# Quinoline-Based Neuropilin‑1 Antagonists Exhibit a Pure Antagonist Profile and Block Vascular Endothelial Growth Factor-Induced Pain

**DOI:** 10.1021/acsptsci.5c00029

**Published:** 2025-10-29

**Authors:** Sara Hestehave, Silvia Dragoni, Philip Fallon, Filipa Mota, Aida Calderon-Rivera, Kimberly Gomez, Jonathan Powell, Anastasia Patsiarika, Tifelle Reisinger, Stuart Crosby, A.W. Edith Chan, David Steadman, Natalie Winfield, Ashley Jarvis, John Martin, Ian C. Zachary, Paul Frankel, Snezana Djordjevic, Christiana Ruhrberg, Rajesh Khanna, David L. Selwood

**Affiliations:** † The Wolfson Institute for Biomedical Research, 4919 University College London, Gower Street, London WC1E 6BT, U.K.; ‡ 105697 NCE Discovery (Domainex Ltd), Chesterford Research Park, Little Chesterford, Saffron Walden, Essex CB10 1XL, U.K.; § Centre for Cardiovascular Biology and Medicine, Division of Medicine, 4919 University College London, 5 University Street ,London WC1E 6JJ, U.K.; ∥ Institute of Structural and Molecular Biology, 4919 University College London, Gower Street, London WC1E 6BT, U.K.; ⊥ Institute of Cardiovascular Science, 4919 University College London, 5 University Street, London WC1E 6JF, U.K.; # UCL Institute of Ophthalmology, 4919 University College London, 11-43 Bath Street, London EC1 V 9EL, U.K.; ∇ Department of Molecular Pathobiology, College of Dentistry, 5894 New York University, New York, New York 10010, United States

**Keywords:** chronic pain, VEGF, antinociceptive, neuropilin, NRP1

## Abstract

Nociceptive pain, resulting from tissue injury or inflammation, affects a large portion of the global population. This type of pain is commonly treated by small molecules that are associated with a variety of drawbacks, including addiction and potential liver or kidney damage, highlighting the need for new therapeutic strategies. Here, we report the design, synthesis, and characterization of EG01449 (**12h**), a quinoline-based neuropilin-1 (NRP1) antagonist with analgesic effects in vascular endothelial growth factor (VEGF)-induced pain models. Neuropilin-1 is a critical coreceptor mediating VEGF signaling. In models of VEGF-induced pain, the VEGFA_165_a isoform increases currents through voltage-gated sodium and calcium channels in dorsal root ganglia sensory neurons. Notably, this effect was mitigated upon the inhibition of NRP1 by **12h,** while **12h** alone showed no discernible impact on sodium currents. Compound **12h** also attenuated sensitivity to mechanical stimuli and cold-induced allodynia. Unlike the previously reported NRP1-targeting compounds that may activate intracellular signaling, **12h** did not activate p38 mitogen-activated protein kinase and exhibited a purely inhibitory pharmacological profile. Structural comparison using X-ray crystallography revealed an additional hydrogen bond that contributes to the increased stabilization of the **12h**/NRP1 complex. These findings demonstrate that the NRP1 inhibitor **12h** elicits an antinociceptive effect and highlight the impact of subtle structural modifications on biological outcomes. NRP1 antagonism thus represents a promising new modality for the treatment of chronic pain conditions.

## Graphical abstract

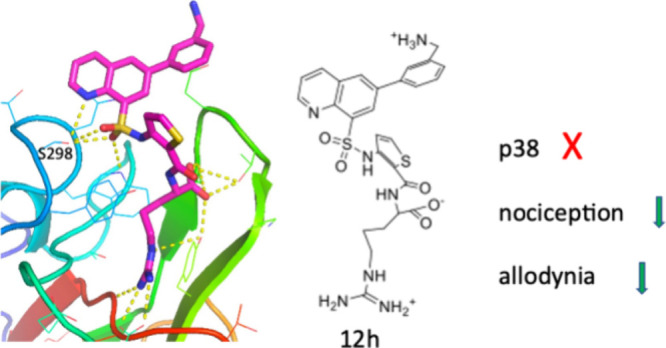

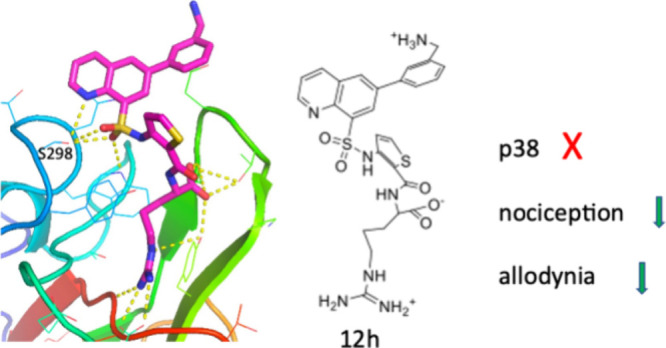

Chronic pain is a global health crisis affecting 11–40% of adults in the US.^1^ Pain is defined as “*An unpleasant sensory and emotional experience associated with, or resembling that associated with, actual or potential tissue damage”*.^2^ The three main types of pain - nociceptive, neuropathic, and nociplastic - differ based on their physiological origin, presentation, and treatment options. Nociceptive pain, usually caused by tissue damage or inflammation, is commonly treated with nonsteroidal anti-inflammatory drugs (NSAIDs) and, in severe cases, opioids.^3^ Neuropathic pain results from nerve damage caused by various factors, including nerve compression or diabetes, and is treated with local injections, surgery, or with central nervous system (CNS)-active drugs. Nociplastic pain involves changes in pain perception and is often linked to chronic pain conditions such as fibromyalgia,^3^ with treatments including CNS-active drugs or nonpharmacological interventions.

Nociceptive pain is the most prevalent form, affecting large segments of the population. However, currently used small-molecule analgesics exhibit multiple problems, including addiction (opioids) or organ toxicity (e.g., liver damage from paracetamol, kidney, and gastrointestinal issues from NSAIDs.^4^ Despite decades of research, new treatments have been slow to appear. A recent innovation, suzetrigine, a specific Nav1.8 channel blocker, is the first nonopioid pain medication to be fast-tracked and approved by the FDA in two decades. Nevertheless, its mechanism of action is also associated with side effects such as itching and muscle spasms, underscoring the need to develop new, safer, and more effective therapeutic approaches. Recent studies have highlighted the role of vascular endothelial growth factor A (VEGFA), its receptors neuropilin-1 (NRP1) and VEGF receptor (VEGFR), and the NRP1/VEGFR signaling axis in pain.^5–7^ VEGFA is pronociceptive, modulating pain-like behaviors in both naïve animals and those with traumatic or diabetic spinal nerve damage.^5,8^ Clinical evidence from patients with osteoarthritis,^200^ where increased VEGFA expression in the synovial fluid is associated with higher pain levels, further supports the view that VEGFA is involved in pain perception. Interestingly, the effects of VEGFA on the sensory nervous system are isoform-dependent: VEGFA_165_b induces antinociception, while VEGFA_165_a promotes nociception.^9^ During pain conditions, the endogenous balance shifts toward the pronociceptive isoform, VEGFA_165_a.^10^ VEGF and its receptors including NRP1 are ubiquitously expressed in several tissues throughout the body including dorsal root ganglia (DRG) neurons which play a vital role in pain perception.^11^ In DRG neurons, upon activation, VEGFA165a increases ion channel current densities and promotes spontaneous firing, resulting in mechanical allodynia and thermal hyperalgesia.^5,12^

NRP1 is a single-pass transmembrane receptor with five extracellular domains (a1, a2, b1, b2 and c) that bind various growth factors, including VEGFA_165_a, and transforming growth factor beta (TGFβ1).^13^ Ligand binding is mediated by a C-terminal amino acid sequence motif RXXR where the terminal arginine is critical for binding to the specific pocket on the NRP1 b1 domain. While VEGFA_165_a, the pronociception-inducing isoform of VEGFA, contains the C-terminal sequence motif critical for interaction with NRP1, the antinociceptive isoform VEGFA_165_b lacks it.^14^ The intracellular C-terminus of NRP1 interacts with PDZ domain-containing proteins (such as the adaptor protein GIPC) via its C-terminal SEA (serine, glutamate, alanine) sequence,^4^ with this protein/protein interaction playing a key role in regulation of vascular permeability.^15^ This positive effect of NRP1 on permeability, dependent on NRP1 expression at adherens junctions^16^ and association with p120 catenin, has been exploited to improve accessibility of nanoparticles and antibodies delivery into tumors.^17^

Targeting NRP1 with small molecule inhibitors may offer therapeutic potential for pain, especially in cases related to conditions such as cancer^18–21^ and chemotherapy-induced pain.^18^ EG00229 (**1**), the first small molecule inhibitor for NRP1, is an arginine derivative with a precise fit for the shallow NRP1 b1 binding pocket, normally occupied by a C-terminal arginine present in most natural ligands.^22^ Despite its modest micromolar potency and relatively short pharmacokinetic duration, EG00229 blocks VEGF signaling^23^ and has shown efficacy in several *in vivo* tumor models.^24–26^ In addition, EG00229 has been effective in alleviating pain-like behaviors after spinal nerve injury,^5^ by preventing VEGFA-induced increase in voltage-gated sodium and calcium channel activity, supporting the therapeutic relevance of NRP1/VEGFA signaling axis inhibition for treatment of pain. While EG00229 inhibited VEGFA-induced permeability in primary brain endothelial cells and retinal blood vessels, it also exhibited agonist-like properties by activating NRP1-dependent signaling pathways that regulate the vascular barrier.^27^ Specifically, EG00229 induced the NRP1-dependent phosphorylation of p38 mitogen-activated protein kinase (MAPK) at T180/Y182, a hallmark of VEGFA-induced permeability signaling in brain and retinal endothelial cells.^28–30^

Here we aimed to improve on the activity and pharmacokinetic profile of EG00229 by designing novel inhibitors with a different pattern of interactions with NRP1, and critically to assess their potential for p38 activation. These compounds were evaluated across multiple assays and compared to EG00229. In contrast to EG00229, our newly developed quinoline-based molecules with additional hydrogen-bonding capability, did not activate p38 kinase or induce vascular permeability, yet produced significant reversal of pain-like behaviors in rodents. NRP1 inhibitors intended to block pain signaling should ideally exhibit a purely inhibitory effect on the p38 kinase pathway.^31^ Furthermore, the new compounds demonstrated improved pharmacokinetics compared to EG00229, offering a distinct mechanism of action and a potential therapeutic profile for pain management.

## Results and Discussion

### New Chemistry Design

In our previous studies on benzothiadiazole-based NRP1 ligands we noted that in crystal structures hydrogen bonding from the heteronitrogen on the benzothiadiazole to S298 within the NRP1 b1 domain ligand-binding site was only seen in one of the two protein chains (chain B, PDB ID: 3I97).^23^ Benzothiadiazole is considered a highly electron deficient heterocycle and is often used in organic electronics in push–pull materials.^32^ Furthermore, a survey of the pdb revealed only five benzothiadiazole – protein structures of these only EG00229 (pdb: 3I97) displayed a H-bond to the protein (Table S1). Similarly, hydrogen bonding (H-bonding) potential to the oxygen heteroatom was observed in a low resolution (6FMF) but not in a high resolution (6FMC) crystal form for a NRP1-bound dihydrobenzofuran analogue (**2)** (Figure 1A–C).^33^ We hypothesized that introduction of a stronger H-bond acceptor within the ligand would maximize H-bond interactions to S298 on NRP1 and potentially improve the affinity.

**Figure 1:**
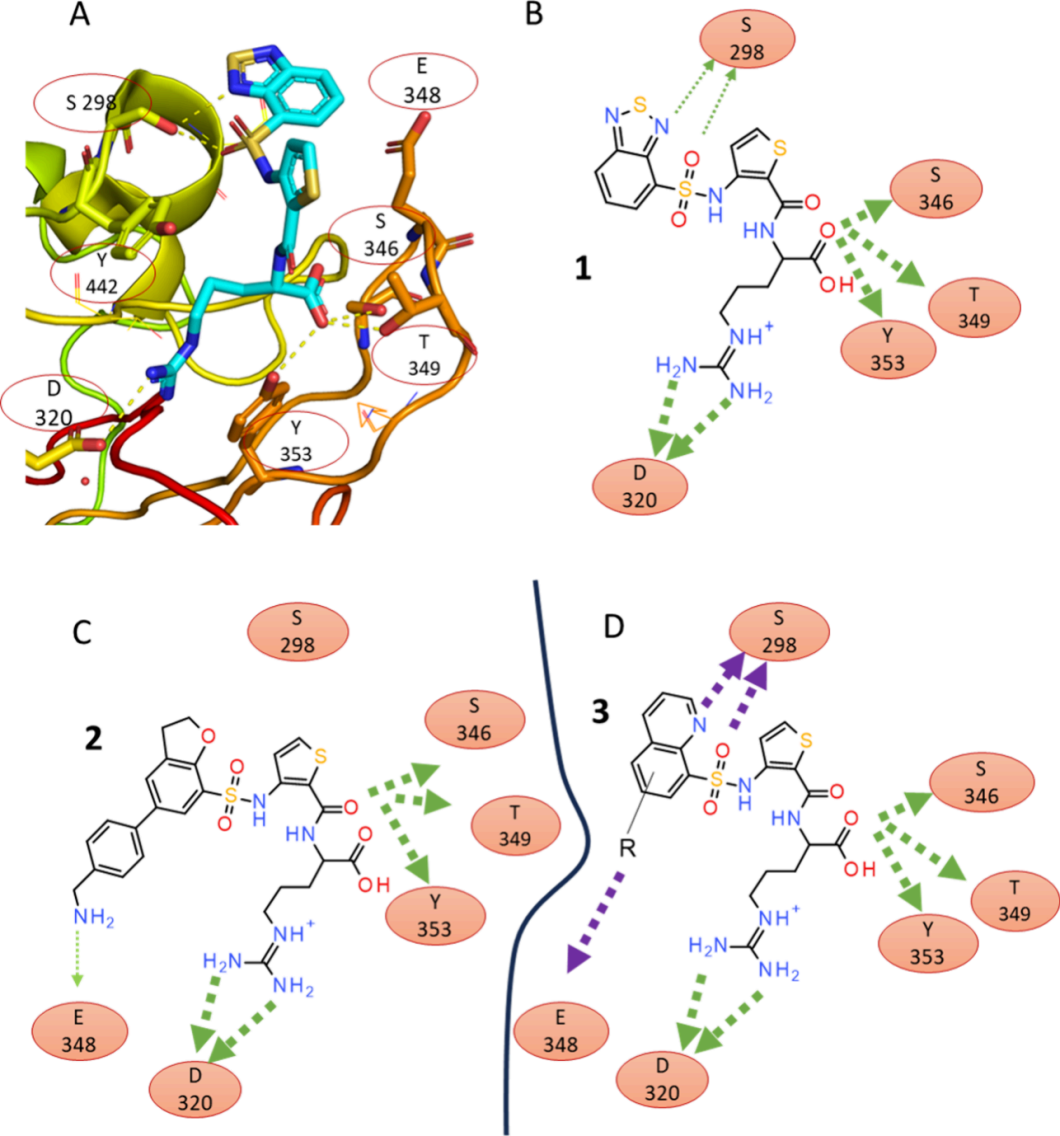
Design of quinoline-based NRP1 ligands. (A) View of the EG00229 (**1**) in a binding site within the structure of EG00229-bound NRP1 (PDB ID: 3I97 chain B) showing key interactions. The structure of EG00229 is shown as a stick model with carbon, nitrogen, oxygen and sulfur atoms colored in light blue, dark blue, red and yellow, respectively. (B) Schematic diagram of interactions based on panel A. Hydrogen bonds of the benzothiadiazole to S298 are only visible in the B chain of EG00229-bound NRP1 (thin green arrows). (C) Diagram of the dihydrobenzofuran ligand showing interactions identified in the crystal structures of the NRP1-bound complex (PDB ID: 6FMC). (D) Diagram of designed quinoline showing expected interactions to the residues of NRP1 (green) and hypothetical interactions in magenta.

Given the higher H-bond strength expected for quinoline and its synthetic accessibility we selected quinoline analogues as a new target set. We proposed that 2-quinoline-based structures (**3**) (Figure 1D) would form a stronger hydrogen bond compared to previously reported molecules. Estimates of hydrogen bond acceptor strength using the p*K*_BHX_ scale^34^ place aromatic amines such as quinoline at 1.89 while the oxygen-containing tetrahydrofuran has p*K*_BHX_ value of 1.28. For comparison, a weak H-bond acceptor, diethyl ether, scores 1.01 on the same scale, while the strong H-bond acceptor imidazole is at 2.72. Since quinolines can be functionalized, this modified scaffold might offer a robust platform for exploring other interactions with NRP1 and targeting additional surface residues, such as E348 (Figure 1D).

### Synthesis

The synthesis of the quinoline target compounds began from a common brominated intermediate prepared as shown (Scheme 1). 6-Bromoquinoline 4 was reacted with chlorosulfonic acid under forcing conditions (160 ^o^C) to produce the sulfonyl chloride 5 in a poor yield (10–15%). The poor yield was representative of many trials of this transformation and reflects the deactivated nature of the quinoline system. The arylsulfonyl chloride **5** was then reacted with methyl, 3-aminothiophene-2-carboxylate to give the sulfonamide **6**. Hydrolysis with LiOH, H_2_O, and THF to **7** also required forcing conditions (140 °C, sealed tube) to provide the product. Finally, reaction with Pbf-protected arginine methyl ester required the highly active coupling agent PyBrOP,^3^ but proceeded smoothly to give the protected arginine derivative, **8** which was hydrolyzed to the desired quinoline – thiophene-arginine scaffold intermediate **9**.

**Scheme 1:**
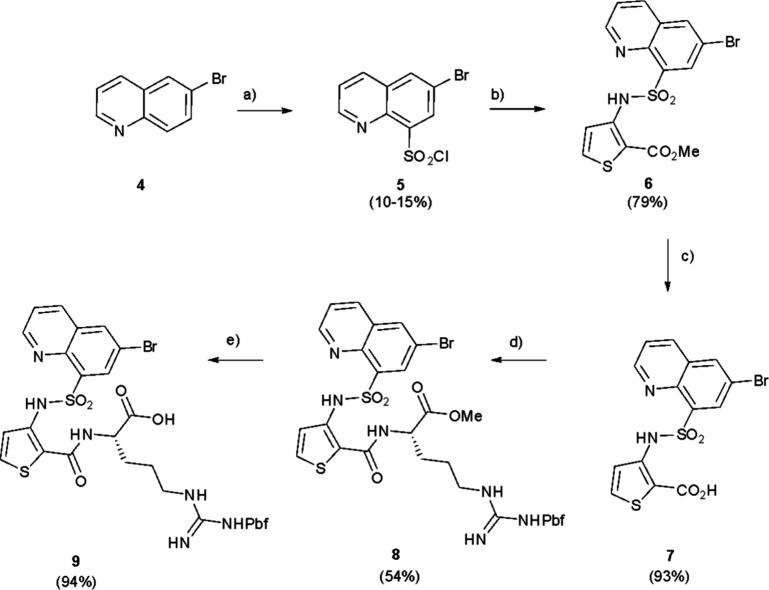
Synthesis of Brominated Intermediate **9** ^a^

a Reagents and conditions: (a) chlorosulfonic acid, 160 °C, 18 h; (b) methyl, 3-aminothiophene-2-carboxylate, pyridine, 0 °C – rt, 20 h; (c) LiOH·H_2_O, THF, H_2_O, sealed tube, 140 °C, 2 h; (d) H-l-Arg­(Pbf)­OMe, PyBrOP, DIPEA, CH_2_Cl_2_, 10 °C – rt, 16 h; (e) LiOH·H_2_O, THF, H_2_O, rt, 4 h.

Several different procedures were then adopted to produce a range of analogues. In the first route (Scheme 2), analogues were prepared by a Suzuki coupling of the relevant aryl boronic acid using palladium tetrakistriphenylphosphine with the quinoline bromide **9** to give **10a,b** followed by a reductive amination with a suitable amine to give **11a**–**c**. Subsequent deprotection gave the desired compounds **12a**–**c** (Scheme 2). In contrast (Scheme 3) a direct Suzuki coupling of the relevant boronic acid onto **9** gave the protected compounds **11d**–**f**. Again, deprotection gave the targets **12d**–**f** (Scheme 3).

**Scheme 2:**
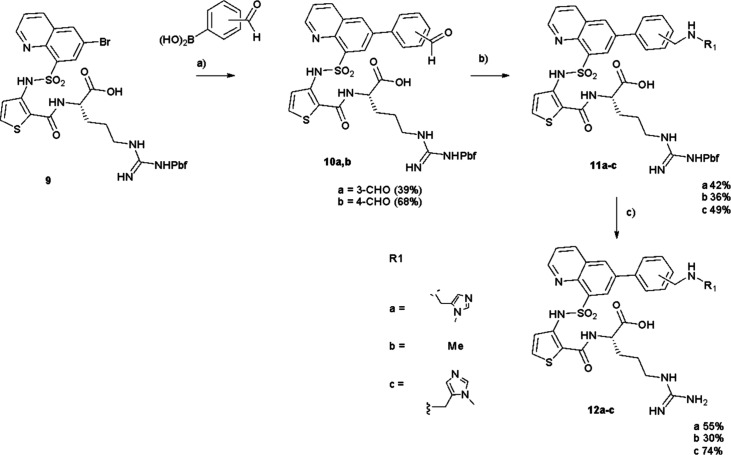
Synthesis of **12a–c** ^a^

a Reagents and conditions: (a) Pd­(PPh_3_)_4_, K_3_PO_4_, THF, H_2_O, MW, 90 °C, 30 min; (b) R_1_-NH_2_, NaCNBH_3_, AcOH, THF, MeOH, 0 °C - rt, 4 h; (c) TFA, CH_2_Cl_2_, rt, 20 h.

**Scheme 3:**
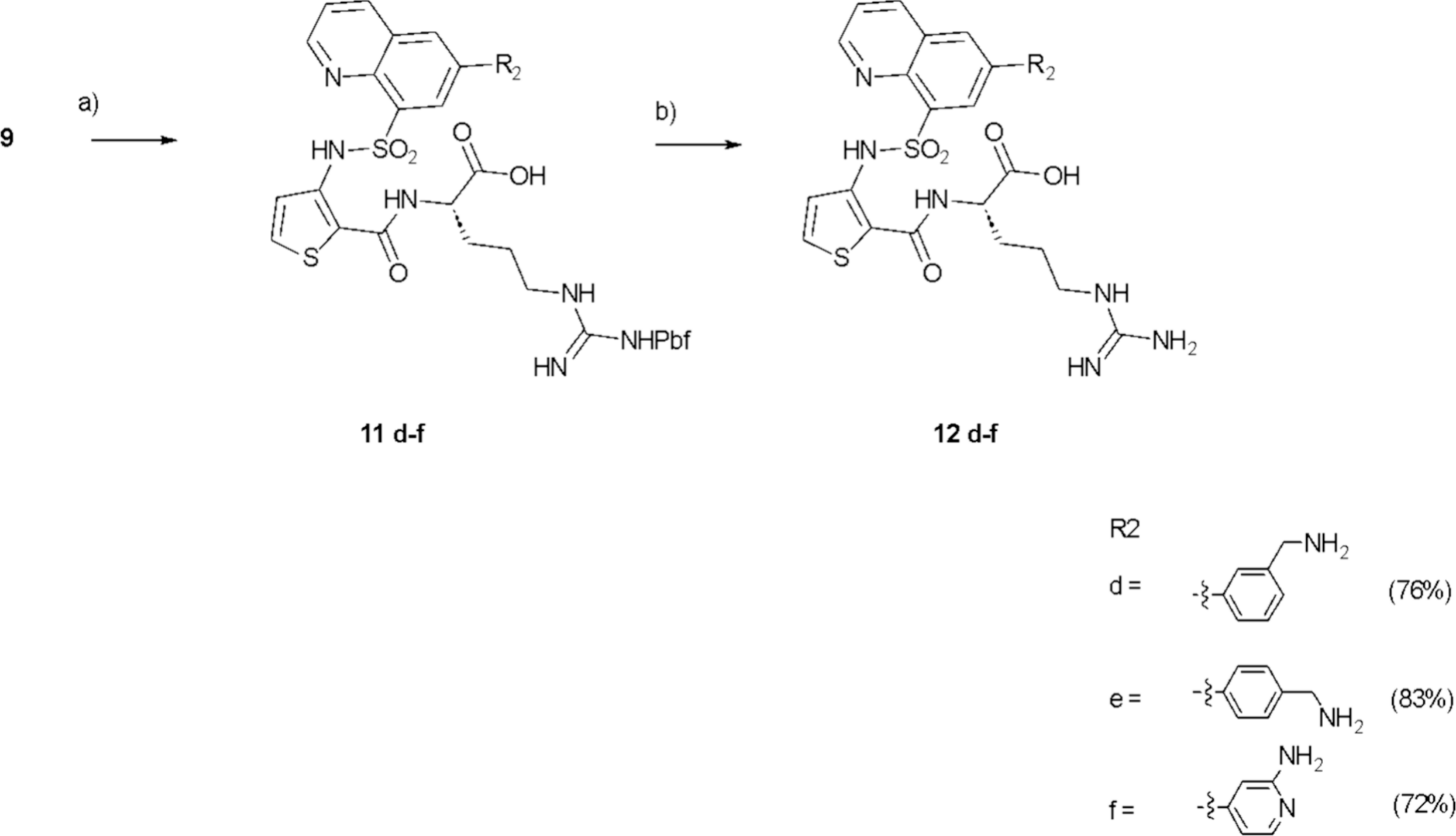
Synthesis of **12d–f** ^a^

a Reagents and conditions: (a) R_2_B­(OH)_2_, Pd­(PPh_3_)_4_, K_3_PO_4_, THF, H_2_O, *M*_W_, 130 °C, 30 min; (b) TFA, CH_2_Cl_2_, rt, 20 h.

Direct N-linked compounds were prepared from **9** via an initial amination reaction to give amine **13**, followed by subsequent reductive amination with the desired aldehyde to give the protected intermediates **11g–11n.** Subsequent deprotection gave the final products, **12g**–**n** (Scheme 4). Compound **12o** was synthesized using a similar method as for **9** but starting from commercially available 8-quinolinesulfonyl chloride **14** which was coupled to methyl, 3-aminothiophene-2-carboxylate to give **15.** Ester hydrolysis gave **16** which could then be coupled to protected arginine to give **17.** Further ester hydrolysis gave **11o** which was then further deprotected to give **12o** (Scheme 5).

**Scheme 4:**
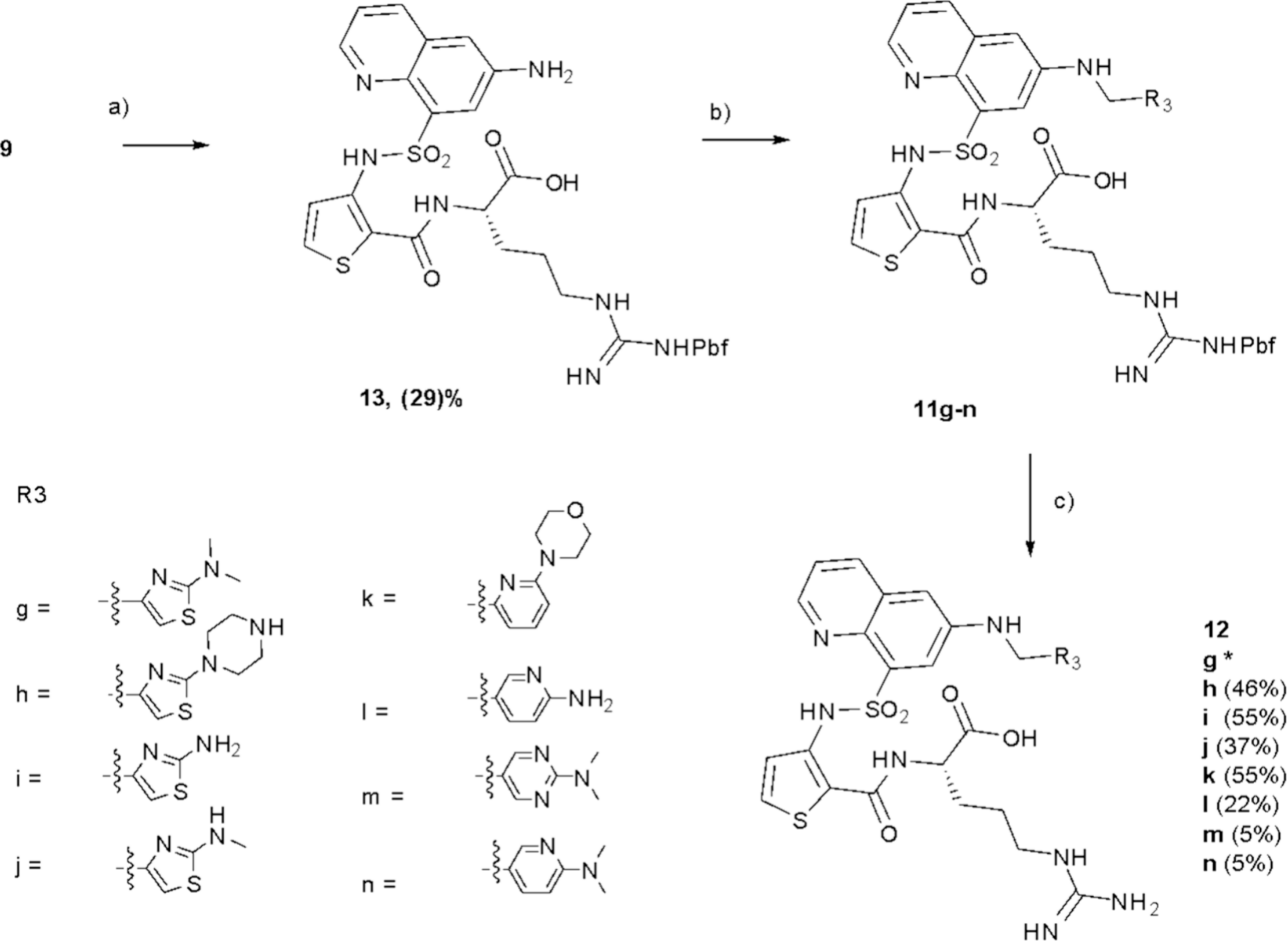
Synthesis of **12g–n** ^a^

a Reagents and conditions: (a) NaN_3_, DMEDA, CuI, Na_2_CO_3_, DMSO, 110 °C, 1 h; (b) R_3_-CHO, NaCNBH_3_, AcOH, THF·MeOH, 0 °C - rt, 4 h; (c) TFA, CH_2_Cl_2_, rt, 20 h.

**Scheme 5:**
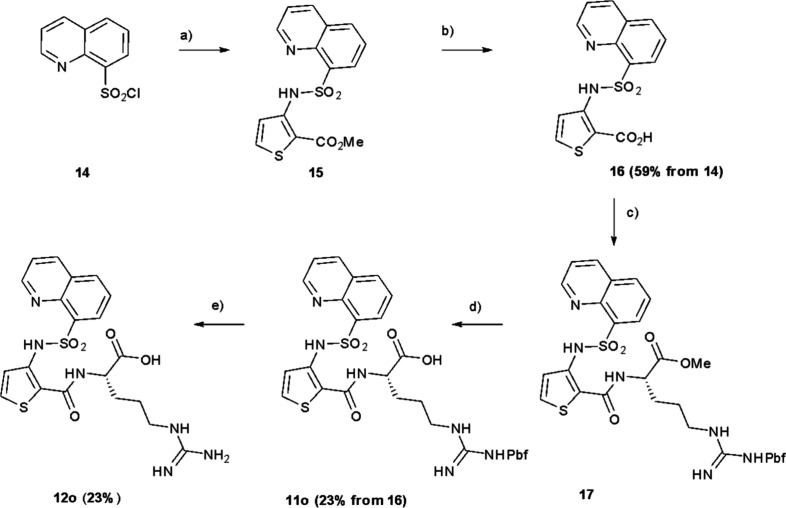
Synthesis of **12o** ^a^

a Reagents and conditions: (a) methyl 3-aminothiophene-2-carboxylate, pyridine, 0 °C – rt, 20 h; (b) LiOH, THF·MeOH·H_2_O, 65 °C, 5 h; (c) H-l-Arg­(Pbf)-OMe, HATU, DIPEA, CH_2_Cl_2_, rt, 18 h; (d) LiOH·H_2_O, THF, H_2_O, rt, 4 h; (e) TFA, CH_2_Cl_2_, rt, 20 h.

**12p** and **12q** were prepared by converting **6** into the corresponding boronic acid **19**, using the Pd­(dppf)_2_Cl_2_ catalyst (Scheme 6). Subsequently, Suzuki coupling of **19** with the appropriate aryl bromide was performed to produce **20p,q**. Ester hydrolysis gave **21p,q** which could then be coupled to protected arginine to give **22p,q.** Further ester hydrolysis gave **11p,q** which could then be deprotected to **12p,q** (Scheme 6).

**Scheme 6:**
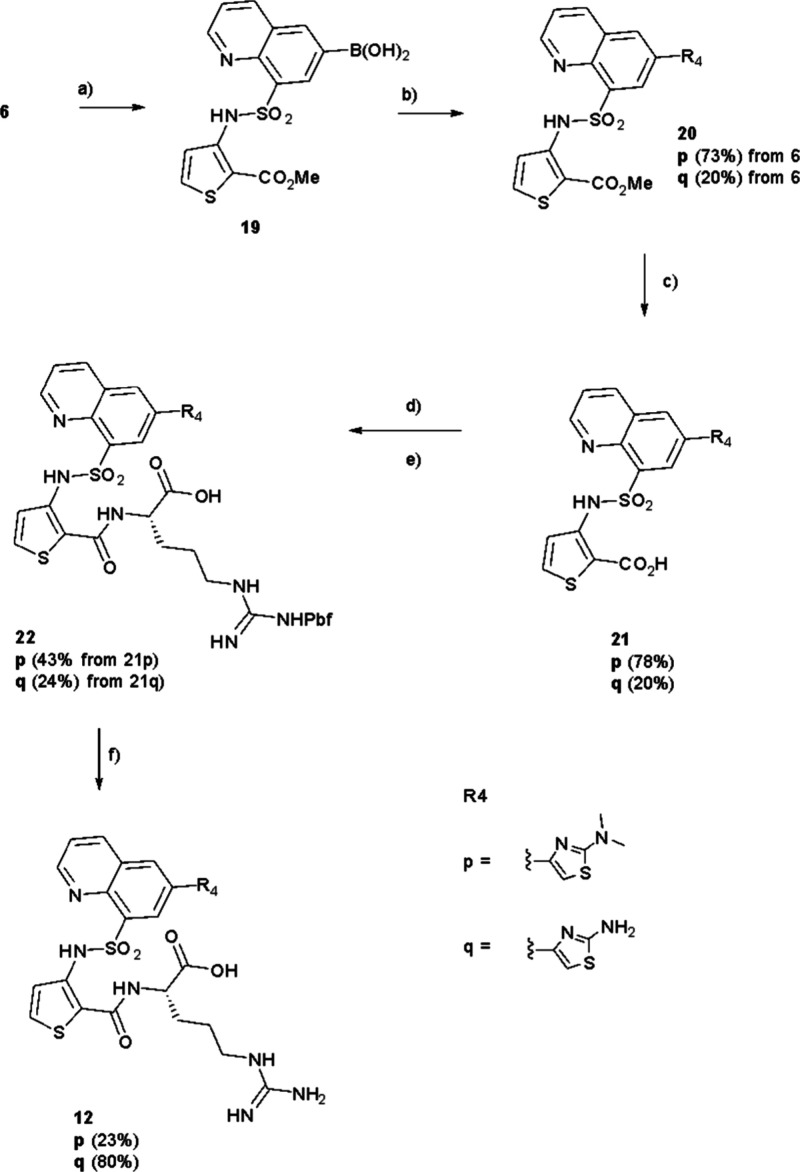
Synthesis of **12p,q** ^a^

a Reagents and conditions: (a) bis­(pinacolato)­diboron, Pd­(dppf)_2_Cl_2_, KOAc, dioxane, *M*_W_ 100 °C, 10 min; (b) R4-Br, Pd­(PPh_3_)_4_, K_3_PO_4_, DME·H_2_O, *M*_W_ 120 °C, 20 min; (c) LiOH, THF·H_2_O·MeOH, 50 °C, 16 h; (d) H-l-Arg­(Pbf)­OMe, PyBrop, DIPEA, CH_2_Cl_2_, 10 °C – rt; (e) LiOH·H_2_O, THF, H_2_O, rt, 4 h; (f) TFA, CH_2_Cl_2_, rt, 20 h.

To establish a scalable route to **12h** we investigated ways to avoid the problematic sulfonation reaction. After many trials, a workable route was established from commercially available 6-nitroquinoline **23** (Scheme 7) through regioselective bromination in concentrated H_2_SO_4_ followed by reduction using iron powder and Boc formation to generate the intermediate **24** (89% yield over three steps).^35^ A three-step sequence^36^ of palladium-catalyzed thiolation, oxidation and sodium ethoxide promoted elimination was used to generate the sodium sulfinate derivative **25**. At this point, reaction under mild conditions using iodine as oxidant^37^ and the thiophene amine gave the key sulfonamide intermediate **26**. The synthesis now proceeded using similar methodology to that already described, thus reductive amination to **27** with deprotection and amide HATU coupling gave fully protected precursor **28**, which yielded **12h** on full deprotection (Scheme 7).

**Scheme 7:**
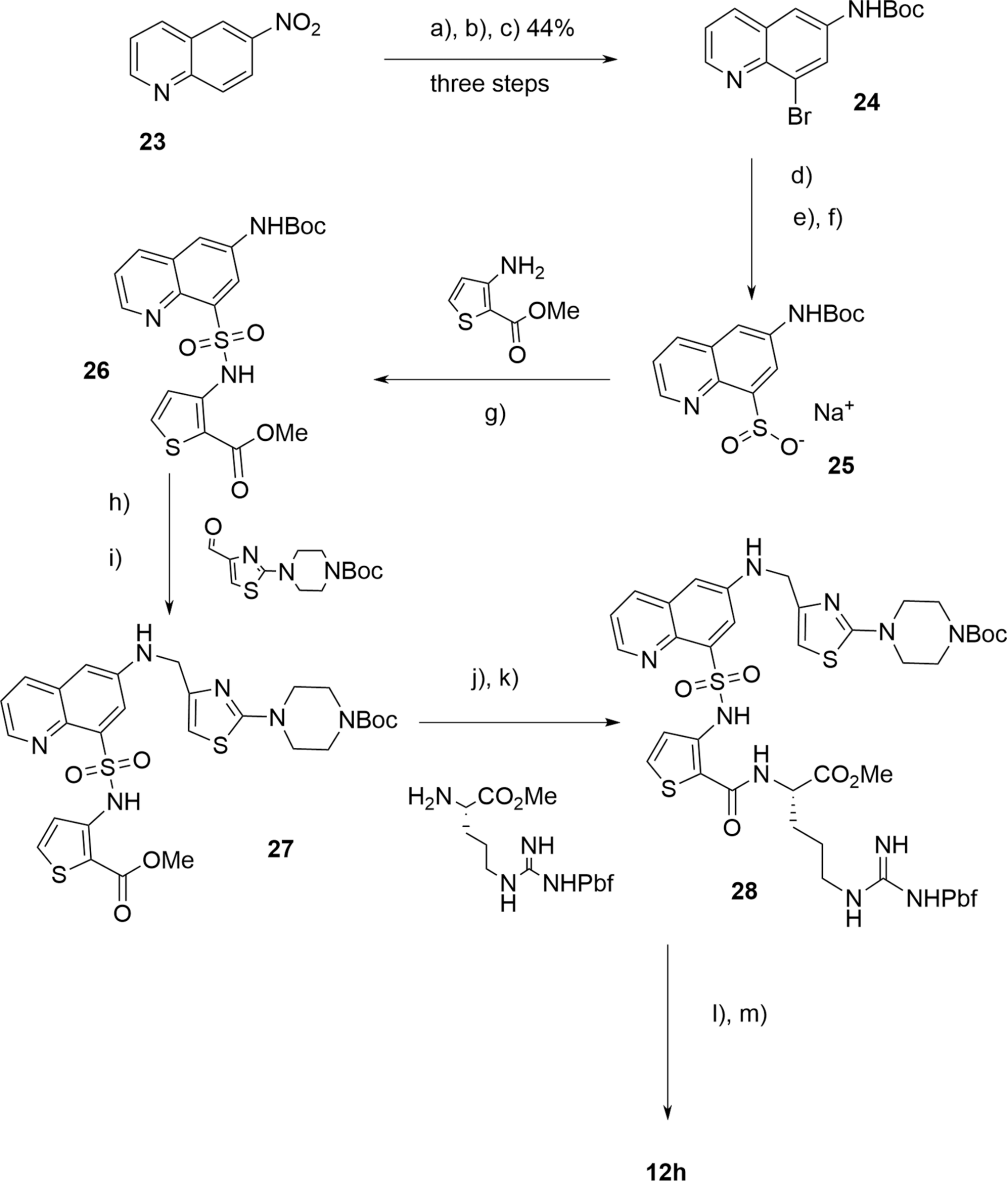
Alternate Synthesis of **12h** ^a^

a Reagents: (a) NBS, (b) Fe NH_4_Cl, (c) (Boc)_2_O, (d) Pd_2_(dba)_3_, Xantphos, toluene, (e) Oxone, ACN, H_2_O, (f) NaOEt, MeOH (g) I_2_, EtOH, (h) TFA/TIPS/DCM, (i) BH_3_, DCM, (j) LiOH, THF·MeOH·H_2_O, 65 °C, 5 h; (k) PyBrOP, DCM, (l) LiOH, THF, rt, (m) 50% TFA/DCM.

Thus, a range of synthetic strategies allowed access to the quinoline target molecules consistent with the design parameters described above.

### Biophysical and Structural Evaluation

#### NRP1 Binding and Competition Studies against VEGFA

The binding affinities of all newly synthesized compounds in the series (**12a–q)** for NRP1 were assessed using surface plasmon resonance (SPR), with the purified recombinant NRP1 b1 domain immobilized on the chip (Table 1). As the SPR is effectively a stop-flow instrument it enables the study of on–off kinetics and determination of the association and dissociation constants. Slow off-rates are linked to residence time and are considered to be beneficial for small molecule drugs.^38^ All quinoline compounds showed consistently good affinities for the NRP1 b1 domain, ranging from 2.54 to 0.32 μM. The unsubstituted quinoline **12o** had a modest dissociation constant (*K*_D_) of 1.40 μM. Quinoline-aryl compounds **12d–f** demonstrated approximately double the potency of the unsubstituted quinoline, with **12d** - the 4-aminomethyl compound - showing the best affinity for NRP1 (*K*_D_ = 0.65 μM), possibly indicating interaction with additional residues on the NRP1 protein surface, suggesting a potential “out-of-pocket” interaction. Quinoline aminomethylheteroaryls **12g–n** showed superior potency, with aminomethyl-thiazolyl derivatives achieving submicromolar affinities: 0.60 μM for **12h** and 0.53 μM for **12j**. Unfortunately, **12g** exhibited poor solubility, and SPR data fitting for this compound did not converge. Quinoline-heteroaryl compounds **12p,q** exhibited a marked difference in affinity: the free heteroamine **12q** was much more affine with a *K*_D_ of 0.51 μM, compared to 2.54 μM for the dimethylated **12p**. The most potent compounds were around 10-fold more effective than our standard inhibitor EG00229 (*K*_D_ ∼ 3.20 μM), suggesting that these new compounds could indeed be interacting with E348, as predicted. Compound **12h** exhibited equilibrium binding kinetics in SPR, with rapid association and slower dissociation rates and an excellent full dose response curve (Figure 2A–C). Selected compounds (**12g, 12h** and **12j**) were further evaluated in an orthogonal, plate-based, cell-free competition assay involving displacement of biotinylated VEGFA_165_a (bt-VEGFA_165_a). All demonstrated potent activities with **12h** emerging as the best compound overall in this system. Figure 2D shows data for the binding analysis of **12h** and confirms **12h** as an effective competitive inhibitor of VEGFA binding to NRP1 (Note: Unless specified otherwise, for simplicity, VEGFA refers to VEGFA_165_a isoform in all figures and legends).

**Figure 2:**
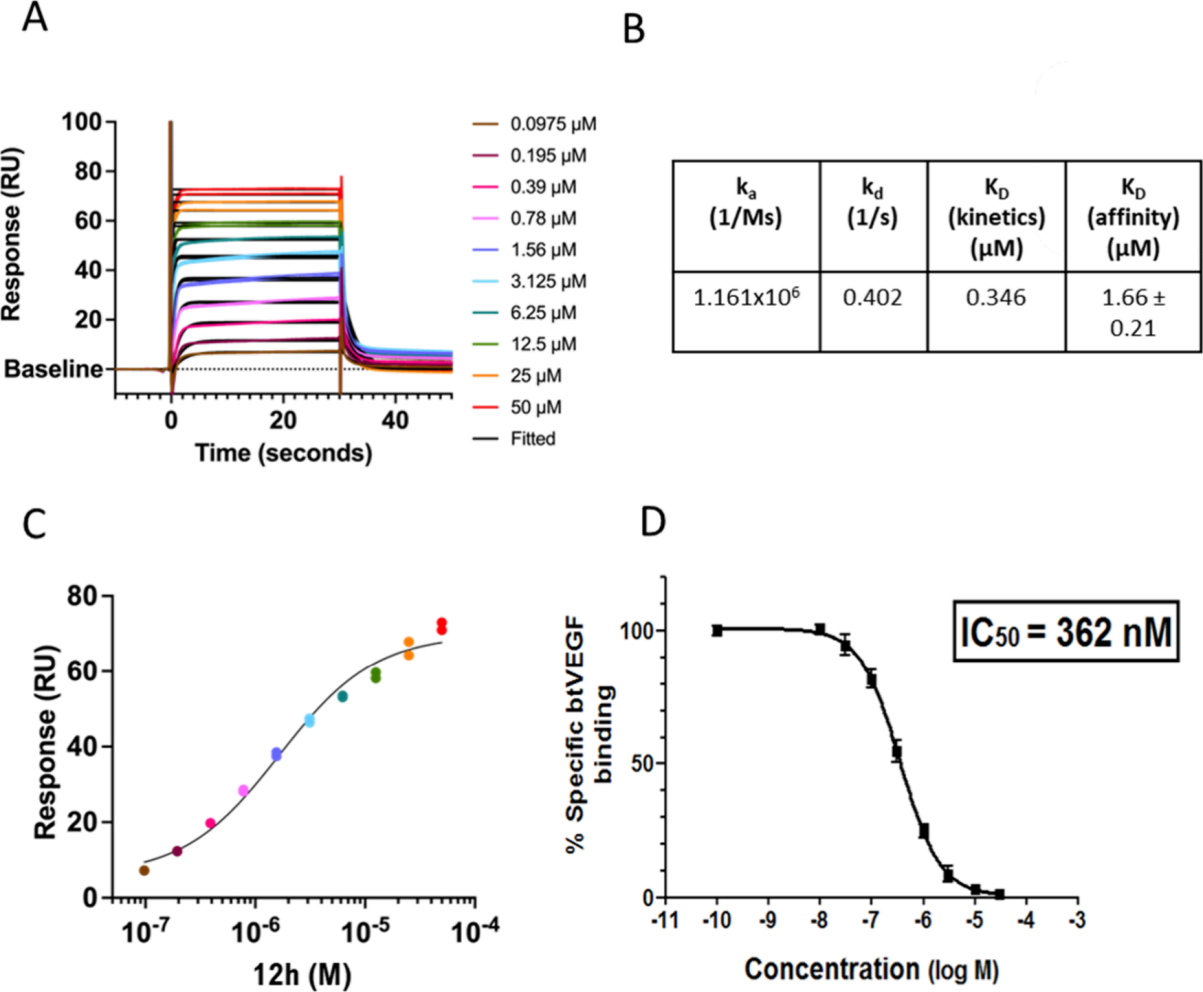
Binding affinity assays for **12h**. (A) SPR Sensorgram of **12h** binding to NRP1 b1 domain immobilized on a CM5 chip at different concentrations as shown. (B) SPR-derived binding parameters for **12h**. (C) Dose response analysis based on equilibrium binding experiment. (D) Competitive binding activity of **12h** with bt-VEGFA to NRP1 b1 domain in a plate-based assay.

**Table 1:**
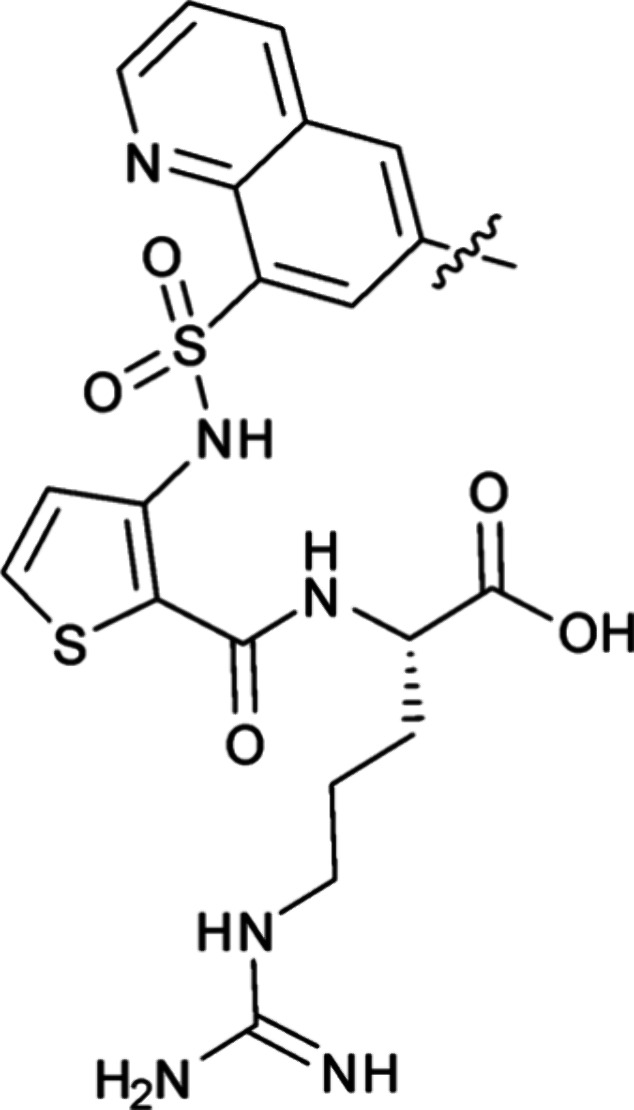

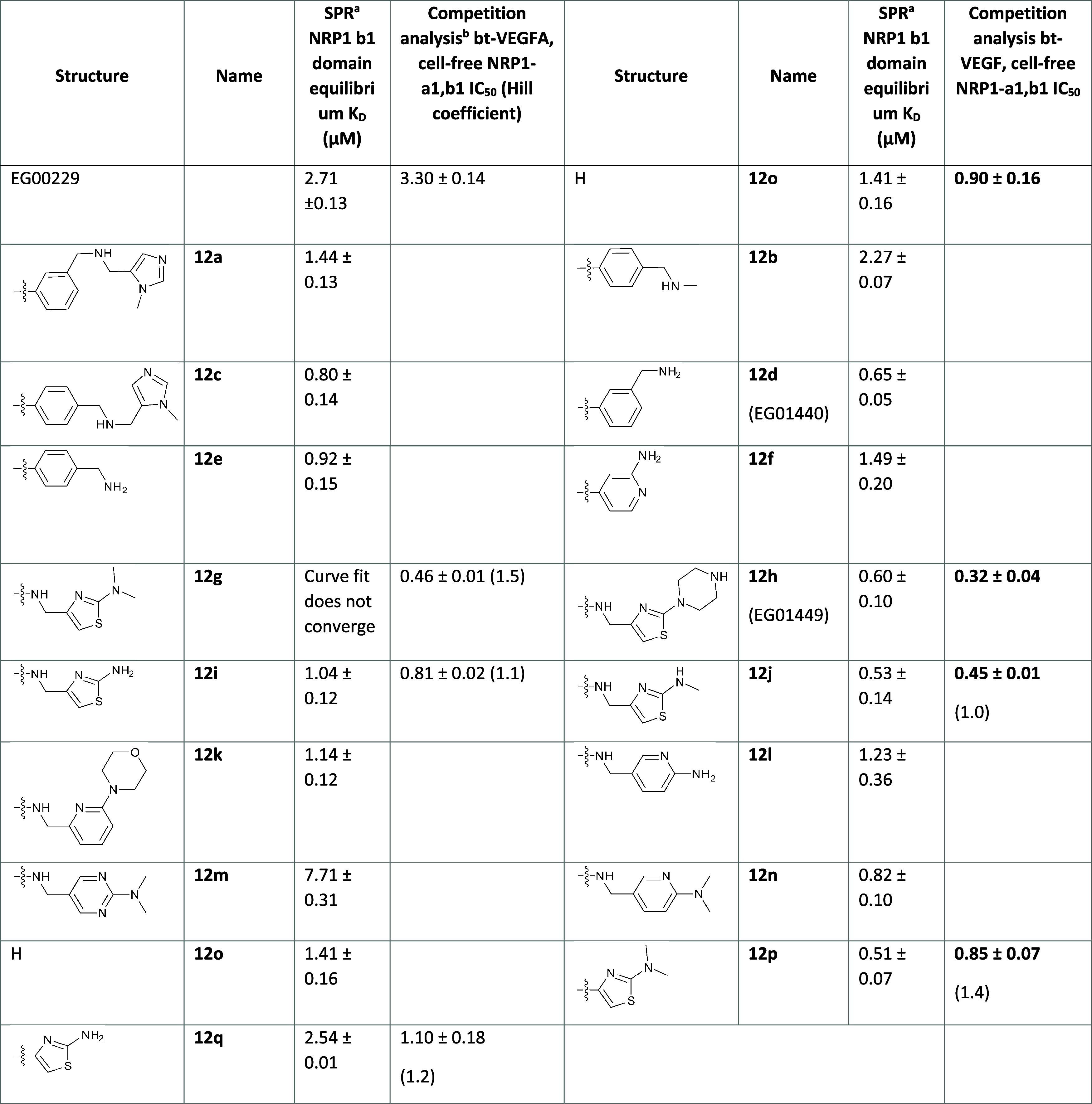
SPR Derived Equilibrium Binding Constants for **12a–q**

a Data is the mean ± SD.

b Data is the mean ± the SEM and is the result of at least three independent experiments.

These assays demonstrated that improved binding and competition potencies were achieved by the strategic replacement of the heterocycle with quinoline.

#### Crystallographic Studies of **12d** Reveal H-Bond from Quinoline Nitrogen to S298

To confirm new interactions between quinoline-based compounds and the NRP1 b1 domain, crystallization screens were set up for the complexes of NRP1 with the range of compounds. We obtained crystals of NRP1 b1 domain in complex with **12d**, one of the best binders (*K*_D_ = 0.65 μM), and X-ray diffraction data were collected on this crystal. Diffraction data and the refinement statistics for the structure (PDB ID: 9F6B) are provided in the Supporting Information (Table S2). The data revealed that the protein/ligand complex crystallized in a monoclinic space group with two protein chains per asymmetric unit (labeled Chains A and B), each bound to a single molecule of **12d**. In the crystal structure the protein molecules are packed such that the ligand binding site in chain A is positioned near the interface with the protein chain B, and vice versa. The binding mode of **12d** resembled that previously observed for EG00229 ^23^ (PDB ID: 3I97). The ligand-binding site is formed by protein loops atop the β-sandwich of the discoidin structural domain with the arginine moiety occupying a pocket defined by, Y297, D320, S346, T349, and Y353, of NRP1 (Figure 3). Interactions with S298 are evident indicating the expected improvement in H-bonding.^39^

**Figure 3:**
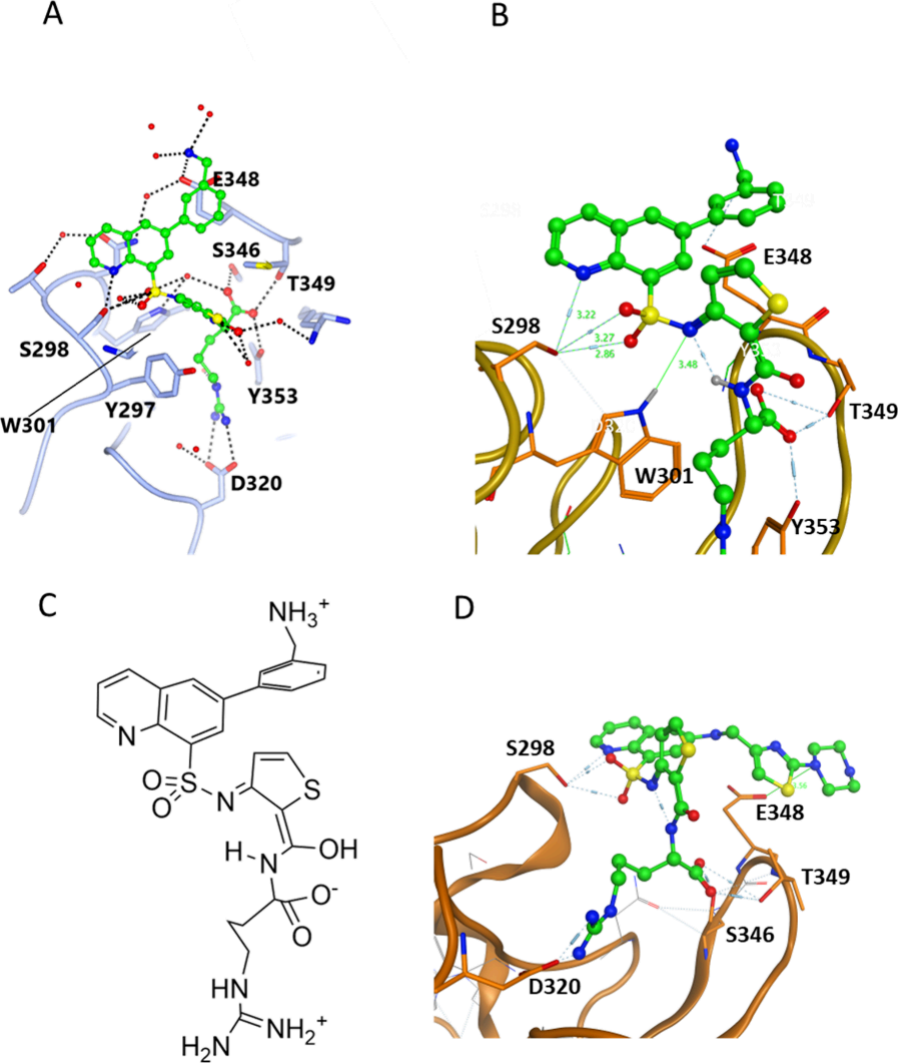
X-ray structure of 12d (PDB ID: 9F6B). Compound **12d** (carbon atoms shown in green in panels A and B) makes extensive hydrogen-bond contacts with NRP1 residues and bound water molecules. (A) **12d** bound to A chain (Figure S1 - **12d** bound to B chain). The hydrogen bonds from the quinoline nitrogen to S298 are visible in both chains within the asymmetric unit. In chain A, an out-of-pocket interaction with E348 is observed but not in chain B where the aromatic ring is rotated. (B) Close-up view of the ligand binding site in chain B showing detail of S298 hydrogen bonds and the interaction of **12d** with W301. (C) Line drawing of **12d** in same orientation as panel A, showing the bound tautomer. (D) Docked conformation of **12h**. In the ball-and-stick model, carbon, oxygen, nitrogen and sulfur atoms are colored green, red, blue and yellow, respectively.

Both ligands form two interactions: one between the guanidine moiety and the side chain of D320, and another with the backbone oxygen of I415. Additionally, they form hydrogen bonds from the acidic group to the hydroxyl groups of Y353, T349, and S346. However, in contrast to EG00229 the quinolinium nitrogen in **12d** was positioned within hydrogen bond range with S298 in both crystallographic protein chains. The distances were 3.22 Å between S298 O and quinoline N, and 3.27 and 2.85 Å between S298 O and sulfonamide oxygens (Figure 3B). The hydrogen bond range is generally considered to be 2.2–3.5 Å, with shorter distances indicating stronger bonding. This observation supports the design rationale of using quinoline nitrogen as a more effective H-bond acceptor. The crystal structure also revealed that the bioactive conformation for the thiophene amide in **12d** adopts a tautomeric structure, stabilized by an intramolecular hydrogen bond, enabling a potential additional interaction with W301, as shown in Figure 3B,C.^40^

Interestingly, two different binding poses were observed for the terminal benzylamine group of **12d**: one with the amino group (NH_3_^+^, Figure 3) rotated away from E348 (Supplementary Figure S1), and another pose where additional H-bond interactions were evident (Figure 3A). We considered that the stronger, more consistent H-bond to the quinoline nitrogen combined with a potential out-of-pocket interaction likely contributes to the increased potency of the quinoline series. As the repeated attempts at crystallization of a NRP1 complex with **12h** were unsuccessful, a computational docking study was performed. The docked model (Figure 3D) shows the pendant thiazolyl- piperazine group largely projecting into solvent, with a potential interaction with E348 easily accommodated.

Taken together, these results suggest a more consistent H-bonding pattern in the crystal structure of the quinoline-type inhibitors and support the interpretation that quinoline increases hydrogen-bonding propensity, contributing to improved binding affinity.

### Biological Evaluation

#### In Vivo Pharmacokinetic and Stability Studies Identify **12h** as a Lead Compound for Further Studies

In addition to enhancing potency, we aimed to improve the pharmacokinetic properties of NRP1 inhibitors. Thiazole analogues **12g**, **12h**, and **12j** were selected for pharmacokinetic analysis because they exhibit competitive inhibition of bt-VEGFA binding to NRP1 at concentrations below 500 nM. Compounds were administered intravenously at 2 mg/kg in mice (Table 2). Among the new quinoline based thiazole set, **12h** displayed the most favorable profile with the lowest clearance (24.50 mL/min/kg), highest AUC (1367 ng*h/mL), and longest half-life (1.30 h). For comparison, the half-life of EG00229 was 0.58 h.^41^ This study demonstrated that biologically relevant exposures were achievable with the quinoline series, and compound **12h** was selected for further biological evaluation.

**Table 2:**
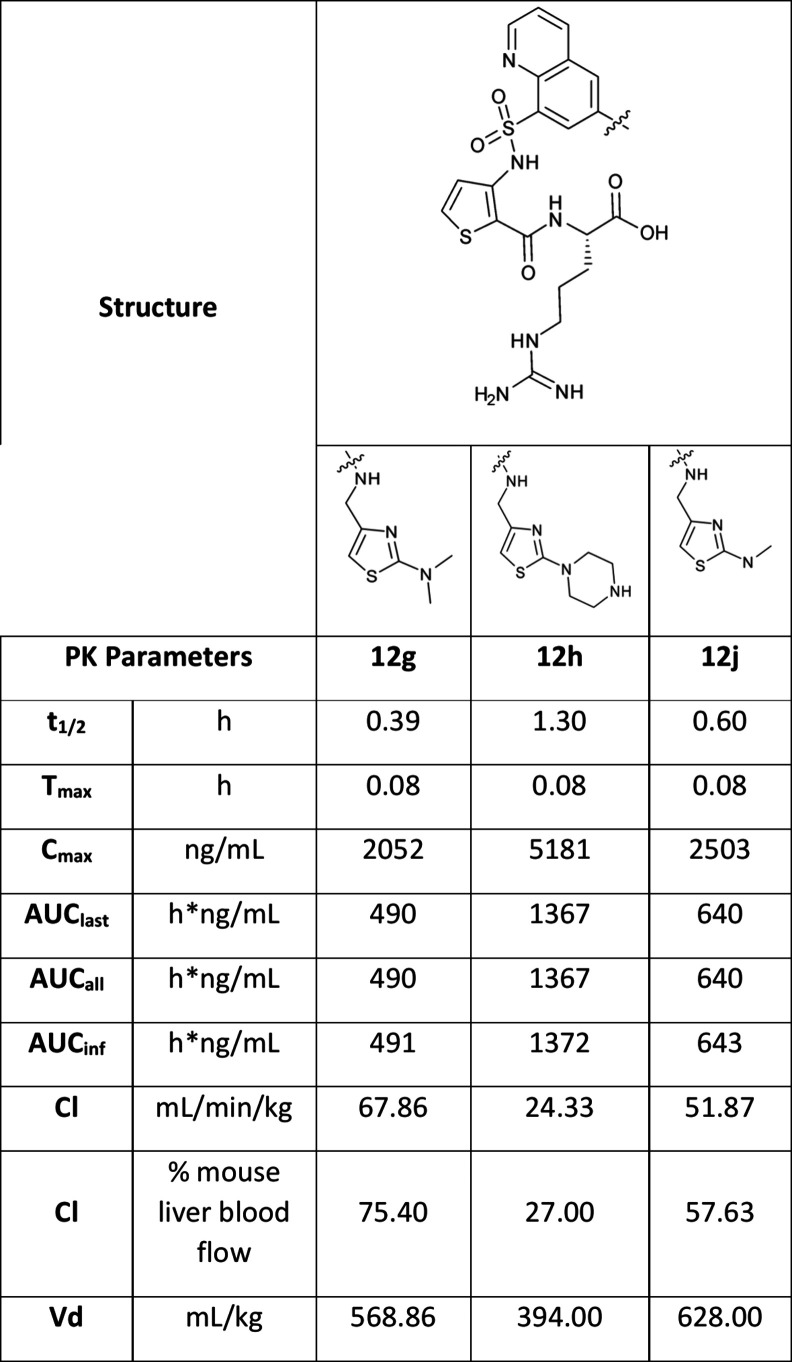
Pharmacokinetic Parameters for the Thiazole Compounds in Mice (Dosed at 2 mg/kg/iv)

#### Effect of **12h** on VEGFA Signaling in Retinal (Ex Vivo) and Brain Endothelial Cells (In Vitro) and on VEGFA-Induced Vascular Leakage in Ex Vivo Mouse Retinas

NRP1 has been shown to mediate VEGFA-induced activation of p38 kinase in endothelial cells, an important pathway in pain signaling. After demonstrating that 12h binds directly to the NRP1 b1 domain and acts as a competitive inhibitor of VEGFA, we evaluated the effects of **12h** and EG00229 on VEGFA165-induced, NRP1-dependent p38 kinase activation in established models—vascular endothelial cells of the ex vivo mouse retina and human brain endothelial cells. We also assessed the downstream induction of vascular permeability in the retina. First, we incubated *ex vivo* retina with **12h** (30 μM) or EG00229 (30 μM) for 15 min prior to VEGFA_165_ stimulation. Whole mount staining for T180/Y182 phosphorylated p38 (P-p38) together with the vascular endothelial marker isolectin B4 (IB4) showed that both **12h** and EG00229 prevented VEGFA-induced p38 phosphorylation in retinal endothelium (Figure 4A,B). In addition to the desired inhibitory effect on VEGFA signaling, EG00229 also induced p38 phosphorylation after 5 min when added alone, (Figure 4A,B). In contrast, **12h** on its own did not induce p38 phosphorylation after 5 min of incubation (Figure 4A,B), suggesting that its mechanism of action may differ from EG00229 by lacking this activation ability.

**Figure 4:**
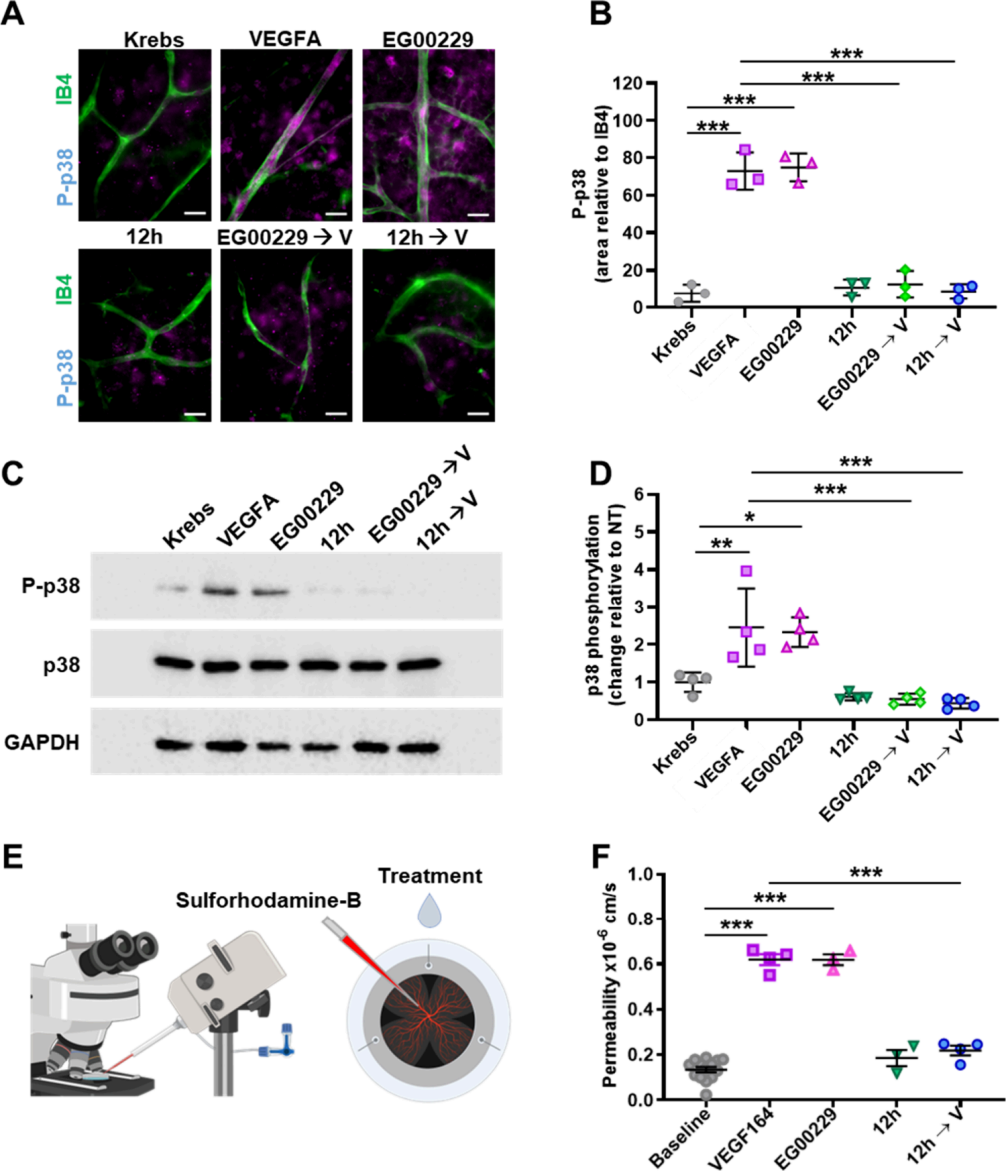
12h inhibits VEGFA-induced permeability and signaling. (A, B) Freshly dissected retinae from C57Bl/6J mice were incubated in Krebs solution with or without VEGFA, EG00229, or **12h**, or were preincubated with EG00229 or **12h** (30 μM) for 15 min before adding VEGFA. Ex vivo retinae were then fixed and immunostained with the vascular endothelial marker isolectin B4 (IB4, green) and an antibody against phosphorylated p38 (P-p38, magenta). (A) Epifluorescent images (scale bars: 20 μm) were used for quantification. (B) Pixel intensity for P-p38 in the IB4-positive vascular area was quantified from the images shown in (A); *n* = 3 independent experiments; each data point represents one retina from one mouse; ***, *P* < 0.001; one-way ANOVA. (C, D) Confluent cells from the human brain endothelial line hCMEC/D3 were treated with VEGFA, EG00229, or **12h** (30 μM) for 5 min or preincubated with EG00229 or **12h** for 15 min and then treated with VEGFA for 5 min. Cell lysates were used for immunoblotting with the indicated antibodies (C), followed by quantification of pixel intensities for P-p38 relative to total P38, as shown in (D). GAPDH was used as a loading control. Data are shown as mean fold change ± SD. Asterisks indicate significant *P*-values for phosphorylation induction after treatment; each data point represents one data point from one of 4 independent experiments; *, *P* < 0.05; **, *P* < 0.01; ***, *P* < 0.001; one-way ANOVA. (E) Diagram of the *ex-vivo* retinal permeability assay. (F) Quantification of fluorescence changes over 2 min relative to baseline (Krebs), after treatment with VEGFA (*n* = 4), EG00229 (*n* = 3), **12h** (*n* = 3), or VEGFA after **12h** pretreatment (*n* = 4). Data are shown as mean ± SD. Each data point indicates the value for one retina after one instance of adding a test substance; *, *P* < 0.05, ** *P* < 0.01,*** *P* < 0.001; one-way ANOVA. Uncropped blots for **4C** shown in Figure S2).

Second, we repeated this experiment using the human brain endothelial cell line hCMEC/D3. Cells were treated with **12h** (30 μM) or EG00229 (30 μM) for 15 min prior to VEGFA_165_ stimulation. Immunoblotting of cell lysates following the treatments showed that both **12h** and EG00229 prevented VEGFA-induced p38 phosphorylation (Figure 4C,D). Similarly, to what was observed in retinae, EG00229 induced p38 kinase phosphorylation after 5 min when added alone (Figure 4C,D) while, **12h**, on its own, did not induce p38 phosphorylation (Figure 4C,D). Thus, **12h** lacks agonist activity in both systems.

Finally, since p38 is a critical mediator of VEGFA-induced vascular leakage in the brain and retinae,^28–30^ we assessed whether **12h** inhibits VEGFA-induced vascular permeability. We measured the extravasation of fluorescent sulforhodamine B from perfused blood vessels of the mouse retina in real-time^30^ in the presence of **12h** or EG00229 (Figure 4E). As previously shown,^30^ a treatment with VEGFA_164_ (mouse equivalent of human VEGFA_165_) increased vascular permeability by ∼3-fold (Figure 4F). Preincubation of retinal explants for 15 min with **12h** (30 μM) significantly reduced VEGFA-induced dye extravasation (Figure 4F). Importantly, in agreement with the results obtained for p38 activation, **12h** alone, had no effect on vascular permeability (Figure 4F).

Taken together, these results indicate that **12h** inhibits VEGFA-induced signaling relevant for mediating pain, without activating the p38 pathway, and therefore is pharmacologically distinct from EG00229.

#### **12h** Abolishes VEGFA–Mediated Increases in Sodium Currents Recorded in Excised Rat Lumbar DRG Neurons

Given the demonstrated ability of **12h** to inhibit VEGFA_165_-induced signaling in endothelial cells (Figure 4), we next investigated whether **12h** could similarly reduce VEGFA_165_-induced effects in DRG sensory neurons. Specifically, we assessed the ability of **12h** to interfere with VEGFA_165_-induced increase in sodium currents through voltage-gated sodium channels expressed in DRG neurons.^5,12^

Typical families of Na^+^ currents from small-sized DRG neurons are shown in Figure 5A. Incubation with 1 nM concentrations of VEGFA_165_ for 30 min, resulted in nearly a 2-fold increase in both total Na^+^ currents (Figure 5A) and current density (Figure 5B,C and Table S3) compared to DMSO controls. Notably, this effect was equally reduced by inhibiting NRP1 with either EG00229 (30 μM) or **12h** (30 μM), as shown in Figure 5B,C. Neither EG00229 nor **12h** alone had any obvious effect on Na^+^ currents.

**Figure 5:**
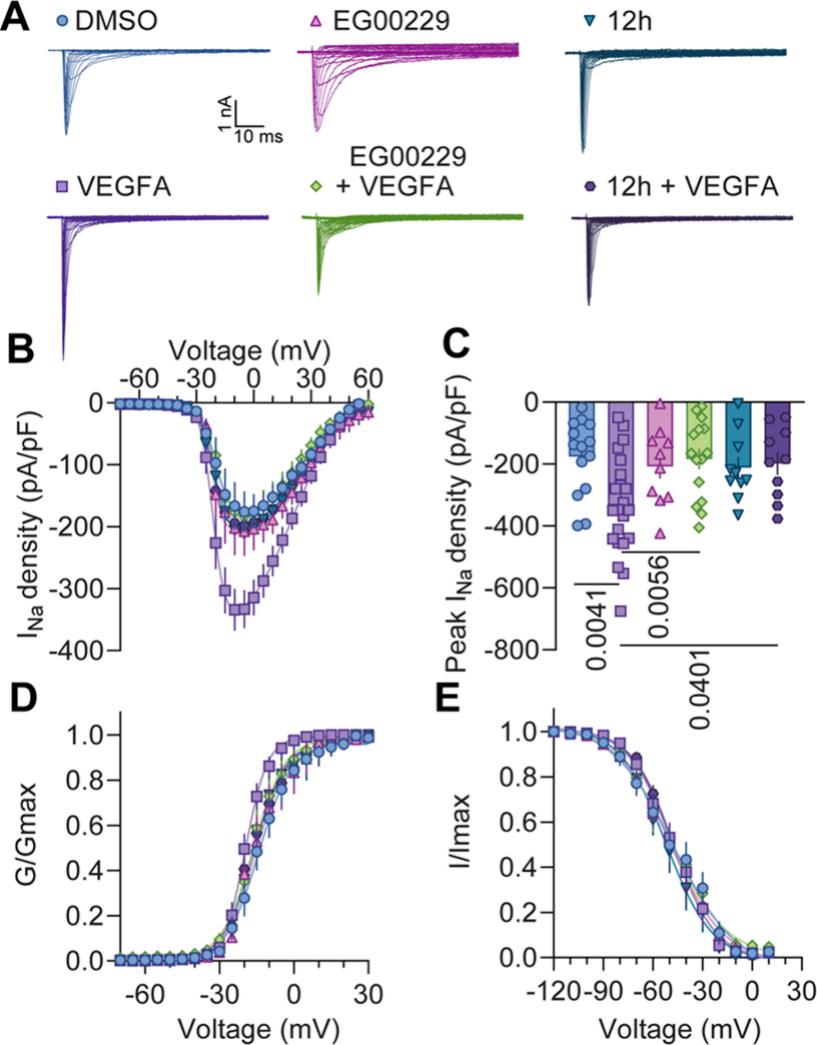
**12h** prevented the VEGFA-mediated increase in total sodium currents in DRG neurons. (A) Representative sodium current traces recorded from small–sized DRG neurons incubated for 30 min with the indicated treatments. Currents were evoked by 150 ms pulse between – 70 and +60 mV. (B) Double Boltzmann fits for current density–voltage curves. (C) Bar graph summarizing peak sodium current densities (pA/pF); *p* values as indicated; one-way ANOVA followed by Tukey’s multiple comparison test. (D, E) Boltzmann fits for voltage-dependent activation (D) and inactivation (E). Half-maximal activation and inactivation voltages (*V*_1/2_) are shown in Table 3. *N* = 10–22 cells; error bars indicate mean ± SEM (Table S3).

To determine whether voltage-dependence was also affected, we analyzed the voltage-dependent activation and inactivation of Na^+^ channels (Figure 5D,E). The half-maximal activation (*V*_1/2_) potential was significantly different when comparing the VEGFA_165_ condition with every other group (Table 3). However, no significant differences were observed in the voltage-dependence of inactivation across the conditions tested (Table 3). Overall, these functional assays suggest that the effect of **12h** can be translated into different systems demonstrating the potential of **12h** to decrease the activity of a signaling pathways involved in pain.

**Table 3:** Gating Properties of Na^+^ Currents Recorded from Rat DRG Neurons^a^

total Na^+^						
activation	**DMSO**	**VEGFA**	**EG00229** (30 μM)	**EG00229 + VEGFA**	**12h** (30 μM)	**12h + VEGFA**
*V*_1/2_	–14.099 ± 0.770 (14)^b^	–19.459 ± 0.341 (22)	–15.229 ± 0.972 (10)^b^	–16.384 ± 0.773 (15)^b^	–16.819 ± 0.840 (10)^b^	–16.420 ± 0.916 (10)^b^
Inactivation						
*V*_1/2_	–47.104 ± 2.921 (14)	–48.169 ± 1.218 (22)	–49.108 ± 2.415 (10)	–49.707 ± 2.554 (15)	–52.032 ± 2.123 (10)	–47.632 ± 1.411 (10)

a Values are means ± SEM calculated from fits of the data from the indicated number of individual cells (in parentheses) to the Boltzmann equation; *V*_1/2_ midpoint potential (mV) for voltage-dependent activation or inactivation. Data was analyzed with one-way ANOVA with Tukey post hoc test.

b *p* < 0.05 for *V*_1/2_ activation of all groups vs VEGFA.

#### **12h** Reduces VEGFA-Induced Allodynia In Vivo

Given that **12h** prevents VEGFA-induced increase in Na^+^ current density in sensory neurons we next tested whether **12h** could similarly prevent pain-like behaviors caused by VEGFA. We induced pronociception by injection of VEGFA_165_a directly into the paw, and the antinociceptive effects of **12h** were evaluated, with EG00229 used as a comparator. As expected, subcutaneous injection of VEGFA induced mechanical allodynia in male and female rats, and cold allodynia primarily in females, confirming its pronociceptive effects (Figure 6).

**Figure 6:**
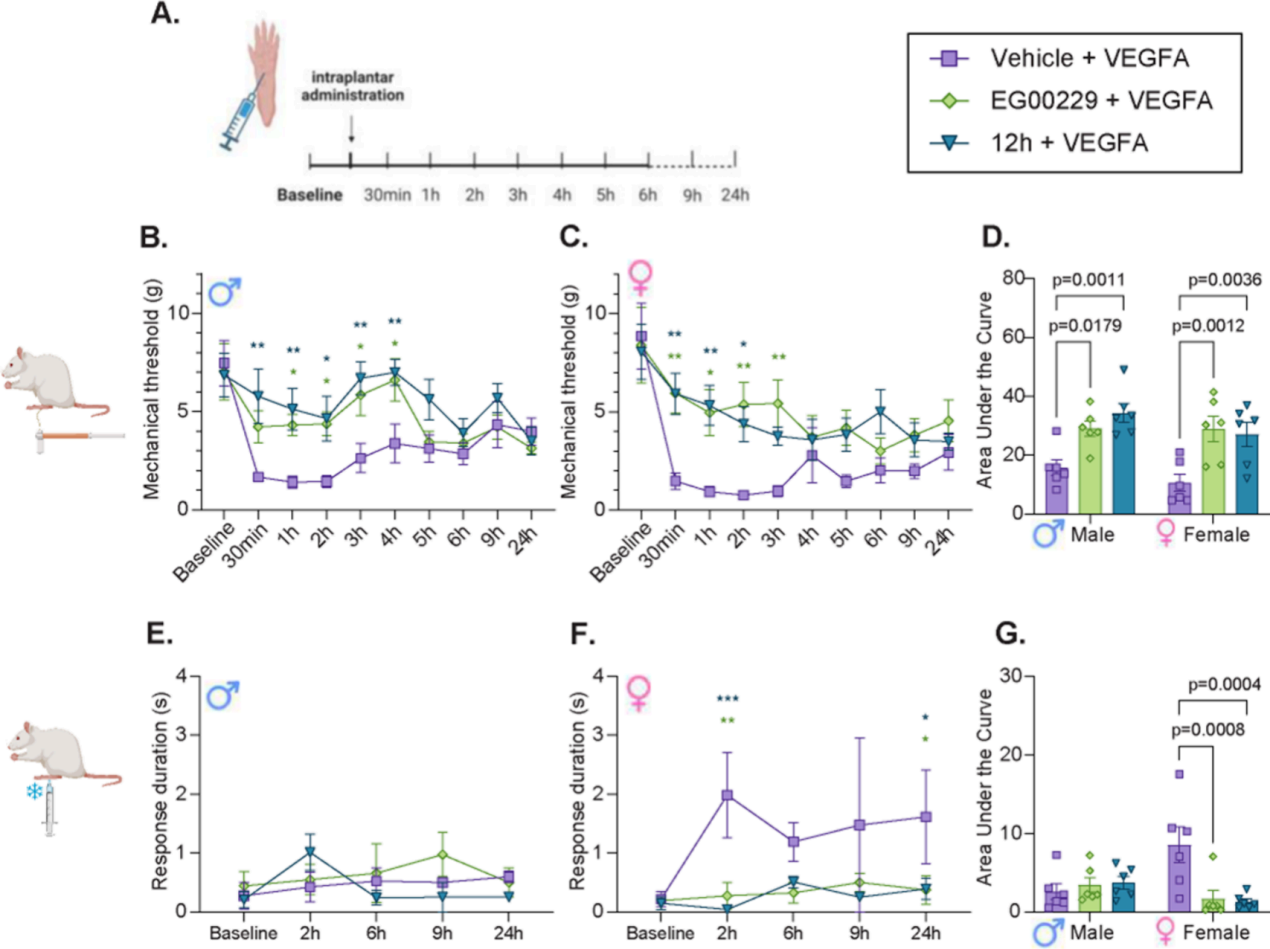
VEGFA induces a pain-like phenotype that is blocked by 12h in male and female rats. (A) Schematic of the study design and treatment conditions. Naïve male and female rats were given intraplantar injections of VEGFA_165_ (10 nM) in combination with either vehicle (PBS) or one of two NRP1 inhibitors, EG00229 or **12h** (30 μM) in a volume of 50 μL/rat. (B, C) Mechanical allodynia was assessed using paw withdrawal thresholds to mechanical stimuli (von Frey filaments, vF) in male (B) and female (C) rats. (D) Quantification of Area Under the Curve of paw withdrawal thresholds from baseline to 6 h postadministration. (E–G) Cold allodynia was assessed using the Acetone Drop Test (ADT), by recording the response duration in male (E) and female (F) rats. (G) Quantification of Area Under the Curve of the response duration to ADT from baseline to 6 h after injection. Error-bars represent mean ± SEM, and sex is indicated by ♂ (male) and ♀ (female). Time-course data were analyzed using two-way repeated measures ANOVA, with Tukey’s post hoc test (*p < 0.05, **p < 0.01, ***p < 0.001). AUC data were analyzed using two-way ANOVA, with Dunnett’s post hoc test, suggesting differences from the sex-specific vehicle treatment group. *n* = 6–7. For full statistical analyses, see Table S3.

When NRP1 inhibitors were coinjected with VEGFA (30 μM) as previously reported,^5^ the development of mechanical allodynia was blunted in both males (Figure 6B) and females (Figure 6C). AUC analysis for each animal during the first 6 h of the experiment, and following statistical analysis (Two-way ANOVA, Supplementary Table S3) showed significant effects of treatment (*p* < 0.0001), and no sex-differences. Dunnett’s posthoc test confirmed that both inhibitors were effective at alleviating the VEGFA-induced mechanical allodynia (Figure 6D).

Hypothesizing that the two compounds at this concentration might have reached the maximum possible effect and thus masked potential small differences in potency, we also assessed a lower concentration, (10 μM). At this dose, we found that only males (Figure S3) showed statistically significant effects of treatment, and statistical post hoc tests indicated that only EG00229 – but not **12h** — produced significant antinociceptive effects (Figure S3). No significant effects of administration of either of the inhibitors were observed in females at this dose (Figure S3).

Cold allodynia was detected by an increase in response duration following the application of an acetone drop. We found that male rats showed minimal signs of VEGFA-induced cold allodynia, and as expected there was no effect of the inhibitors in this sex (Figure 6E). In contrast, VEGFA induced an increased cold-like response time in females, which was significantly reduced by both inhibitors of NRP1 at 30 μM concentrations (Figure 6F). These findings were also confirmed by AUC analysis that detected significant effects of treatment-group (*P* = 0.026), and a significant interaction between the “sex” and “treatment-group” factors (*P* = 0.0028), indicating sex-specific differences in cold sensitivity (Figure 6G; Table S3). When testing the lower dose (10 μM), we found no modifying effects of any of the NRP1 inhibitors on the cold allodynia outcome in any sex (Figure S3). For full statistical analysis, see Table S4.

#### VEGFA Increased Aversion to Mechanical Stimuli Was Reduced by Intraplantar Injections of **12h**

In addition to evaluating sensory thresholds to evoked stimuli, we wanted to assess if VEGFA induces aversion to a medium force mechanical stimulation, and whether NRP1 inhibition could prevent this. We used a two-chamber conditioned place aversion (CPA) test.^42^ Animals were injected intraplantarly with either saline or VEGFA_165_ and 1 h later tested in the CPA paradigm. The tests consisted of four 10 min sessions; schematic of the study design is shown in Figure 7A. During preconditioning, rats were given free access to both chambers, each paired with a scent (such as strawberry or spearmint). During conditioning, the rat was confined to one chamber at a time, which was paired with repeated mechanical stimulation (10 g vF-filament) every 30 s or no stimulation (NS). During the testing phase, the rats were once more given free access to both chambers, and aversion was measured by the reduced time spent in the chamber conditioned with stimulation. As shown by baseline measures in Figure 6, 10 g stimulation is typically above the threshold in most test-subjects, meaning that it often induces a withdrawal threshold even under naïve conditions. We hypothesized that the stimulation would not cause aversion under naïve circumstances, but that a prior intraplantar injection of VEGFA would make the stimulation aversive, as seen with other injury-models previously tested (Hestehave and co-workers).^43,44^ First, we therefore conducted a pilot experiment to evaluate whether mechanical stimulation would induce CPA in VEGFA-injected rats. (Figure S4). Naïve rats injected with PBS-vehicle spent an equal amount of time in both chambers both during preconditioning and testing (Figure S4), suggesting that the stimuli were not considered aversive. In contrast, the animals injected with VEGFA spent significantly less time in the vF-conditioned chamber during the test (Figure S4). To confirm the difference, we quantified the CPA-score by calculating the difference in time spent in the vF-chamber between test-phase and preconditioning phase, and revealed a significantly higher aversion to the 10 g stimulation in VEGFA-treated animals (Figure S4).

**Figure 7:**
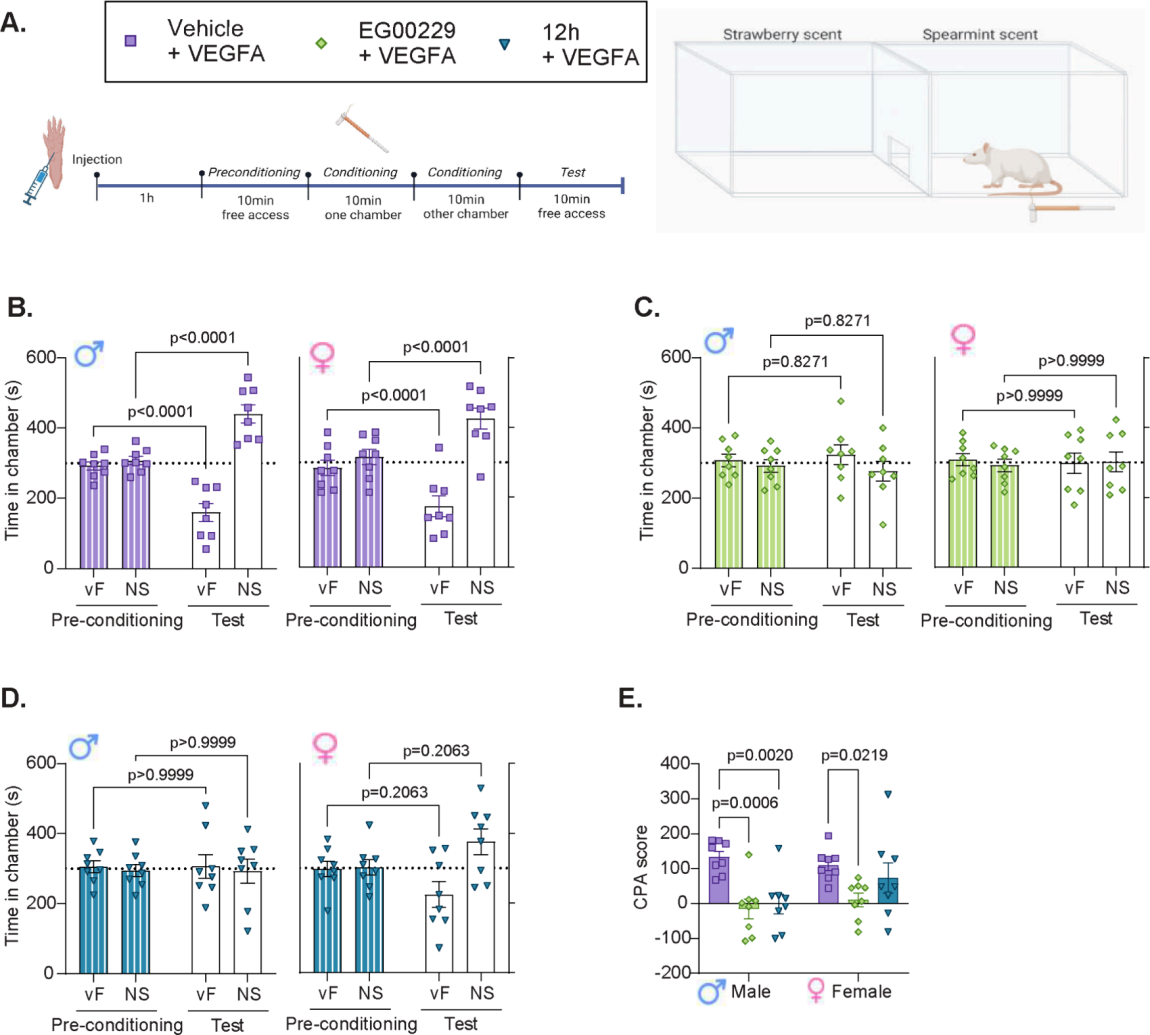
NRP1 inhibitor **12h** blocks VEGFA-induced increase in aversion to mechanical stimuli in rats. VEGFA (10 nM) was administered interplantarly to naïve male and female rats, in combination with either vehicle (PBS) or the NRP1 inhibitors, EG00229 or **12h** (50 μL of 30 μM per rat). (A) Schematic of the study design. One hour after injection, rats were exposed to the two-chamber CPA-test, including four consecutive 10 min sessions of: preconditioning, conditioning to each chamber, and testing. The conditioning phase included one chamber conditioned with stimulation using a 10 g vF-filament every 30 s, while the other chamber received no stimulation (NS). (B) Animals treated with VEGFA/vehicle showed increased aversion to the vF-conditioned chamber during testing of both male (♂) and female (♀) rats. (C) NRP1 inhibitor EG00229 prevented the stimulus-aversion from VEGFA in both sexes. (D) NRP1 inhibitor **12h** prevented significant stimulus-aversion from VEGFA in both sexes. (E) NRP1 inhibitors prevented the aversive effects from VEGFA as demonstrated by decreased CPA-scores when compared with vehicle. CPA score = time in vF-chamber during preconditioning – time in vF-chamber during testing phase. *n* = 8. *P*-values as suggested by appropriate post hoc test. For full statistical analysis, see Table S3.

Next, we examined weather coinjection of NRP1 inhibitors with VEGFA, could reduce the observed aversion. Again, male and female rats injected with VEGFA alone, spent significantly less time in the vF-conditioned chamber during the test (Figure 7B). However, when EG00229 (Figure 7C) or **12h** (Figure 7D) were coinjected with VEGFA, the aversion was prevented.

CPA-score analysis confirmed that both inhibitors significantly decreased VEGFA-induced aversion in males, while only EG00229 showed a significant effect in females (Figure 7E). The outcome of the **12h** injection resulted in more variable results in females, and although there was no significantly increased aversion (Figure 7D), the CPA score did not show a statistically significant improvement compared to the VEGFA/vehicle group (Figure 7E). When lower doses (10 μM) of the inhibitors were tested in the CPA-paradigm, we found that neither compound had an effect in females. Intriguingly, under these conditions **12h** showed superior efficacy in males - while EG00229 had no effect, **12h** showed significant reduction in VEGFA-induced aversion (Figure S5, Table S4).

The landscape of VEGFA isoforms, their receptors, and pain signaling is complex and not yet fully understood.^20^ NRP1 mRNA has been found in various neuronal structures including the olfactory bulb, hippocampus, cerebellum, cortex, motoneurons in the spinal cord and DRGs.^45–49^ Expression of NRP1 in DRG neurons is upregulated following nerve injury,^49,50^ and after both peripheral and central lesions in DRG, NRP1 mRNA expression also increases in the spinal cord’s superficial laminae,^8^ implicating NRP1 in mediating pain response.

In this work, we focused on the role of the NRP1/VEGFR signaling axis and the effect of NRP1 inhibition in VEGF-induced pain models. We are build upon our previous studies which showed that in the sensory system, CRISPR/Cas9-mediated knockdown of NRP1 prevents VEGFA-induced enhancement of Ca_V_2.2 and Na_V_1.7 currents, similarly impacting spinal cord neurotransmission and pain-like behaviors.^51^ Comparable results were observed when NRP1 is inhibited with EG00229,^5^ underscoring the potential for developing new therapeutic agents targeting this pathway. Recently, we demonstrated that NRP1 inhibitor EG00229 effectively suppresses nerve growth factor (NGF)-evoked sensitization of mouse and human nociceptors, as well as mechanical allodynia and thermal hyperalgesia in mice.^49^ Despite improved useful analgesic properties, further optimization of NRP1 ligands is needed to increase in vivo potency against VEGFA-induced pain-like behavior.

We now report on a new compound, **12h**, which exhibits notable differences in its inhibitory and pharmacokinetic profile compared to EG00229. Our in vitro experiments demonstrated that **12h** is as effective as EG00229 in inhibiting sodium currents in sensory neurons. In vivo experiments comparing the two compounds in VEGFA-induced mechanical and cold allodynia in rats revealed sex-based differences in their effects. At 30 μM, both EG00229 and **12h** produced comparable antinociceptive effects in males and females. However, at lower doses (10 μM) only EG00229 showed measurable effects, and only in males.

Intriguingly, further assessment of pain-like behavior using the Conditioned Place Aversion paradigm^43,44,52,53^ revealed concentration- and sex-dependent differences in the inhibitory profiles of the two compounds. In these experiments we first established that VEGFA injection into the paw caused an increased aversion to mechanical stimuli. This aversive quality of the stimuli was blocked by 30 μM of either of NRP1 inhibitors in males, with EG00229 showing superior activity compared to **12h** in females. At lower concentrations of inhibitors, neither compound affected females but **12h** retained potency in preventing aversion in male rats. These findings suggest that although **12h** at lower concentrations did not prevent mechanical sensitivity, it was more effective than EG00229 in mitigating the aversive quality of the stimuli. To benchmark against our previous studies using EG00229,^12^ in all experiments reported here, we employed local administration in rats, which mimics phenotypes observed in traditional pain models while conserving compound usage.

The observed in vivo activity of NRP1 inhibitors correlated with their effects on p38 (MAPK14), a key regulatory kinase in pain transmission. Having established that EG00229 blocks VEGFA signaling and p38-dependent vascular permeability, we demonstrated that **12h** exhibits comparable activity. However, we also found that despite inhibiting VEGFA signaling via NRP1, EG00229 on its own also induces unwanted p38 activation and vascular permeability in human brain endothelial cells. In contrast, **12h** does not activate undesirable p38 signaling and therefore appears to be the superior compound for blocking VEGF signaling. As the p38 pathway activation has been implicated in several pain models,^31,42,54^ future studies should compare **12h** and EG00229 with respect to p38 activation in VEGFA-induced pain models. If NRP1 is to be pursued as a pain target, then elimination of unwanted physiological effects such as increased vascular permeability are important to maximize the potential clinical benefit.

The mechanistic basis for the differing effectiveness of **12h** compared to EG00229 in cellular and animal studies may originate in its structural and biophysical properties. X-ray crystallography of the ligand/NRP1 complex revealed improved hydrogen-bond stabilization and potential out-of-pocket interactions, consistent with the enhanced binding affinity and altered kinetics observed by SPR. These subtle structural differences appear to shift the pharmacology from partial agonism (EG00229) to purely inhibitory type of **12h**, as evidenced by the loss of p38 activation in the vasculature.

We acknowledge that our study has several limitations. First, we assessed the effects of EG00229 and **12h** on VEGFA-induced pain-related outcomes,^5^ but did not address the potential off-target effects. Although EG00229 has previously shown selectivity over the closely related NRP2,^41^ we did not evaluate **12h** interaction with NRP2. Future research should investigate the effects of systemic administration in traditional pain models and further explore sex-dependent differences in VEGFA and NRP1 inhibition in pain conditions.

## Conclusions

Considering the in vitro NRP1-binding activity, *ex vivo* inhibitory potency and pharmacokinetic profiles, coupled with the in *vivo* efficacy, **12h** emerges as the superior compound to EG00229 for blocking VEGFA-mediated signaling and the downstream pain-related effects. **12h** represents a valuable tool for further investigation of VEGFA-induced pain-like behavior and for the development of molecules with the therapeutic potential. Notably, the pharmacokinetic advantages of small molecules over antibodies and soluble receptors will likely be important to develop effective analgesic drugs, especially considering the ongoing opioid crisis.^55,56^

## Experimental Section

### Animal Ethics Statement

Animal studies ethics and approvals.

Pharmacokinetic studies: all in vivo study protocols, husbandry and anesthesia followed guidelines of United Kingdom Home Office Scientific Procedures Act (1986).

Permeability study: Animal work was performed following UK Home Office Animals in Science Procedures e-Licensing (ASpeL) and institutional Animal Welfare and Ethical Review Body (AWERB) guidelines.

Pain studies: the NYU Grossman School of Medicine’s Institutional Animal Care and Use committee (Approval numbers: PROTO202100104).

### Chemistry

All materials were obtained from commercial suppliers and used without further purification unless otherwise noted. Anhydrous solvents were either obtained from Aldrich or Fisher Scientific and used directly. All reactions involving air- or moisture-sensitive reagents were performed under a nitrogen atmosphere. Routine analytical thin layer chromatography was performed on precoated plates (Alugram, SILG/UV254). Reaction analyses and purity were determined by reverse-phase LC-MS using an analytical C18 column (Phenomenex Luna C18 (2) 50 × 4.6 mm, 5 μm for 4.5 and 13 min methods), using a diode array detector and an A:B gradient starting from 95% A: 5% B at a flow rate of 2.25 or 1.5 mL/min, where eluent A was 0.1% formic acid/H_2_O and eluent B was 0.1% formic acid/MeOH or eluent A was 10 mM NH_4_HCO_3_ (aq.) and eluent B: MeOH. Silica gel chromatography was performed with prepacked silica gel Biotage SNAP (KP-Sil) cartridges. Ion exchange chromatography was performed using Isolute Flash SCX-2 cartridges. Reverse-phase preparative HPLC was carried out on a Waters ZQ instrument using mass-directed purification on a preparative C18 column (Phenomenex Luna C18 (2), 100 × 21.2 mm, 5 μm). Depending upon the retention time and the degree of separation of the desired compound from any impurities, an A:B gradient was employed starting from high %A/low %B at a flow rate of 20 mL/min. The following combinations of A and B were typically used: A = H_2_O + 0.1% formic acid: B = MeOH (or ACN) + 0.1% formic acid or A = 10 mM NH_4_HCO_3_ (aq): B = methanol. ^1^H and ^13^C spectra were measured with a Bruker DRZ 400 MHz spectrometer. All observed protons are reported as parts per million (ppm) and are aligned to the residual solvent peak e.g., for DMSO-*d*_6_ at δ_H_ 2.50 and δ_C_ 39.5 and for CDCl_3_ at δ_H_ 7.26. Data are reported as follows: chemical shift, multiplicity (s = singlet, d = doublet, t = triplet, br = broad, m = multiplet), coupling constants (*J*) recorded in Hz, and number of protons. Low-resolution mass spectrometry data were determined on Waters ZQ4000 single quadruple or Micromass Ultima triple quadruple mass spectrometers. High-resolution mass spectrometry was determined using Positive Ion Electrospray on the Orbitrap.

Purity statement: All compounds tested (bioassays) were determined to be at least 95% pure by LC-MS unless otherwise stated.

### Compound Synthesis and Characterization

#### 6-Bromoquinoline-8-sulfonyl Chloride (**5**)

6-Bromoquinoline (10.0 g, 48.3 mmol) was added portion-wise to chlorosulfonic acid (100 mL, 0.5 M) at rt. After completion of the addition, the reaction mixture was heated at 160 °C for 18 h. The reaction mixture was cooled to rt, then poured dropwise into an ice-cold water (1000 mL) and stirred for 30 min. The precipitated solid was collected by filtration, washed with excess water and dried. The crude compound was purified by column chromatography (100–200 mesh silica-gel, eluted with CHCl_3_) to afford compound **2** (2.0 g, 13%) as an off-white solid.

LCMS: Rt 2.76 min, (ESI^+^) *m*/*z* 306.0, 308.0, 310.0 [M + H]^+^, Purity 94%.

^1^H NMR (400 MHz, CDCl_3_) δ 9.24 (dd, *J* = 4.1, 1.9 Hz, 1H), 8.62 (d, *J* = 2.2 Hz, 1H), 8.38 (d, *J* = 2.3 Hz, 1H), 8.24 (d, *J* = 8.0 Hz, 1H), 7.66 (m, 1H).

#### Methyl 3-((6-Bromoquinoline)-8-sulfonamido)­thiophene-2-carboxylate (**6**)

Anhydrous pyridine (30 mL, 0.6 M) was added dropwise to 6-bromoquinoline-8-sulfonyl chloride **5** (5.0 g, 16.4 mmol) at 0 °C under N_2_ atmosphere over a period of 30 min. A solution of methyl-3-aminothiophene-2-carboxylate (2.6 g, 16.4 mmol) in anhydrou*s* pyridine (30 mL, 0.6 M) was added dropwise to the above reaction mixture over a period of 50 min and stirred at rt for 20 h. The reaction mixture was then poured into an ice-cold water (350 mL) and the resulting precipitated solid was collected by filtration and dried to give compound **6** (5.5 g, 79%) as a pale orange solid.

LCMS: Rt 3.59 min, (ESI^–^) *m*/*z* 425.0, 427.0, [M-H]^−^, Purity 94%.

^1^H NMR (400 MHz, CDCl_3_): δ 10.70 (s, 1H), 9.10 (dd, *J* = 4.2, 1.7 Hz, 1H), 8.53 (d, *J* = 2.2 Hz, 1H), 8.24–8.10 (m, 2H), 7.59–7.48 (m, 2H), 7.33 (d, *J* = 5.5 Hz, 1H), 3.82 (s, 3H).

#### 3-((6-Bromoquinoline)-8-sulfonamido)­thiophene-2-carboxylic Acid (**7**)

To a solution of compound **6** (2.2 g, 5.2 mmol) in THF (22 mL, 0.2 M), 2 M (aq.) LiOH solution (22 mL, 0.2 M) was added and heated at 140 °C for 3 h in a sealed tube. Then the reaction mixture was cooled to rt, diluted with water and acidified (pH ∼ 4) with 1 N HCl at 0 °C and stirred for 15 min. The resulting precipitated solid was collected by filtration, washed with water and dried to give compound **7** (2.0 g, 94%) as off white solid.

LCMS: Rt 2.32 min, (ESI^–^) *m*/*z* 411.0, 413.0, [M-H]^−^, Purity 94%.

1H NMR (400 MHz, DMSO-*d*_6_): δ 13.64 (s, 1H), 10.75 (s, 1H), 9.01 (dd, *J* = 4.2, 1.7 Hz, 1H), 8.67 (d, *J* = 2.2 Hz, 1H), 8.55–8.43 (m, 2H), 7.82–7.69 (m, 2H), 7.35 (d, *J* = 5.5 Hz, 1H).

#### Methyl N2-(3-((6-Bromoquinoline)-8-sulfonamido)­thiophene-2-carbonyl)-Nw-((2,2,4,6,7-pentamethyl-2,3-dihydrobenzofuran-5-yl)­sulfonyl)-l-argininate (**8**)

To a solution of compound **6** (5.0 g, 12.1 mmol) in CH_2_Cl_2_ (50 mL, 0.2 M), PyBrOP (8.5 g, 18.2 mmol) was added at 10 °C, followed by DIPEA (15 mL, 84.9 mmol) and l-Arg­(Pbf)­OMe (5.2 g, 10.9 mmol) and allowed to stir at rt for 16 h. The reaction mixture was diluted with CH_2_Cl_2_ (200 mL), washed successively with cold water (50 mL), 1 M (aq.) HCl (2 × 50 mL), brine solution (50 mL), then dried over anhydrous Na_2_SO_4_ and concentrated. The crude compound was purified by column chromatography (100–200 mesh silica-gel, eluted with 3% MeOH/CH_2_Cl_2_) to afford compound **8** (5.5 g, 54%) as an off-white solid.

LCMS: Rt 3.78 min, (ESI^–^) *m*/*z* 833.0, 835.0, [M-H]^−^, Purity 96%.

1H NMR (400 MHz, DMSO-*d*_6_): δ 11.38 (s, 1H), 8.91 (d, *J* = 3.9 Hz, 1H), 8.62 (s, 1H), 8.41 (m, 3H), 7.64 (m, 2H), 7.28 (d, *J* = 5.5 Hz, 1H), 6.90 (s, 1H), 6.72 (br s, 1H), 6.43 (br s, 1H), 4.35 (m, 1H), 3.66 (s, 3H), 3.03 (d, *J* = 7.2 Hz, 2H), 2.90 (s, 2H), 2.49 (s, 3H), 2.41 (s, 3H), 1.95 (s, 3H), 1.77–1.74 (m, 1H), 1.64 (m, 1H), 1.38 (m, 8H).

#### N2-(3-((6-Bromoquinoline)-8-sulfonamido)­thiophene-2-carbonyl)-Nw-((2,2,4,6,7-pentamethyl-2,3-dihydrobenzofuran-5-yl)­sulfonyl)-l-arginine (**9**)

To a solution of compound **8** (2.2 g, 5.2 mmol) in THF (32 mL, 0.2 M) and water (13 mL, 0.4 M) was added LiOH.H_2_O (485 mg, 21.1 mmol) at 0 °C and then stirred at rt for 4 h. Then the reaction mixture was diluted with water (100 mL), cooled to 0 °C, acidified (pH ∼ 4) with 1 M HCl, extracted with EtOAc (2 × 150 mL). The combined organic layer was successively washed with H_2_O (50 mL) and brine solution (50 mL), dried over anhydrous Na_2_SO_4_ and concentrated to afford compound **9** (2.0 g, 93%) as an off-white solid.

LCMS: Rt 3.12 min, (ESI^+^) *m*/*z* 821.5, 823.5, 824.6 [M + H]^+^, Purity 94%.

1H NMR (400 MHz, DMSO-*d*_6_) δ 12.70–12.62 (m, 1H), 11.41 (s, 1H), 8.98–8.82 (m, 1H), 8.61 (d, *J* = 2.5 Hz, 1H), 8.41 (d, *J* = 5.6 Hz, 2H), 8.26 (d, *J* = 7.5 Hz, 1H), 7.62 (m, 2H), 7.26 (d, *J* = 5.4 Hz, 1H), 6.63 (br s, 1H), 6.56–6.27 (br s, 2H), 4.36–4.22 (m, 1H), 3.01 (m, 2H), 2.91 (s, 2H), 2.47 (s, 3H), 2.41 (s, 3H), 1.95 (s, 3H), 1.86–1.69 (m, 1H), 1.62 (br s, 1H), 1.38 (s, 8H).

#### N2-(3-((6-(3-Formylphenyl)­quinoline)-8-sulfonamido)­thiophene-2-carbonyl)-Nw-((2,2,4,6,7-pentamethyl-2,3-dihydrobenzofuran-5-yl)­sulfonyl)-l-arginine (**10a**)

A mixture of Compound **9** (150 mg, 0.2 mmol), 3-formylphenylboronic acid (58 mg, 0.4 mmol), K_3_PO_4_ (163 mg, 0.8 mmol) in THF-H_2_O (5 mL, 0.04 M, 1:0.1) was degassed with argon for 30 min in a thick-well borosilicate glass vial. Pd­(PPh_3_)_4_ (11 mg, 0.1 mmol) was then added, and the reaction mixture was degassed again for 15 min and irradiated in the *M*_W_ at 90 °C for 30 min. The reaction mixture was then diluted with H_2_O (15 mL), cooled to 0 °C, acidified with 2 M HCl (30 mL) and extracted with EtOAc (2 × 50 mL). The combined organic layer was washed successively with water (20 mL) and brine solution (20 mL), dried over anhydrous Na_2_SO_4_ and concentrated. The crude compound was purified by preparative HPLC to afford compound **10a** (60 mg, 39%) as an off-white solid.

LCMS: Rt 1.36 min, (ESI^–^) *m*/*z* 845.3, [M – H]^−^, Purity 99%.

^1^H NMR (400 MHz, DMSO-*d*_6_) δ 12.75 (s, 1H), 11.44 (br s, 1H), 10.16 (s, 1H), 8.88 (s, 1H), 8.71 (s, 2H), 8.51- 8.39 (m, 2H), 8.21 (s, 1H), 8.01 (d, *J* = 7.6 Hz, 1H), 7.80 (m, 1H), 7.61–7.5 (m, 2H), 7.29 (m, 2H), 7.00–6.67 (m, 2H), 6.40 (s, 1H), 4.27 (m, 1H), 3.08 (d, *J* = 16.8 Hz, 2H), 2.93 (s, 2H), 2.48 (s, 3H), 2.41 (s, 3H), 1.97 (s, 3H), 1.78 (m, 1H), 1.67 (m, 1H), 1.38 (s, 8H).

#### N2-(3-((6-(4-Formylphenyl)­quinoline)-8-sulfonamido)­thiophene-2-carbonyl)-Nw-((2,2,4,6,7-pentamethyl-2,3-dihydrobenzofuran-5-yl)­sulfonyl)-l-arginine (**10b**)

The compound was prepared according to the same procedure as Compound **10a** starting from Compound **9** (300 mg, 0.4 mmol) and 4-formylphenylboronic acid. The crude compound was purified by preparative HPLC to afford compound **10b** (210 mg, 68%) as off-white solid.

LCMS: Rt 1.32 min, (ESI^–^) *m*/*z* 845.2, [M-H]^−^, Purity 95%.

^1^H NMR (400 MHz, DMSO-*d*_6_) δ 10.10 (s, 1H), 8.96 (br s, 1H), 8.68 (d, *J* = 2.4 Hz, 1H), 8.48 (dd, *J* = 9.0, 5.2 Hz, 2H), 8.32 (s, 1H), 8.07 (q, 4H), 7.58 (s, 2H), 7.22 (d, *J* = 5.5 Hz, 1H), 7.14 (d, *J* = 5.6 Hz, 1H), 6.99 (br s, 2H), 6.41 (s, 1H), 4.04 (m, 1H), 3.06 (m, 2H), 2.95 (m, 2H), 2.43 (s, 3H), 2.41 (s, 3H), 2.00 (s, 3H), 1.75–1.52 (m, 4H), 1.40 (s, 6H).

#### (3-((6-(3-(((1-Methyl-1H-imidazol-5-yl)­methyl)­amino)­phenyl)­quinoline)-8-sulfonamido)­thiophene-2-carbonyl)-l-arginine (**11a**)

To a solution of compound **10a** (85 mg, 0.1 mmol) in THF-MeOH (4 mL, 0.02 M, 1:1) were added (1-methyl-1H-imidazol-5-yl)­methylamine (18 mg, 0.2 mmol) and AcOH (few drops) at 0 °C and the reaction mixture was stirred at rt for 2 h. NaCNBH_3_ (12 mg, 0.2 mmol) was then added, and the reaction mixture was stirred for further 2 h. The reaction mixture was quenched with ice water (5 mL) and concentrated. The crude compound was purified by preparative HPLC to afford compound **11a** (40 mg, 42%) as an off-white solid.

LCMS: Rt 2.13 min, (ESI^–^) *m*/*z* 940.4, [M-H]^−^, Purity 99%.

^1^H NMR (400 MHz, DMSO-*d*_6_) δ 8.82 (s, 1H), 8.66 (s, 1H), 8.45 (d, *J* = 9.2 Hz, 2H), 7.83 (s, 1H), 7.73 (d, *J* = 7.4 Hz, 2H), 7.63–7.42 (m, 6H), 7.33 (s, 1H), 7.24 (d, *J* = 5.5 Hz, 1H), 6.89 (s, 2H), 6.40 (s, 2H), 4.19 (m, 1H), 3.94–3.84 (m, 4H), 3.62 (t, *J* = 6.0 Hz, 3H), 3.17–3.11 (m, 2H), 2.94 (s, 2H), 2.43 (s, 3H), 2.41 (s, 3H), 1.99 (s, 3H), 1.81–1.57 (m, 4H), 1.39 (s, 8H).

#### N2-(3-((6-(4-((Methylamino)­methyl)­phenyl)­quinoline)-8-sulfonamido)­thiophene-2-carbonyl)-Nw-((2,2,4,6,7-pentamethyl-2,3-dihydrobenzofuran-5-yl)­sulfonyl)-l-arginine (**11b**)

The compound was prepared according to the same procedure as Compound **11a** starting from Compound **10b** (100 mg, 0.1 mmol) and 2 M MeNH_2_ in THF (0.18 mL, 0.3 mmol). The crude compound was purified by preparative HPLC to afford compound **11b** (40 mg, 36%) as off-white solid.

LCMS: Rt 2.97 min, (ESI^–^) *m*/*z* 860.3, [M-H]^−^, Purity 97%.

^1^H NMR (400 MHz, DMSO-*d*_6_) δ 9.93 (br s, 1H), 8.83 (s, 2H), 8.61 (s, 1H), 8.46–8.35 (m, 2H), 7.82 (d, *J* = 7.9 Hz, 2H), 7.61–7.49 (m, 4H), 7.20 (s, 2H), 6.94 (m, 1H), 6.38 (br s, 2H), 4.06 (m, 3H), 3.15 (m, 2H), 2.94 (s, 2H), 2.50 (s, 3H), 2.41 (s, 3H), 2.40 (s, 3H), 2.00 (s, 3H), 1.75–1.65 (m, 2H), 1.40 (s, 8H).

#### N2-(3-((6-(4-((((1-Methyl-1H-imidazol-5-yl)­methyl)­amino)­methyl)­phenyl)­quinoline)-8-sulfonamido)­thiophene-2-carbonyl)-Nw-((2,2,4,6,7-pentamethyl-2,3-dihydrobenzofuran-5-yl)­sulfonyl)-l-arginine (**11c**)

The compound was prepared according to the same procedure as Compound **11a** starting from Compound **10b** (120 mg, 0.1 mmol) and (1-methyl-1H-imidazol-5-yl)­methylamine (24 mg, 0.2 mmol). The crude compound was purified by preparative HPLC to afford compound **11c** (65 mg, 49%) as off-white solid.

LCMS: Rt 2.09 min, (ESI^–^) *m*/*z* 940.3, [M-H]^−^, Purity 96%.

^1^H NMR (400 MHz, DMSO-*d*_6_) δ 8.84 (s, 1H), 8.63 (d, *J* = 2.2 Hz, 1H), 8.51–8.41 (m, 2H), 7.82 (d, *J* = 7.7 Hz, 2H), 7.65 (s, 1H), 7.57 (d, *J* = 7.9 Hz, 3H), 7.39 (s, 1H), 7.26 (d, *J* = 5.5 Hz, 1H), 6.92–6.84 (m, 3H), 6.40 (br s, 2H), 4.22 (m, 1H), 3.92 (s, 2H), 3.86 (s, 2H), 3.66 (s, 3H), 3.13–3.07 (m, 2H), 2.93 (s, 2H), 2.45 (s, 3H), 2.42 (s, 3H), 1.98 (s, 3H), 1.79–1.64 (m, 3H), 1.52 (m, 2H), 1.39 (s, 8H).

#### N2-(3-((6-(3-(Aminomethyl)­phenyl)­quinoline)-8-sulfonamido)­thiophene-2-carbonyl)-Nw-((2,2,4,6,7-pentamethyl-2,3-dihydrobenzofuran-5-yl)­sulfonyl)-l-arginine (**11d**)

To a solution of compound **9** (200 mg, 0.2 mmol) and (3-(aminomethyl)­phenyl)­boronic acid (76 mg, 0.5 mmol) in THF-H_2_O (0.2 M, 10:1) was added K_2_CO_3_ (138 mg, 1.0 mmol) and the reaction mixture was degassed with argon for 15 min in a thick-well borosilicate glass vial. Pd­(PPh_3_)_4_ (0.1 mmol, 10 mol %) was then added and the reaction mixture was degassed again for 15 min and irradiated in the MW at 130 °C for 30 min. Then the reaction mixture was cooled to rt and concentrated. The crude compound was purified by preparative HPLC to afford the corresponding coupled product and used directly in the Suzuki step.

LCMS: Rt 2.54 min, (ESI^–^) *m*/*z* 846.3, [M – H]^−^, Purity 99%.

^1^H NMR (400 MHz, DMSO-*d*_6_) δ 9.95 (br s, 2H), 8.80 (s, 1H), 8.68 (d, *J* = 2.3 Hz, 1H), 8.44–8.34 (m, 3H), 7.92 (s, 1H), 7.82 (d, *J* = 7.8 Hz, 1H), 7.55 (m, 3H), 7.20 (d, *J* = 5.4 Hz, 2H), 6.90 (s, 2H), 6.38 (s, 2H), 4.15 (m, 3H), 3.11 (m, 2H), 2.93 (m, 2H), 2.47 (s, 3H), 2.40 (s, 3H), 1.98 (s, 3H), 1.75–1.66 (m, 4H), 1.38 (s, 6H).

#### N2-(3-((6-(4-(Aminomethyl)­phenyl)­quinoline)-8-sulfonamido)­thiophene-2-carbonyl)-Nw-((2,2,4,6,7-pentamethyl-2,3-dihydrobenzofuran-5-yl)­sulfonyl)-l-arginine (**11e**)

To a solution of compound **9** (200 mg, 0.2 mmol) and (4-(aminomethyl)­phenyl)­boronic acid (76 mg, 0.5 mmol) in THF-H_2_O (0.2 M, 10:1) was added K_2_CO_3_ (138 mg, 1.0 mmol) and the reaction mixture was degassed with argon for 15 min in a thick-well borosilicate glass vial. Pd­(PPh_3_)_4_ (0.1 mmol, 10 mol %) was then added and the reaction mixture was degassed again for 15 min and irradiated in the *M*_W_ at 130 °C for 30 min. Then the reaction mixture was cooled to rt and concentrated. The crude compound was purified by preparative HPLC to afford the corresponding coupled product and used directly in the Suzuki step.

LCMS: Rt 2.50 min, (ESI^–^) *m*/*z* 846.3, [M – H]^−^, Purity 97%.

^1^H NMR (400 MHz, DMSO-*d*_6_) δ 10.01 (s, 1H), 8.81 (s, 1H), 8.61 (s, 2H), 8.46–8.35 (m, 3H), 7.84 (d, *J* = 7.9 Hz, 2H), 7.62 (d, *J* = 7.9 Hz, 2H), 7.53 (m, 1H), 7.22 (s, 2H), 6.92 (s, 1H), 6.38 (s, 2H), 4.11 (m, 3H), 3.14 (m, 2H), 2.94 (m, 2H), 2.46 (s, 3H), 2.40 (s, 3H), 2.00 (s, 3H), 1.77–1.69 (m, 4H), 1.40 (s, 6H).

#### N2-(3-((6-(2-Aminopyridin-4-yl)­quinoline)-8-sulfonamido)­thiophene-2-carbonyl)-Nw-((2,2,4,6,7-pentamethyl-2,3-dihydrobenzofuran-5-yl)­sulfonyl)-l-arginine (**11f**)

To a solution of compound **9** (200 mg, 0.2 mmol) and (2-aminopyridin-4-yl)­boronic acid (69 mg, 0.5 mmol) in THF-H_2_O (0.2 M, 10:1) was added K_2_CO_3_ (138 mg, 1.0 mmol) and the reaction mixture was degassed with argon for 15 min in a thick-well borosilicate glass vial. Pd­(PPh_3_)_4_ (0.1 mmol, 10 mol %) was then added and the reaction mixture was degassed again for 15 min and irradiated in the *M*_W_ at 130 °C for 30 min. Then the reaction mixture was cooled to rt and concentrated. The crude compound was purified by preparative HPLC to afford the corresponding coupled product and used directly in the Suzuki step.

LCMS: Rt 2.50 min, (ESI^–^) *m*/*z* 833.2, [M – H]^−^, Purity 97%.

^1^H NMR (400 MHz, DMSO-*d*_6_) δ 12.60 (br s, 1H), 11.46 (br s, 2H), 8.86 (s, 1H), 8.60 (d, *J* = 2.5 Hz, 1H), 8.49 (d, *J* = 8.8 Hz, 2H), 8.06 (d, *J* = 5.2 Hz, 1H), 7.59 (s, 1H), 7.42 (br s, 2H), 7.24 (d, *J* = 5.5 Hz, 1H), 6.97–6.86 (m, 3H), 6.41 (br s, 1H), 6.16 (s, 2H), 4.23 (m, 1H), 3.13–3.07 (m, 2H), 2.93 (s, 2H), 2.48 (s, 3H), 2.42 (s, 3H), 1.98 (s, 3H), 1.84–1.74 (m, 1H), 1.68 (m, 1H), 1.60–1.40 (m, 2H), 1.39 (s, 6H).

#### N2-(3-((6-Aminoquinoline)-8-sulfonamido)­thiophene-2-carbonyl)-Nw-((2,2,4,6,7-pentamethyl-2,3-dihydrobenzofuran-5-yl)­sulfonyl)-l-arginine (**13**)

To a solution of Compound **9** (600 mg, 0.7 mmol) in DMSO (8 mL, 0.1 M) was added NaN_3_ (118 mg, 1.8 mmol), Na_2_CO_3_ (100 mg, 0.9 mmol) and CuI (173 mg, 0.9 mmol) at rt. The mixture was degassed for 20 min with argon and then was added DMEDA (112 mg, 1.3 mmol) and heated at 110 °C for 1 h. The reaction mixture was cooled to rt, diluted with EtOAc (80 mL), washed with 1 N HCl (40 mL), dried over anhydrous Na_2_SO_4_ and concentrated. The crude compound was purified by preparative HPLC to afford **13** (160 mg, 29%) as a yellow solid.

LCMS: Rt 3.12 min, (ESI^–^) *m*/*z* 756.3, [M-H]^−^, Purity 96%.

^1^H NMR (400 MHz, DMSO-*d*_6_): δ 12.68 (br s, 1H), 11.21 (br s, 1H), 8.43 (s, 1H), 8.21 (br s, 1H), 7.97 (dd, *J* = 8.5, 1.7 Hz, 1H), 7.91 (d, *J* = 2.6 Hz, 1H), 7.57 (br s, 1H), 7.28 (br s, 1H), 7.20 (d, *J* = 5.4 Hz, 1H), 6.97 (s, 1H), 6.75 (br s, 1H), 6.41 (br s, 2H), 6 (s, 2H), 4.27 (m, 1H), 3.08–2.98 (m, 2H), 2.92 (s, 2H), 2.48 (s, 3H), 2.42 (s, 3H), 1.97 (s, 3H), 1.79–1.75 (m, 1H), 1.74–1.65 (m, 1H), 1.38 (s, 8H).

#### N2-(3-((6-(((2-(Dimethylamino)­thiazol-4-yl)­methyl)­amino)­quinoline)-8-sulfonamido)­thiophene-2-carbonyl)-Nw-((2,2,4,6,7-pentamethyl-2,3-dihydrobenzofuran-5-yl)­sulfonyl)-l-arginine (**11g**)

To a solution of compound **13** (100 mg, 0.1 mmol) in THF-MeOH (3 mL, 0.04 M, 1:1) were added 2-(dimethylamino)­thiazole-4-carbaldehyde (15.6 mg, 0.1 mmol), magnesium sulfate (0.2 mmol) and AcOH (0.1 mmol) at 0 °C and the reaction mixture was stirred at rt for 1 h. NaCNBH_3_ (0.1 mmol) was then added, and the reaction mixture was stirred for further 2 h. The reaction mixture was quenched with ice water (10 mL) and extracted with EtOAc (2 × 10 mL). The combined organic layer was washed successively with water (20 mL) and brine solution (20 mL), dried over anhydrous Na_2_SO_4_ and concentrated. The crude compound was used directly in the next step without purification (70 mg, yellow solid).

#### Nw-((2,2,4,6,7-Pentamethyl-2,3-dihydrobenzofuran-5-yl)­sulfonyl)-N2-(3-((6-(((2-(piperazin-1-yl)­thiazol-4-yl)­methyl)­amino)­quinoline)-8-sulfonamido)­thiophene-2-carbonyl)-l-arginine (**11h**)

To a solution of compound **13** (100 mg, 0.1 mmol) in THF-MeOH (3 mL, 0.04 M, 1:1) were added 2-(piperazin-1-yl)­thiazole-4-carbaldehyde (19.7 mg, 0.1 mmol), magnesium sulfate (0.2 mmol) and AcOH (0.1 mmol) at 0 °C and the reaction mixture was stirred at rt for 1 h. NaCNBH_3_ (0.1 mmol) was then added, and the reaction mixture was stirred for further 2 h. The reaction mixture was quenched with ice water (10 mL) and extracted with EtOAc (2 × 10 mL). The combined organic layer was washed successively with water (20 mL) and brine solution (20 mL), dried over anhydrous Na_2_SO_4_ and concentrated. The crude compound was used directly in the next step without purification (85 mg, yellow solid).

#### N2-(3-((6-(((2-Aminothiazol-4-yl)­methyl)­amino)­quinoline)-8-sulfonamido)­thiophene-2-carbonyl)-Nw-((2,2,4,6,7-pentamethyl-2,3-dihydrobenzofuran-5-yl)­sulfonyl)-l-arginine (**11i**)

To a solution of compound **13** (100 mg, 0.1 mmol) in THF-MeOH (3 mL, 0.04 M, 1:1) were added 2-aminothiazole-4-carbaldehyde (12.8 mg, 0.1 mmol), magnesium sulfate (0.2 mmol) and AcOH (0.1 mmol) at 0 °C and the reaction mixture was stirred at rt for 1 h. NaCNBH_3_ (0.1 mmol) was then added, and the reaction mixture was stirred for further 2 h. The reaction mixture was quenched with ice water (10 mL) and extracted with EtOAc (2 × 10 mL). The combined organic layer was washed successively with water (20 mL) and brine solution (20 mL), dried over anhydrous Na_2_SO_4_ and concentrated. The crude compound was used directly in the next step without purification (135 mg, yellow solid).

#### N2-(3-((6-(((2-(Methylamino)­thiazol-4-yl)­methyl)­amino)­quinoline)-8-sulfonamido)­thiophene-2-carbonyl)-Nw-((2,2,4,6,7-pentamethyl-2,3-dihydrobenzofuran-5-yl)­sulfonyl)-l-arginine (**11j**)

To a solution of compound **13** (100 mg, 0.1 mmol) in THF-MeOH (3 mL, 0.04 M, 1:1) were added 2-(methylamino)­thiazole-4-carbaldehyde (14.2 mg, 0.1 mmol), magnesium sulfate (0.2 mmol) and AcOH (0.1 mmol) at 0 °C and the reaction mixture was stirred at rt for 1 h. NaCNBH_3_ (0.1 mmol) was then added, and the reaction mixture was stirred for further 2 h. The reaction mixture was quenched with ice water (10 mL) and extracted with EtOAc (2 × 10 mL). The combined organic layer was washed successively with water (20 mL) and brine solution (20 mL), dried over anhydrous Na_2_SO_4_ and concentrated. The crude compound was used directly in the next step without purification (73 mg, yellow solid).

#### N2-(3-((6-(((6-Morpholinopyridin-2-yl)­methyl)­amino)­quinoline)-8-sulfonamido)­thiophene-2-carbonyl)-Nw-((2,2,4,6,7-pentamethyl-2,3-dihydrobenzofuran-5-yl)­sulfonyl)-l-arginine (**11k**)

To a solution of compound **13** (100 mg, 0.1 mmol) in THF-MeOH (3 mL, 0.04 M, 1:1) were added 6-morpholinopicolinaldehyde (19.2 mg, 0.1 mmol), magnesium sulfate (0.2 mmol) and AcOH (0.1 mmol) at 0 °C and the reaction mixture was stirred at rt for 1 h. NaCNBH_3_ (0.1 mmol) was then added, and the reaction mixture was stirred for further 2 h. The reaction mixture was quenched with ice water (10 mL) and extracted with EtOAc (2 × 10 mL). The combined organic layer was washed successively with water (20 mL) and brine solution (20 mL), dried over anhydrous Na_2_SO_4_ and concentrated. The crude compound was used directly in the next step without purification (70 mg, yellow solid).

#### N2-(3-((6-(((6-Aminopyridin-3-yl)­methyl)­amino)­quinoline)-8-sulfonamido)­thiophene-2-carbonyl)-Nw-((2,2,4,6,7-pentamethyl-2,3-dihydrobenzofuran-5-yl)­sulfonyl)-l-arginine (**11l**)

To a solution of compound **13** (100 mg, 0.1 mmol) in THF-MeOH (3 mL, 0.04 M, 1:1) were added 6-aminonicotinaldehyde (12.2 mg, 0.1 mmol), magnesium sulfate (0.2 mmol) and AcOH (0.1 mmol) at 0 °C and the reaction mixture was stirred at rt for 1 h. NaCNBH_3_ (0.1 mmol) was then added, and the reaction mixture was stirred for further 2 h. The reaction mixture was quenched with ice water (10 mL) and extracted with EtOAc (2 × 10 mL). The combined organic layer was washed successively with water (20 mL) and brine solution (20 mL), dried over anhydrous Na_2_SO_4_ and concentrated. The crude compound was used directly in the next step without purification (51 mg, orange solid).

#### N2-(3-((6-(((2-(Dimethylamino)­pyrimidin-5-yl)­methyl)­amino)­quinoline)-8-sulfonamido)­thiophene-2-carbonyl)-Nw-((2,2,4,6,7-pentamethyl-2,3-dihydrobenzofuran-5-yl)­sulfonyl)-l-arginine (**11m**)

To a solution of compound **13** (100 mg, 0.1 mmol) in THF-MeOH (3 mL, 0.04 M, 1:1) were added 2-(dimethylamino)­pyrimidine-5-carbaldehyde (15.1 mg, 0.1 mmol), magnesium sulfate (0.2 mmol) and AcOH (0.1 mmol) at 0 °C and the reaction mixture was stirred at rt for 1 h. NaCNBH_3_ (0.1 mmol) was then added, and the reaction mixture was stirred for further 2 h. The reaction mixture was quenched with ice water (10 mL) and extracted with EtOAc (2 × 10 mL). The combined organic layer was washed successively with water (20 mL) and brine solution (20 mL), dried over anhydrous Na_2_SO_4_ and concentrated. The crude compound was used directly in the next step without purification (25 mg, orange solid).

#### N2-(3-((6-(((6-(Dimethylamino)­pyridin-3-yl)­methyl)­amino)­quinoline)-8-sulfonamido)­thiophene-2-carbonyl)-Nw-((2,2,4,6,7-pentamethyl-2,3-dihydrobenzofuran-5-yl)­sulfonyl)-l-arginine (**11n**)

To a solution of compound **13** (100 mg, 0.1 mmol) in THF-MeOH (3 mL, 0.04 M, 1:1) were added 6-(dimethylamino)­nicotinaldehyde (15.0 mg, 0.1 mmol), magnesium sulfate (0.2 mmol) and AcOH (0.1 mmol) at 0 °C and the reaction mixture was stirred at rt for 1 h. NaCNBH_3_ (0.1 mmol) was then added, and the reaction mixture was stirred for further 2 h. The reaction mixture was quenched with ice water (10 mL) and extracted with EtOAc (2 × 10 mL). The combined organic layer was washed successively with water (20 mL) and brine solution (20 mL), dried over anhydrous Na_2_SO_4_ and concentrated. The crude compound was used directly in the next step without purification (43 mg, orange solid).

#### Nw-((2,2,4,6,7-Pentamethyl-2,3-dihydrobenzofuran-5-yl)­sulfonyl)-N2-(3-(quinoline-8-sulfonamido)­thiophene-2-carbonyl)-l-arginine (**11o**)

A solution of 8-quinolinesulfonyl chloride (1.8 g, 7.9 mmol) in pyridine (10 mL) was added dropwise to a stirring solution of methyl-3-amino-2-thiophene carboxylate (1 g, 6.3 mmol) in pyridine (10 mL). The solution was stirred for 18 h during which time a faint solid had formed. H_2_O (30 mL) was added and the resultant off-white solid that precipitated was collected by filtration and washed with H_2_O. LCMS analysis confirmed the solid was desired intermediate methyl ester. The solid (1.4 g, 4.0 mmol) was redissolved in THF/MeOH (2:1, 21 mL) and LiOH (aq., 1M, 20 mL, 20 mmol) added in one portion. The reaction was heated at 65 °C for 5 h after which time the reaction was deemed complete by LCMS. The solvent was removed *in vacuo* and the residue dissolved in H_2_O (30 mL) before acidifying to pH2 using HCl (aq., 2N). The product was extracted with DCM (2 × 50 mL) and the combined organic extracts dried over MgSO_4_ before removing the solvent under reduced pressure to give the desired compound as an off-white solid, 1.25 g, 3.7 mmol, 59%. (LCMS: Rt 2.12 min, (ESI^+^) *m*/*z* 335, [M + H]^+^, Purity 99%.) This acid (300 mg, 0.89 mmol) dissolved in DCM (40 mL) and DIPEA (0.48 mL, 2.7 mmol) and HATU (418 mg, 1.1 mmol) were added, and the solution stirred at ambient temperature for 10 min before adding H-l-Arg­(Pbf)-OMe (390 mg, 0.89 mmol) in one portion. The reaction was stirred at ambient temperature for 18 h after which time LCMS indicated the reaction had gone to completion. The reaction mixture was diluted with NaHO_3_ (sat., aq., 50 mL) and DCM (100 mL) and the layers separated. The organic phase was dried over MgSO_4_ before removing the solvent *in vacuo*. Purification by column chromatography (Biotage SP1, KP-Sil column eluting with 100% DCM to 10% MeOH/DCM) provided the title compound as a pale-yellow oil (500 mg, 0.67 mmol, 74%). (LCMS: Rt 2.91 min, (ESI^+^) *m*/*z* 757, [M + H]^+^, Purity 64%). This protected arginine mimetic (1 equiv) was dissolved in THF/H_2_O (4:1, 0.04 M) and LiOH (1M, aq., 5 equiv) added in one portion. The reaction was stirred at rt until LCMS indicated the reaction had gone to completion. The reaction was then concentrated *in vacuo* and the residue taken up in TFA/DCM (1:1, excess) and stirred at rt until LCMS indicated the reaction had gone to completion. The TFA was removed *in vacuo* to provide the crude product as a viscous oil. Purification by preparative HPLC (pH9) afforded the title compound (74 mg, 23%, cream solid).

#### (8-(N-(2-(Methoxycarbonyl)­thiophen-3-yl)­sulfamoyl)­quinolin-6-yl)­boronic Acid (**19**)

Bromoquinoline **9** (600 mg, 1.4 mmol), bispinacolato diboron (720 mg, 2.8 mmol), Pd­(dppf)_2_Cl_2_ (102 mg, 0.14 mmol) and KOAc (414 mg, 4.2 mmol) were combined and suspended in dioxane (15 mL). The suspension was degassed with nitrogen for 5 min before heating in a microwave at 100 °C for 10 min. The reaction was filtered through a pad of Celite and concentrated *in vacuo* to provide the crude product as a dark brown oil (1.4 g). The crude material was taken into the subsequent Suzuki couplings.

LCMS: Rt 2.32 min, (ESI^+^) *m*/*z* 393, [M + H]^+^, Purity 81%1%.

#### Methyl 3-((6-(2-(Dimethylamino)­thiazol-4-yl)­quinoline)-8-sulfonamido)­thiophene-2-carboxylate (**20p**)

Boronic acid **19** (crude from previous step, assumed 1.4 mmol), 4-bromo-N,N-dimethylthiazol-2-amine (315 mg, 1.5 mmol), Pd­(PPh_3_)_4_ (162 mg, 0.14 mmol) and K_3_PO_4_ (2 M, aq., 2.7 mL) were combined and suspended in DME (15 mL). The reaction was degassed with nitrogen for 5 min before heating in the microwave at 120 °C for 20 min. LCMS indicated completion of reaction. The reaction mixture was acidified to pH5 using 10% AcOH/H_2_O and then concentrated *in vacuo* to give the crude product. Purification by column chromatography (Biotage SP1, KP-Sil column eluting with neat iso-hexane to 10% MeOH/EtOAc) provided the title compound as a pale brown solid, 490 mg, 1.0 mmol, 73% over 2 steps.

LCMS AnalpH9_MeOH_QC: Rt 3.15 min, (ESI^+^) *m*/*z* 475, [M + H]^+^, Purity 87%.

#### Methyl 3-((6-(2-(Dimethylamino)­thiazol-4-yl)­quinoline)-8-sulfonamido)­thiophene-2-carboxylate (**21p**)

Methyl ester **20p** (487 mg, 1.0 mmol) was dissolved in THF/MeOH (2:1, 9 mL) and LiOH (1 M, aq., 5.1 mL, 5.1 mmol) added in one portion. The reaction was stirred at 50 °C for 16 h whereupon LCMS indicated the reaction had gone to completion. The reaction was concentrated *in vacuo* and the residue taken up in H_2_O (30 mL) and washed with EtOAc (1 × 30 mL). The aqueous layer was then acidified to pH2 using HCl (6 M, aq.) and further extracted with EtOAc (3 × 30 mL). The combined organic extracts were washed with brine (30 mL), dried over MgSO_4_ and concentrated *in vacuo* to give the title compound as an orange solid, 371 mg, 0.81 mmol, 78%

LCMS: Rt 3.02 min, (ESI^+^) *m*/*z* 461, [M + H]^+^, Purity 83%.

#### N2-(3-((6-(2-(Dimethylamino)­thiazol-4-yl)­quinoline)-8-sulfonamido)­thiophene-2-carbonyl)-Nw-((2,2,4,6,7-pentamethyl-2,3-dihydrobenzofuran-5-yl)­sulfonyl)-l-arginine (**22p**)

Carboxylic acid **21a** (365 mg, 0.79 mmol) and PyBrOP (554 mg, 1.2 mmol) were suspended in DCM (4 mL) and stirred at rt for 5 min before adding DIPEA (1 mL, 5.6 mmol) in one portion, whereupon the suspension gave way to a dark yellow solution. The reaction was stirred at rt for 6 days, at which point LCMS indicated the reaction had gone to completion. The reaction was concentrated in vacuo to provide the crude product. Purification by prep-HPLC (pH9) provided the title compound as an orange oil, 300 mg, 0.34 mmol, 43%

LCMS: Rt 3.22 min, (ESI^+^) *m*/*z* 883, [M + H]^+^, Purity 92%.

#### Methyl 3-((6-(2-Aminothiazol-4-yl)­quinoline)-8-sulfonamido)­thiophene-2-carboxylate (**20q**)

Synthesis as for **20a**, but with 4-bromothiazol-2-amine (550 mg, 1.4 mmol). Purification by prep-HPLC (pH9) provided the title compound as a white solid, 152 mg, 0.28 mmol, 20% over 2 steps.

LCMS: Rt 3.26 min, (ESI^+^) *m*/*z* 547, [M + H]^+^, Purity 98%.

#### ((6-(2-Aminothiazol-4-yl)­quinoline)-8-sulfonamido)­thiophene-2-carboxylic Acid (**21q**)

Synthesis as for **21p** but starting from **20q** (150 mg, 0.27 mmol). The title compound was isolated as a pale-yellow solid, 96 mg, 0.18 mmol, 67%.

LCMS: Rt 3.20 min, (ESI^+^) *m*/*z* 533, [M + H]^+^, Purity 84%.

#### N2-(3-((6-(2-Aminothiazol-4-yl)­quinoline)-8-sulfonamido)­thiophene-2-carbonyl)-Nw-((2,2,4,6,7-pentamethyl-2,3-dihydrobenzofuran-5-yl)­sulfonyl)-l-arginine (**22q**)

Synthesis as for **22p** but starting from **21q** (94 mg, 0.18 mmol). The title compound was isolated as a white solid, 41 mg, 0.043 mmol, 24%.

LCMS: Rt 3.30 min, (ESI^+^) *m*/*z* 955, [M + H]^+^, Purity 98%.

### General Pbf Removal Procedure, **12a–n**

To a solution of key intermediate (0.01 mmol) in DCM (0.1 M) was added TFA (1.5 mmol) and the reaction mixture was stirred at rt for 20 h. The reaction mixture was evaporated, and the crude compound was purified by preparative HPLC (pH2) to afford the target compounds.

#### (2*S*)-5-Guanidino-2-[[3-[[6-[3-[[(3-methylimidazol-4-yl)­methylamino]­methyl]­phenyl]-8-quinolyl]­sulfonylamino]­thiophene-2-carbonyl]­amino]­pentanoic Acid, Formic Acid **12a**

LCMS: Rt 3.47 min, (ESI^+^) *m*/*z* 690.2, [M + H]^+^, Purity 100%. Ten mg, 55%, yellow solid.

#### (2*S*)-5-Guanidino-2-[[3-[[6-[4-(methylaminomethyl)­phenyl]-8-quinolyl]­sulfonylamino]­thiophene-2-carbonyl]­amino]­pentanoic Acid, Formic Acid **12b**

LCMS: Rt 4.09 min, (ESI^+^) *m*/*z* 610.2, [M + H]^+^, Purity 100%. Five mg, 30%, white solid.

#### (2*S*)-5-Guanidino-2-[[3-[[6-[4-[[(3-methylimidazol-4-yl)­methylamino]­methyl]­phenyl]-8-quinolyl]­sulfonylamino]­thiophene-2-carbonyl]­amino]­pentanoic Acid, Formic Acid **12c**

LCMS: Rt 4.73 min, (ESI^+^) *m*/*z* 646.2, [M + H]^+^, Purity 95%. Twenty-eight mg, 74%, yellow solid.

^1^H NMR (400 MHz, DMSO-*d*_6_) δ 8.88 (dd, *J* = 4.0, 2.0 Hz, 1H), 8.64 (d, *J* = 2.4 Hz, 1H), 8.47 (dd, *J* = 8.4, 2.0 Hz, 1H), 8.41 (d, *J* = 2.4 Hz, 1H), 8.14 (s, 1H), 7.77 (d, *J* = 8.4 Hz, 2H), 7.61–7.53 (m, 4H), 7.26 (d, *J* = 5.6 Hz, 1H), 7.20 (d, *J* = 5.6 Hz, 1H), 6.83 (s, 1H), 4.23–4.20 (m, 1H), 3.82 (s, 2H), 3.75 (s, 2H), 3.64 (s, 3H),3.33–3.26 (m, 2H), 1.92–1.78 (m, 4H).

#### (2*S*)-2-[[3-[[6-[3-(Aminomethyl)­phenyl]-8-quinolyl]­sulfonylamino]­thiophene-2-carbonyl]­amino]-5-guanidino-pentanoic Acid, Formic Acid **12d**

LCMS: Rt 4.09 min, (ESI^+^) *m*/*z* 596.2, [M + H]^+^, Purity 100%. 39 mg, 76%, white solid.

^1^H NMR (400 MHz, DMSO-*d*_6_) δ 10.08 (br s, 1H), 8.89 (s, 1H), 8.59 (s, 1H), 8.39 (d, *J* = 8.0 Hz, 1H), 8.24–8.21 (m, 2H), 7.94 (s, 1H), 7.74 (d, *J* = 8.0 Hz, 1H), 7.54–7.48 (m, 2H), 7.43 (d, *J* = 8.0 Hz, 1H), 7.25 (br s, 1H), 7.14 (d, *J* = 5.6 Hz, 1H), 4.21 (br s, 1H), 4.09–4.01 (m, 2H), 3.18–3.17 (m, 2H), 1.82–1.60 (m, 4H).

#### (2*S*)-2-[[3-[[6-[4-(Aminomethyl)­phenyl]-8-quinolyl]­sulfonylamino]­thiophene-2-carbonyl]­amino]-5-guanidino-pentanoic Acid, Formic Acid **12e**

LCMS: Rt 3.93 min, (ESI^+^) *m*/*z* 596.2, [M + H]^+^, Purity 100%.

^1^H NMR (400 MHz, DMSO-*d*_6_) δ 10.18 (br s, 1H), 8.91 (dd, *J* = 4.0, 1.6 Hz, 1H), 8.63 (d, *J* = 2.0 Hz, 1H), 8.47 (dd, *J* = 8.4, 1.6 Hz, 1H), 8.16 (s, 1H), 7.85 (d, *J* = 8.0 Hz, 1H), 7.64–7.58 (m, 2H), 7.23 (d, *J* = 5.2 Hz, 1H), 7.19 (d, *J* = 5.2 Hz, 1H), 6.57 (br s, 1H), 4.21 (br s, 1H), 4.11 (s, 2H), 2.69–2.67 (m, 2H), 1.90–1.81 (m, 4H).

#### (2*S*)-2-[[3-[[6-(2-Amino-4-pyridyl)-8-quinolyl]­sulfonylamino]­thiophene-2-carbonyl]­amino]-5-guanidino-pentanoic Acid, Formic Acid **12f**

LCMS: Rt 3.65 min, (ESI^+^) *m*/*z* 583.2, [M + H]^+^, Purity 100%. Fourteen mg, 72%, yellow solid.

^1^H NMR (400 MHz, DMSO-*d*_6_) δ δ 10.19–10.02 (m, 1H), 8.90 (dd, *J* = 4.4, 2.0 Hz, 1H), 8.64 (d, *J* = 2.4 Hz, 1H), 8.49 (dd, *J* = 8.8, 2.0 Hz, 1H), 8.42 (d, *J* = 2.4 Hz, 1H), 8.14 (s, 1H), 8.04 (d, *J* = 5.2 Hz, 1H), 7.61 (dd, *J* = 8.8, 4.4 Hz, 1H), 7.54 (t, *J* = 5.2 Hz, 1H), 7.25 (d, *J* = 5.2 Hz, 1H), 7.18 (d, *J* = 5.2 Hz, 1H), 6.91 (dd, *J* = 5.2, 1.6 Hz, 1H), 6.88–6.87 (m, 1H), 6.15 (s, 2H), 4.22–4.21 (m, 1H), 1.93–1.80 (m, 4H).

#### (2*S*)-2-[[3-[[6-[[2-(Dimethylamino)­thiazol-4-yl]­methylamino]-8-quinolyl]­sulfonylamino]­thiophene-2-carbonyl]­amino]-5-guanidino-pentanoic Acid, Formic Acid **12g** **

LCMS: Rt 4.73 min, (ESI^+^) *m*/*z* 646.2, [M + H]^+^, Purity 95%. 2 mg, nominal amount part of sample lost.

^1^H NMR (400 MHz, DMSO-*d*_6_) δ 8.44 (dd, *J* = 4.4, 2.0 Hz, 1H), 8.16 (s, 1H), 8.08 (d, *J* = 2.4 Hz, 1H), 8.00 (dd, *J* = 8.4, 2.0 Hz, 1H), 7.66 (br s, 1H), 7.30 (dd, *J* = 8.4, 4.4 Hz, 1H), 7.25 (d, *J* = 5.6 Hz, 1H), 7.10 (d, *J* = 5.6 Hz, 1H), 6.93 (t, *J* = 5.6 Hz, 1H), 6.82 (d, *J* = 2.4 Hz, 1H), 6.50 (s, 1H), 4.22 (d, *J* = 5.6 Hz, 2H), 4.15–4.13 (m, 1H), 3.33–3.32 (m, 2H), 3.02 (s, 6H), 1.88–1.83 (m, 4H).

#### (2*S*)-5-Guanidino-2-[[3-[[6-[(2-piperazin-1-ylthiazol-4-yl)­methylamino]-8-quinolyl]­sulfonylamino]­thiophene-2-carbonyl]­amino]­pentanoic Acid, Formic Acid **12h**

LCMS: Rt 3.93 min, (ESI^+^) *m*/*z* 688.2, [M + H]^+^, Purity 98%. Twenty-six mg, 46%, yellow solid.

^1^H NMR (400 MHz, DMSO-*d*_6_) δ 9.86 (br s, 1H), 8.44 (dd, *J* = 4.0, 1.6 Hz, 1H), 8.16 (s, 1H), 8.08 (d, *J* = 2.8 Hz, 1H), 8.00 (dd, *J* = 8.4, 1.6 Hz, 1H), 7.67 (br s, 1H), 7.30 (dd, *J* = 8.4, 4.0 Hz, 1H), 7.26 (d, *J* = 5.6 Hz, 1H), 7.11 (d, *J* = 5.6 Hz, 1H), 6.93 (t, *J* = 6.4 Hz, 1H), 6.82 (d, *J* = 2.4 Hz, 1H), 6.61 (s, 1H), 4.23 (d, *J* = 5.6 Hz, 2H), 4.15–4.13 (m, 1H), 3.39–3.36 (m, 6H), 2.91–2.89 (m, 4H), 1.91–1.79 (m, 4H).

HRMS Calc. for C_14_H_15_BrN_2_O_2_ [M + H]^+^ 323.03897 found 323.0383.

#### (2*S*)-2-[[3-[[6-[(2-Aminothiazol-4-yl)­methylamino]-8-quinolyl]­sulfonylamino]­thiophene-2-carbonyl]­amino]-5-guanidino-pentanoic Acid, Formic Acid **12i**

LCMS: Rt 3.79 min, (ESI^+^) *m*/*z* 618.2, [M + H]^+^, Purity 99%. 41 mg, 55%, yellow solid.

^1^H NMR (400 MHz, DMSO-*d*_6_) δ 8.44 (dd, *J* = 4.0, 1.6 Hz, 1H), 8.16 (s, 1H), 8.06 (d, *J* = 2.4 Hz, 1H), 8.00 (dd, *J* = 8.4, 1.6 Hz, 1H), 7.69 (br s, 1H), 7.31 (dd, *J* = 8.4, 4.4 Hz, 1H), 7.28 (d, *J* = 6.8 Hz, 1H), 7.11 (d, *J* = 5.6 Hz, 1H), 6.90–6.87 (m, 1H), 6.81 (d, *J* = 2.4 Hz, 1H), 6.33 (s, 1H), 4.16–4.15 (m, 3H), 3.39–3.36 (m, 6H), 3.39–3.25 (m, 6H), 1.91–1.78 (m, 4H).

#### (2*S*)-5-Guanidino-2-[[3-[[6-[[2-(methylamino)­thiazol-4-yl]­methylamino]-8-quinolyl]­sulfonylamino]­thiophene-2-carbonyl]­amino]­pentanoic Acid **12j**

LCMS: Rt 4.49 min, (ESI^+^) *m*/*z* 632.2, [M + H]^+^, Purity 99%. Nine mg, 37%, yellow solid.

#### (2S)-5-Guanidino-2-[[3-[[6-[(6-morpholino-2-pyridyl)­methylamino]-8-quinolyl]­sulfonylamino]­thiophene-2-carbonyl]­amino]­pentanoic Acid **12k**

LCMS: Rt 5.31 min, (ESI^+^) *m*/*z* 683.2, [M + H]^+^, Purity 98%. Twenty-four mg, 55%, yellow solid.

^1^H NMR (400 MHz, DMSO-*d*_6_) δ 8.45 (dd, *J* = 4.0, 1.6 Hz, 1H), 8.14 (s, 1H), 8.01 (d, *J* = 2.4 Hz, 1H), 7.98 (dd, *J* = 8.4, 1.6 Hz, 1H), 7.59 (br s, 1H), 7.49 (dd, *J* = 8.0, 7.2 Hz, 1H), 7.36 (br s, 1H), 7.31 (dd, *J* = 8.0, 4.4 Hz, 1H), 7.13–7.10 (m, 2H), 6.82 (br s, 1H), 6.68 (d, *J* = 7.2 Hz, 1H), 6.66 (d, *J* = 7.2 Hz, 1H), 4.31 (d, *J* = 6.0 Hz, 2H), 4.20 (br s, 1H), 3.70–3.68 (m, 4H), 3.47–3.45 (m, 4H), 3.28–3.23 (m, 2H), 1.88–1.79 (m, 4H).

#### (2*S*)-2-[[3-[[6-[(6-Amino-3-pyridyl)­methylamino]-8-quinolyl]­sulfonylamino]­thiophene-2-carbonyl]­amino]-5-guanidino-pentanoic Acid, Formic Acid **12l**

LCMS: Rt 3.75 min, (ESI^+^) *m*/*z* 612.2, [M + H]^+^, Purity 97%. Nine mg,(22%) 22%, yellow solid.

^1^H NMR (400 MHz, DMSO-*d*_6_) δ 8.44 (dd, *J* = 4.0, 1.6 Hz, 1H), 8.14 (s, 1H), 8.02–8.00 (m, 2H), 7.95 (d, *J* = 1.6 Hz, 1H), 7.55 (br s, 1H), 7.39 (dd, *J* = 8.0, 2.4 Hz, 1H), 7.32 (dd, *J* = 8.0, 4.0 Hz, 1H), 7.08 (d, *J* = 5.6 Hz, 1H), 6.92 (br s, 1H), 6.84 (br s, 1H), 6.55 (s, 1H), 6.41 (d, *J* = 8.0 Hz, 1H), 5.85 (s, 1H), 4.20 (br s, 1H), 4.15 (d, *J* = 6.0 Hz, 2H), 3.26–3.24 (m, 2H), 1.91–1.72 (m, 4H).

#### (2*S*)-2-[[3-[[6-[[2-(Dimethylamino)­pyrimidin-5-yl]­methylamino]-8-quinolyl]­sulfonylamino]­thiophene-2-carbonyl]­amino]-5-guanidino-pentanoic Acid **12m**

LCMS: Rt 5.48 min, (ESI^+^) *m*/*z* 642.2, [M + H]^+^, Purity 98%. Two mg, 5%, yellow solid.

#### (2*S*)-2-[[3-[[6-[[6-(Dimethylamino)-3-pyridyl]­methylamino]-8-quinolyl]­sulfonylamino]­thiophene-2-carbonyl]­amino]-5-guanidino-pentanoic Acid **12n**

LCMS: Rt 3.68 min, (ESI^+^) *m*/*z* 640.2, [M + H]^+^, Purity 99%. Eight mg, 5%, yellow solid.

### General Methyl Ester Hydrolysis and Pbf Removal Procedure, **12o–q**

Protected intermediate (1 equiv) was dissolved in THF/H_2_O (4:1, 0.04 M) and LiOH (1 M, aq., 5 equiv) added in one portion. The reaction was stirred at rt until LCMS indicated the reaction had gone to completion. The reaction was then concentrated *in vacuo* and the residue taken up in TFA/DCM (1:1, excess) and stirred at rt until LCMS indicated the reaction had gone to completion. The TFA was removed *in vacuo* to provide the crude product as a viscous oil. Purification by preparative HPLC (pH9) afforded the title compound.

#### (S)-5-Guanidino-2-{[3-(quinoline-8-sulfonylamino)-thiophene-2-carbonyl]-amino}-pentanoic Acid, **12o**

LCMS: Rt 4.94 min, (ESI^+^) *m*/*z* 491, [M + H]^+^, Purity 99%. 74 mg, 23%, cream solid.

^1^H NMR (400 MHz, DMSO-*d*_6_) δ 9.52 (br s, 1H), 8.90 (dd, *J* = 4.0, 1.5 Hz, 1H), 8.42 (dd, *J* = 8.6, 1.8 Hz, 1H), 8.38 (dd, *J* = 7.3, 1.5 Hz, 1H), 8.12 (dd, *J* = 8.3, 1.0 Hz, 1H), 7.76 (br s, 1H), 7.66 (app t, *J* = 7.6 Hz, 1H), 7.58 (dd, *J* = 8.3, 4.0 Hz, 1H), 7.38 (br s, 1H), 7.29 (d, *J* = 5.6 Hz, 1H), 7.15 (d, *J* = 5.6 Hz, 1H), 7.10–6.80 (br s, 2H), 4.20 (m, 1H), 3.28–3.18 (m, 2H), 1.88 (m, 1H), 1.82–1.62 (m, 3H).

#### (S)-2-({3-[6-(2-Dimethylamino-thiazol-4-yl)-quinoline-8-sulfonylamino]-thiophene-2-carbonyl}-amino)-5-guanidino-pentanoic Acid **12p**

LCMS: Rt 6.15 min, (ESI^+^) *m*/*z* 617, [M + H]^+^, Purity 99%. 49 mg, 23%, yellow solid.

^1^H NMR (400 MHz, DMSO-*d*_6_) δ 13.18–11.84 (br s, 2H), 9.97 (br s, 1H), 8.83 (m, 2H), 8.49 (d, *J* = 2.0 Hz, 1H), 8.43 (dd, *J* = 8.3, 1.8 Hz, 1H), 7.63 (br s, 1H), 7.55 (dd, *J* = 8.3, 4.3 Hz, 1H), 7.51–7.19 (br s, 2H), 7.40 (s, 1H), 7.24 (d, *J* = 5.6 Hz, 1H), 7.16 (d, *J* = 5.6 Hz, 1H), 6.92–6.68 (br s, 2H), 4.20 (m, 1H), 3.32–3.24 (m, 2H), 3.13 (s, 6H), 1.96–1.72 (m, 4H).

#### (S)-2-({3-[6-(2-Amino-thiazol-4-yl)-quinoline-8-sulfonylamino]­thiophene-2-carbonyl}-amino)-5-guanidino-pentanoic Acid **12q**

LCMS: Rt 4.88 min, (ESI^+^) *m*/*z* 589, [M + H]^+^, Purity 99%. Twenty mg, 80%, pale yellow solid.

^1^H NMR (400 MHz, DMSO-*d*_6_) δ 9.98 (br s, 1H), 8.86 (d, *J* = 2.3 Hz, 1H), 8.81 (dd, *J* = 4.3, 1.8 Hz, 1H), 8.42 (d, *J* = 2.0 Hz, 1H), 8.39 (dd, *J* = 8.3, 1.8 Hz, 1H), 7.66 (br s, 1H), 7.54 (dd, *J* = 8.3, 4.3 Hz, 1H), 7.45–7.19 (br s, 2H), 7.27–7.21 (m, 4H), 7.13 (d, *J* = 5.6 Hz, 1H), 6.96–6.51 (br s, 2H), 4.18 (m, 1H), 3.33–3.25 (m, 2H), 1.95–1.75 (m, 4H).

### Alternative Route to **12h**

#### *tert*-Butyl (8-bromoquinolin-6-yl)­carbamate (**24**)

In a 500 mL rb flask with an air condenser. To the 6-nitroquinoline **23** (9.0 g, 0.052 mol, 1.0 equiv, 6-nitroquinoline) was added sulfuric acid (45.0 mL, 0.81 mol, 15.7 equiv) and the reaction stirred at 60 °C until in solution (approximately 10 min). The NBS (18.4 g, 0.10 mol, 2.0 equiv) was added in portions and the reaction heated at 60 °C for 6 h. Cooled to rt and poured onto ice. The mixture was neutralized with 880 ammonia (200 mL). The solid was filtered off, washed with water (50 mL) and air-dried then used directly in the next step. A sample was purified by column chromatography for analysis (SiO_2_, cyclohexane: EtOAc).

^1^H NMR (500 MHz, CDCl_3_) δ 9.22 (dd, *J* = 4.3, 1.7 Hz, 1H), 8.83 (d, *J* = 2.4 Hz, 1H), 8.77 (d, *J* = 2.4 Hz, 1H), 8.39 (dd, *J* = 8.3, 1.7 Hz, 1H), 7.66 (dd, *J* = 8.3, 4.2 Hz, 1H).

^13^C NMR (126 MHz, CDCl_3_) δ 154.68, 147.58, 145.26, 138.78, 127.85, 126.81, 126.68, 124.24, 123.83.

HRMS Calc. for C_28_H_34_N_10_O_5_S_3_ [M + H]^+^ 687.19485 found 687.19444.

To the 8-bromo-6-nitroquinoline (11.1 g, 0.044 mol, 1.0 equiv) in ethanol (112 mL) was added the ammonium chloride dissolved in water (56.6 mL), and iron powder (12.2 g, 0.22 mol, 5.0 equiv) and the reaction gently refluxed (85 °C) overnight. The hot reaction mixture was filtered through Celite, washed with hot methanol (100 mL). The volatiles were removed on a rotary evaporator, and the residue taken up in EtOAc (200 mL) and water (50 mL). The layers were separated then the EtOAc layer washed with brine (50 mL) and dried (MgSO_4_). Yellow brown solid (9.6 g, 0.043 mol, 98.0%) was used crude in the next stage. Using the method reported for Boc formation, a solution of 8-bromoquinolin-6-amine R1 (9.82 g, 0.044 mol, 1.0 equiv) in tert-butanol (9.0 mL) was added the di*tert*-butyl dicarbonate (10.56 g, 0.048 mol, 1.1 equiv) and the mixture was stirred at 60 °C for 16 h. The volatiles were removed on a rotary evaporator and the crude product taken up in EtOAc (100 mL) and imidazole^57^ (1.49 g, 0.022 mol, 0.5 equiv) added and the mixture stirred for 30 min then washed with 1% aq. HCl (2 × 20 mL), NaHCO_3_ (20 mL) and dried (Na_2_SO_4_). Column chromatography using SiO_2_ EtOAc:Pet. ether gave the product **24** (12.72 g, 0.039 mol, 89.4%, Yield over 3 steps).

^1^H NMR (500 MHz, CDCl_3_) δ 8.92 (dd, *J* = 4.3, 1.6 Hz, 1H), 8.08 (dd, *J* = 8.3, 1.7 Hz, 1H), 8.02 (s, 1H), 7.96 (d, *J* = 2.4 Hz, 1H), 7.41 (dd, *J* = 8.3, 4.2 Hz, 1H), 6.85 (s, 1H), 1.54 (s, 9H).

^13^C NMR (126 MHz, CDCl_3_) δ 171.36, 152.61, 149.61, 141.81, 136.95, 136.56, 130.00, 126.24, 124.81, 122.46, 113.83, 81.61, 28.43.

#### Sodium 6-((tert-Butoxycarbonyl)­amino)­quinoline-8-sulfinate (**25**)

Using the reported three step sequence to generate the sulfenic acid salt.^36^

##### Methyl 3-((6-((tert-Butoxycarbonyl)­amino)­quinolin-8-yl)­thio)­propanoate

To the bromide ((2.0 g, 6.2 mmol, 1.0 eq in toluene (20 mL) was added the xantphos (0.179 g, 0.309 mmol, 0.050 equiv) and the Pd_2_(dba)_3_ (0.283 g, 0.309 mmol, 0.05 equiv) and the DIPEA (2.2 mL, 12.4 mmol, 2.0 equiv) The mixture was purged with nitrogen for 10 min then the methyl 3-mercaptopropanoate ((0.7 mL, 6.2 mmol, 1.0 equiv) added and the reaction stirred for 4 h at 100 °C. The reaction mixture was filtered through Celite and then column chromatography, SiO_2_ EtOAc - Pet ether) gave give pure product. Used directly in the next stage.

##### Methyl 3-((6-((tert-Butoxycarbonyl)­amino)­quinolin-8-yl)­sulfonyl)­propanoate

To the sulfide (8.97 g, 24.8 mmol, 1.0 equiv) in acetonitrile (87 mL) was added the pentapotassium dioxidanesulfonoperoxoate hydrogen sulfate sulfate (30.4 g, 49.5 mmol, 2.0 equiv) dissolved in water (116 mL) and the reaction stirred overnight. LCMS indicated complete conversion. Water (60 mL) and EtOAc (200 mL) were added, and the layers were separated. The aqueous layer was extracted with EtOAc (2 × 100 mL). The combined organic layers were washed with brine (1 × 50 mL), and dried (Na_2_SO_4_), and concentrated on the rotary evaporator to afford the product **25** as a white solid. Yield (7.3 g, 18.5 mmol, 75%).

^1^H NMR (600 MHz, DMSO) δ 9.79 (s, 1H), 8.74 (dd, *J* = 4.1, 1.8 Hz, 1H), 8.19 (dd, *J* = 8.4, 1.8 Hz, 1H), 8.05 (d, *J* = 2.5 Hz, 1H), 8.01 (s, 1H), 7.40 (dd, *J* = 8.3, 4.1 Hz, 1H), 1.50 (s, 9H).

^13^C NMR (151 MHz, DMSO) δ 156.63, 153.02, 147.66, 141.73, 137.36, 135.33, 128.73, 121.12, 117.71, 113.17, 79.18, 28.16.

HRMS Calc. for C_14_H_17_N_2_O_4_^32^S [M + H]^+^ 309.09035 found 309.0981.

#### Methyl 3-[[6-(tert-Butoxycarbonylamino)-8-quinolyl]­sulfonylamino] thiophene-2-carboxylate (**26**)

Methanol was used rather than ethanol due to poor solubility of the sulfinate salt in ethanol.^37^ To the sulfenate salt (0.86 g, 5.0 mmol, 2 equiv) in methanol (10.0 mL) was added the iodine R3 (3.46 g, 14 mmol, 1.0 equiv) followed by the amine (1.57 g, 1.0 mmol, 1.0 equiv) in methanol (20 mL). The reaction was stirred for 3 h then 10% sodium thiosulfate solution (20 mL) added, and the methanol removed on the rotary evaporator. EtOAc (100 mL) was added, and the mixture separated, the EtOAc layer was washed with brine (20 mL) and dried (Na_2_SO_4_). Purification, by chromatography (SiO2) gave product (0.240 g, 1 mmol, 19%).

^1^H NMR (600 MHz, Chloroform-*d*) δ 10.65 (s, 1H), 8.93 (dd, *J* = 4.2, 1.7 Hz, 1H), 8.39 (s, 1H), 8.23 (d, *J* = 2.4 Hz, 1H), 8.09 (dd, *J* = 8.4, 1.7 Hz, 1H), 7.47 – 7.41 (m, 2H), 7.26 (d, *J* = 5.5 Hz, 1H), 7.16 (s, 1H), 3.81 (s, 3H), 1.53 (s, 9H).

^13^C NMR (151 MHz, Chloroform-*d*) δ 163.28, 152.60, 149.67, 142.77, 139.58, 136.39, 136.09, 135.58, 131.43, 129.78, 124.43, 124.43, 122.75, 120.55, 119.21, 111.13, 81.71, 51.95, 28.30.

HRMS Calc. for C_20_H_22_N_3_O_6_S_6_ [M + H]^+^ 464.09445 found 464.0934.

#### *tert*-Butyl 4-(4-(((8-(N-(2-(Methoxycarbonyl)­thiophen-3-yl)­sulfamoyl)­quinolin-6-yl)­amino)­methyl)­thiazol-2-yl)­piperazine-1-carboxylate (**27**)

8-(N-(2-(methoxycarbonyl)­thiophen-3-yl)­sulfamoyl)­quinolin-6-aminium 2,2,2-trifluoroacetate. To the Boc protected amine (53.0 mg, 0.12 mmol, 1.0 equiv) compound in DCM (5.0 mL) was added the TFA (0.25 mL, 3.3 mmol) and the reaction stirred overnight. The volatiles were removed on the rotary evaporator and the residue purified by Column Chromatography C18 ACN/H_2_O 0.1% TFA to give the product (31.0 mg, 0.065 mmol, 57%). The product was used directly in the next step.

*tert*-butyl 4-(4-(((8-(N-(2-(methoxycarbonyl)­thiophen-3-yl)­sulfamoyl)­quinolin-6-yl)­amino) methyl)­thiazol-2-yl)­piperazine-1-carboxylate. The method described for compound **11h** was used. On the thiazole quinoline amine (58.0 mg, 0.12 mmol, 1.0 equiv). Yield (40.4 mg, 0.063 mmol, 52%, 29% over two steps).

1H NMR (600 MHz, Chloroform-d) δ 10.62 (s, 1H), 8.74 (dd, *J* = 4.2, 1.6 Hz, 1H), 7.94 (d, *J* = 2.6 Hz, 1H), 7.90 (dd, *J* = 8.4, 1.7 Hz, 1H), 7.47 (d, *J* = 5.5 Hz, 1H), 7.31 (dd, *J* = 8.4, 4.2 Hz, 1H), 7.27 (d, *J* = 5.5 Hz, 1H), 6.91 (d, *J* = 2.7 Hz, 1H), 6.48 (s, 1H), 4.36 (s, 2H), 3.81 (s, 3H), 3.61–3.56 (m, 4H), 3.54 (s, 5H), 1.47 (s, 9H).

^13^C NMR (151 MHz, DMSO) δ 170.92, 162.91, 153.86, 150.01, 145.52, 145.21, 136.47, 134.40, 132.48, 130.74, 123.16, 122.21, 120.66, 107.63, 105.83, 103.59, 79.30, 51.64, 47.70, 43.57, 28.06.

#### Methyl, N2-(3-((6-(((2-(4-(tert-Butoxycarbonyl)­piperazin-1-yl)­thiazol-4-yl)­methyl)­amino)­quinoline)-8-sulfonamido)­thiophene-2-carbonyl)-Nw-((2,2,4,6,7-pentamethyl-2,3-dihydrobenzofuran-5-yl)­sulfonyl)­arginine (**28**)

Using the general method, the methyl ester (97.0 mg, 150.4 μmol, 1.0 equiv) was hydrolyzed. The product was used directly in the next step. The acid (93.4 mg, 148.0 μmol, 1.0 equiv) dissolved in DMF and DIPEA (0.129 mL, 740.0 μmol, 5.0 equiv) and HATU (84.4 mg, 222.0 μmol, 1.5 equiv) were added and the solution stirred at ambient temperature for 10 min before adding H-l-Arg­(Pbf)-OMe (105.9 mg, 222.0 μmol, 1.5 equiv) in one portion. The reaction was stirred at ambient temperature for 18 h after which time LCMS indicated the reaction had gone to completion. Water (0.5 mL) and 3 equiv of AcOH were added and the mixture directly applied to the column Col ACN/H_2_O 0.1% TFA. Yield (18.0 mg, 17.089 μmol, 12%).

#### (2*S*)-5-Guanidino-2-[[3-[[6-[(2-piperazin-1-ylthiazol-4-yl)­methylamino]-8-quinolyl]­sulfonylamino]­thiophene-2-carbonyl]­amino]­pentanoic Acid, Formic Acid, **12h**

This protected arginine mimetic (1 equiv) was dissolved in THF/H_2_O (4:1, 0.04 M) and LiOH (1M, aq., 5.0 equiv) added in one portion. The reaction was stirred at rt until LCMS indicated the reaction had gone to completion. The reaction was then concentrated *in vacuo* and the residue taken up in TFA/DCM (1:1, excess) and stirred at rt until LCMS indicated the reaction had gone to completion. The TFA was removed *in vacuo* to provide the crude product, as a viscous oil. Purification by preparative HPLC (pH9) afforded the title compound (74 mg, 23%, cream solid). Data identical to above.

### SPR Analysis of Compounds

#### Materials

Surface Plasmon Resonance experiments were performed using a Biacore 4000 instrument at a constant temperature of 25 °C. Sensor chips, buffer stock solutions, and immobilization reagents were purchased from GE Healthcare. Recombinant human NRP1-b1 was produced in-house and recombinant human NRP1-ECD (extracellular domain) was purchased from ACRO (catalogue number NR1-H5A228). All other reagents were obtained from Sigma.

#### Immobilization

PBS containing 0.05% surfactant P20 was used as the running buffer during immobilization. NRP1-b1 was immobilized onto a CM5 chip using random amine coupling. The four flow cells were treated in the same way to optimize throughput. In summary, spots 1 and 2 were activated with the coupling reagents EDC and NHS for 10 min. NRP1-b1 at a concentration of 20 μg/mL in 10 mM sodium acetate pH 5 was injected onto the surface for 10 and 5 min in spots 1 and 2, respectively to generate surfaces with high and low density. The immobilization levels ranged from 2302 to 1823 RU on spot 1 and from 948 to 1112 RU in spot 2. The unmodified spot 3 was used as a reference.

#### Kinetics and Affinity Measurements

PBS buffer containing 0.05% surfactant P20 and 3% DMSO was used as the running buffer and sample dilution buffer throughout these experiments. Dose–responses were obtained using a 2-fold sample dilution from 16 μM to 31 nM, using an injection time of 60 s. Surface regeneration between injections was not necessary, but a wash step with 1 M NaCl was included after injection of the highest concentration sample for each compound.

#### Data Processing

Binding curves were corrected for variations in DMSO concentration and normalized by molecular weight. Binding results to high and low-density surfaces were processed independently and the average ± SD is presented. *K*_D_s reported are derived from steady-state binding responses and therefore correspond to the equilibrium binding affinity of the compounds.

### Bt-VEGFA Cell-Free Binding Assay

The assay was conducted as previously described.^33^ Briefly, the 96-well plates were precoated with NRP1 protein at 3 μg/mL overnight at 4 °C. On the following day, the plates were treated with blocking buffer (PBS containing 1%1% BSA) and washed three times with wash buffer (PBS containing 0.1% Tween-20). The various concentrations of compounds diluted in PBS containing 1%1% DMSO were added, followed by addition of 0.25 nM of bt-VEGFA_165_. After 2 h of incubation at room temperature, the plates were washed three times with wash buffer. The bound bt-VEGFA_165_ to NRP1 was detected by streptavidin– horseradish peroxidase conjugates and the enzyme substrate, and measured using a Tecan Genius plate reader at 450 nm with a reference wavelength at 595 nm. Nonspecific binding was determined in the absence of NRP1-coated wells of the plates.

### Protein Expression, Purification, and Crystallization

Frozen cell pellets from 2 L *E. coli* Rosetta (DE3) cells were resuspended in 20 mM Tris-HCl pH 7.9, 20 mM imidazole, 250 mM NaCl and lysed with a cell disruptor (Constant Systems). Soluble protein was isolated by centrifugation, 23,000 rpm, 30 min, 4 °C and incubated with 1 mL Ni-NTA resin (Qiagen) for 2 h at 4 °C. Elution was performed with the buffer containing 250 mM imidazole over 6 column volumes (CV). Dialysis against 20 mM Tris-HCl pH 7.9, 20 mM imidazole, 250 mM NaCl, 5 mM DTT and TEV cleavage were performed overnight at room temperature. The dialysate was incubated with 500 μL Ni-NTA resin for 1 h at 4 °C. The supernatant was retained; the resin was washed twice with 3 mL of 20 mM Tris pH 7.9, 20 mM imidazole, 250 mM NaCl and the washes were added to the unbound supernatant. The supernatant containing the cleaved protein was concentrated and loaded onto a Superdex 75 16/10 size exclusion column equilibrated in 25 mM MES pH 6.0, 50 mM NaCl. Fractions containing NRP1 b1 were pooled, concentrated and loaded onto a MonoS 5/50 column equilibrated in 25 mM MES pH 6.0, 50 mM NaCl. Elution was performed with a gradient of 50 to 500 mM NaCl over 30 CV. Pure NRP1 b1 protein was buffer exchanged into 20 mM Tris-HCl pH 7.9, 50 mM NaCl. Ligand **12d** was added to the purified NRP1 b1 protein to a final concentration of 1 mM. The NRP1 b1/ligand mixtures were incubated at 20 °C for 2 h before concentrating using a Vivaspin 500 spin column (5,000 MWCO). One μL + 1 μL drops of the NRP1 b1/reservoir solution mixtures were set up using the hanging drop method of vapor diffusion. The drops were microseeded immediately after set up using NRP1 b1 apo crystals. Crystallization conditions for ligand **12d** were: NRP1 b1 protein concentration 9.8 mg/mL; 0.2 M ammonium chloride; 14% PEG3350. Crystals took approximately 4 days to reach their maximum dimensions.

### X-ray Crystallography Data Collection and Processing

For data collection, crystals were mounted in nylon loops and flash cooled in liquid nitrogen. All data sets were collected using a Rigaku MicroMax-007HF generator equipped with either a Saturn 944 CCD detector or an R-AXIS IV++ image plate. Reflections were indexed, integrated, and scaled using either MOSFLM and SCALA (CCP4) or HKL2000. A previously published structure of human NRP1 b1 (PDB ID: 1KEX) was used as the search model for molecular replacement using PHASER (CCP4). The resulting models were then automatically rebuilt using BUCCANEER (CCP4) and refined using REFMAC5 (CCP4), with geometric weights automatically assigned. The resulting electron density maps were then examined, and protein residues that showed poor fit in the electron density were adjusted using COOT. Difference electron density maps calculated after initial refinement were examined for the presence of possible ligand. Once electron density corresponding to ligand was located, molecular structure files and refinement library files were produced using JLIGAND (CCP4). The ligands were fitted into the electron density using COOT and refined using REFMAC5. Water molecules were added using the water placement option in COOT and refined using REFMAC5. The structural geometry of both the protein and ligand were finally checked using MOE (Chemical Computing Group).

For details see Supplementary Table S2. The final structure was deposited with the protein data bank PDB ID: 9F6B.

### Retinal Immunostaining

Retinae from C57Bl/6J mice were fixed in 4% formaldehyde in PBS for 1 h and washed three times with PBS. After 1 h in blocking buffer (3% Triton X-100, 1%1% Tween and 0.5% BSA in 2× PBS), retinae were immunostained by incubation with rabbit anti-P-p38 MAPK (Thr180/Tyr182) antibody (Cell Signaling) and biotin-conjugated Isolectin B4 (IB4, Merck) overnight at 4 °C, followed by Alexa Fluor 555–conjugated goat antirabbit antibodies (Invitrogen) and Alexa Fluor 488-conjugated streptavidin (Invitrogen) respectively for 1 h at rt. Images were acquired with a Zeiss Axioskop 2 microscope, using a ph3 Plan-Apochromat 63×/1.40 oil objective, Hamamatsu camera and the HCImage software. Each image was acquired as an RGB color image and then processed with ImageJ software version 1.52a (NIH Bethesda). Channels were split to separate the IB4 staining (green channel) from the P-p38 staining (red channel). The threshold function was used on the IB4 image to determine the area of the vessels and to generate a mask that was restored in the red channel image to measure the area of the vessels positive to P-p38 staining. The P-p38 staining area was normalized against the area stained with IB4. Three retinae from three different mice per condition were used for analysis. One-way ANOVA was used to compare the different treatments.

### Immunoblotting

Cell lysates of the human brain endothelial cell line hCMEC/D3 were prepared in RIPA buffer containing 0.1% SDS, protease inhibitor cocktail 2 and phosphatase inhibitor cocktail (Sigma-Aldrich). Proteins were separated by SDS-PAGE and transferred to nitrocellulose by wet electrotransfer. Membranes were blocked with 5% milk in Tris-buffered saline (TBS) overnight at 4 °C and then incubated with the appropriate primary antibody diluted in TBS containing 0.1% Tween-20 and 1% BSA for 2 h and 30 min at rt; the primary antibodies used were specific for phospho P-p38, p38 (1:1000; Cell Signaling Technology) and GADPH (1:10000; Merck). Membranes were washed with 0.1% Tween-20 in TBS and then incubated with goat antimouse or goat antirabbit horseradish peroxidase (HRP)-conjugated IgG (GE Healthcare) diluted 1:10,000 or 1:5000, respectively, with 0.1% Tween-20, 1% BSA in PBS. Membranes were developed using the ECL reagents (Roche) and images were acquired with the Bio-Rad ChemiDoc MP Imaging System and the Bio-Rad Image Lab software (version 6.0.1). Protein bands were quantified using ImageJ software version 1.52a (NIH Bethesda), whereby signal intensity was normalized to signal intensity from GADPH from the same sample as a loading control. Phosphorylation levels were normalized against the total levels of p38. Densitometric quantification of three independent immunoblots was determined by changes in protein or phosphoprotein content normalized to GADPH total protein loading controls, with values expressed as fold increase. Data are shown as mean ± SD. Statistical analysis included one-way ANOVA with significance levels set at 0.05, followed by posthoc Dunnett’s tests.

### Ex Vivo Retina Permeability Assay

C57Bl/6J mice were culled through CO_2_ overdose before proceeding with the cannulation of the common carotid arteries and perfusion of the vasculature, as previously described.^30^ Each eye was subsequently removed and enucleated. The retina was isolated with the attached sclera, flattened onto a silicone base (SYLGARD 184, Merck), and held in position by a metal ring and pins. Retinal explants were visualized with an Olympus 10× objective on an upright Axiophot fluorescence microscope (Zeiss) and continuously superfused with Krebs solution (124 mM NaCl, 5 mM KCl, 2 mM MgSO_4_, 22 mM Na_2_CO_3_, 0.125 mM NaH_2_PO_4_ and 2 mM CaCl_2_, pH 7.4) supplemented with 5 mM glucose and 0.1% BSA w/v. A radial vein was injected with 1 mg/mL sulforhodamine B (479 Da; Merck) in Krebs solution using a glass needle to visualize the vasculature under a TRITC filter with an Olympus 40× water immersion objective. For permeability measurements, the fluorescence of a selected microvessel was recorded continuously by time-lapse imaging with a CCD camera (Hamamatsu) and HCImageLive software (Hamamatsu) for at least 30 s to obtain a baseline. VEGFA_164_ or EG00229 in Krebs solution were then added dropwise onto the retina. Recording continued for a minimum of 90 s. Time-lapse series were analyzed using ImageJ (NIH Bethesda). Pixel intensity measurements were collected and plotted against time.^30^ Permeability measurements from at least three different ex vivo retinal preparations were combined and expressed as mean ± SD. Repeated-measure one-way ANOVA was utilized to compare the baseline and VEGFA_164_-induced permeability with and without pharmacological inhibitors.

### Pharmacokinetics

To test compound drug-like properties, selected compounds with low IC_50_ were further evaluated for their pharmacokinetic (PK) profile. 6–8 week-old BABL/c female mice were used. Two mg/kg of compounds was formulated in 7.5% DMSO and 92.5% PBS solution and intravenously dosed into the tail vein as a bolus. Blood samples were collected by cardiac puncture at 5, 15, 30, 60, 180, and 240 min post dosing. Plasma samples were prepared by centrifugation at 7000 rpm for 5 min, and supernatants were collected, immediately snap-frozen on dry ice and stored at −20 °C. Samples were analyzed by liquid chromatography-tandem mass spectrometry using electrospray ionization and data was analyzed by WinNonlin software.

### Model Studies on Nociception

#### Animals

Pathogen-free rats were kept in light (12-h light: 12-h dark cycle; lights on at 07:00 h) and temperature (23 ± 3 °C) controlled rooms. Female Sprague–Dawley rats (∼75–100 g, Charles River Laboratories, Wilmington, MA.) were employed for DRG electrophysiological recordings. For behavioral experiments, male and female rats were 6 weeks old upon arrival and were left to acclimatize to the surroundings for at least 1 week before the start of behavioral experiments. Standard rodent chow and water were available *ad libitum.* All animal use was conducted in accordance with the National Institutes of Health guidelines, and the study was conducted in strict accordance with recommendations in the Guide for the Care and Use of Laboratory Animals of the College of Dentistry of the New York University. All efforts were made to minimize animal suffering. All behavioral experiments were performed by the same experienced female experimenter, who was blinded to the treatment.

#### Dorsal Root Ganglion Neuron Cultures

Lumbar DRGs were dissected from 75 to 100 g female Sprague–Dawley rats. DRGs were excised and placed in sterile DMEM (Cat# 11965; Thermo Fisher Scientific, Waltham, MA). The ganglia were dissociated enzymatically with collagenase type I (1.66 mg/mL, Cat# LS004194; Worthington) and neutral protease (1.04 mg/mL, Cat# LS02104; Worthington, Lakewood, NJ) for 50 min at 37 °C under gentle agitation. The dissociated cells were then centrifuged (800 rpm for 5 min) and resuspended in DMEM containing 1% penicillin/streptomycin sulfate (Cat# 15140, Life Technologies, Carlsbad, CA) and 10% fetal bovine serum [HyClone]). The cells were seeded on poly-d-lysine (0.1 mg/mL; Cat# P6407, Millipore Sigma, St. Louis, MO) and laminin (1 mg/mL; Cat#sc-29012, Santa Cruz Biotechnology, Dallas, TX) -coated 12 mm glass coverslips and incubated at 37 °C. All cultures were used within 48 h.

#### Whole-Cell Patch-Clamp Recordings of Na^+^ Currents in Acutely Dissociated DRG Neurons

Recordings were obtained from acutely dissociated DRG neurons obtained from female rats as described earlier. Patch-clamp recordings were performed at room temperature (22–24 °C). Currents were recorded using an EPC 10 Amplifier-HEKA (HEKA Elektronik, Ludwigshafen, Germany) linked to a computer with Patchmaster software.

To determine the effect of VEGFA application on voltage-gated sodium currents, we incubated DRG neurons for 30 min with recombinant rat VEGFA, 1 nM (Cat#P4853, Abnova, Taipei, Taiwan) before whole-cell patch-clamp recordings. Additionally, 30 μM EG00229 (Cat#6986, Tocris Bioscience, Bristol UK) and 30 μM **12h** (in DMSO) were also applied to the culture medium for 30 min before recording. For experiments where VEGFA was tested in combination with the compounds, EG00229 and **12h** were added first for 30 min, followed by VEGFA for another 30 min before recording sodium currents. For the control condition, DRG neurons were incubated for 30 min with DMSO at a final concentration of 0.1%. VEGFA, EG00229 and **12h** were added at the same concentrations in the external recording solution during all data acquisition.

For Na^+^ current (*I*_Na+_) recordings, the external solution contained (in mM): 130 NaCl, 3 KCl, 30 tetraethylammonium chloride, 1 CaCl_2_, 0.5 CdCl_2_, 1 MgCl_2_, 10 d-glucose and 10 HEPES (pH 7.3 adjusted with NaOH, and mOsm/L = 315). Patch pipettes were filled with an internal solution containing (in mM): 140 CsF, 1.1Cs-EGTA, 10 NaCl, and 15 HEPES (pH 7.3 adjusted with CsOH, and mOsm/L = 300). Peak Na^+^ current was acquired by applying 150 ms voltage steps from – 70 to +60 mV in 5-mV increments from a holding potential of – 60 mV to obtain the current–voltage (I–V) relation.

Normalization of currents to each cell’s capacitance (pF) was performed to allow for collection of current density data. For I–V curves, functions were fitted to data using a nonlinear least-squares analysis. I–V curves were fitted using double Boltzmann functions:

$$f=a+g1/(1+\exp((x-V_{1/2}1)/k1))+g2/(1+\exp(-(x-V_{1/2}2)/k2))$$

where *x* is the membrane potential, *V*_1/2_ is the midpoint potential and *k* is the corresponding slope factor for single Boltzmann functions. Double Boltzmann fits were used to describe the shape of the curve, not to imply the existence of separate channel populations. Numbers 1 and 2 simply indicate first and second midpoints; *a* along with *g* are fitting parameters.

Activation curves were obtained from the I–V curves by dividing the peak current at each depolarizing step by the driving force according to the equation: *G* = *I*/(*V*_mem_ – *E*_rev_), where *I* is the peak current, *V*_mem_ is the membrane potential and *E*_rev_ is the reversal potential. The conductance (*G*) was normalized against the maximum conductance (*G*_max_). Steady-state inactivation (SSI) curves were obtained by applying an H-infinity protocol that consisted of 1-s conditioning prepulses from −120 to +10 mV in 10-mV increments followed by a 200 ms test pulse to +10 mV. Inactivation curves were obtained by dividing the peak current recorded at the test pulse by the maximum current (*I*_max_). Activation and SSI curves were fitted with the Boltzmann equation.

### Behavioral Experiments

#### Hind Paw Injection Procedure

The procedure was performed as reported previously.^5^ Briefly, rats were gently restrained in a fabric cloth and given an intraplantar injection in the hind-paw containing VEGFA_165_ (10 nM) and compound (EG00229 or **12h**, at 30 or 10 μM) alone or in combination in 50 μL of PBS vehicle (NaCl 137 mM, KCl 2.5 mM, Na_2_HPO_4_ 10 mM, and KH_2_PO_4_ 1.8 mM), using a 31G needle.

#### Mechanical Allodynia, VF

Low intensity mechanical sensitivity was assessed by using a series of calibrated von Frey monofilaments (North Coast Medical, Inc., Morgan Hill), similar to previous studies.^52^ Animals were placed in individual Plexiglas (9.5 × 14 × 19.0 cm) enclosures on an elevated wire grid. They were given approximately 15 min to acclimate to the enclosure and the experimenter’s presence and movements below the grid, prior to stimulation of the plantar surface of the hind paw with a series of calibrated von Frey filaments (0.4, 0.6, 1.0, 1.4, 2.0, 4.0, 6.0, 8.0, 10.0, 15.0, 26.0 g). To initiate testing a filament with a bending force of 4.0 g was first applied to the hind paw with uniform pressure for 5 s. A brisk withdrawal was considered a positive response whereupon the next lower filament in the series was applied. In the absence of a positive response the neighboring higher filament was applied. After the first change in response-pattern, indicating the threshold, 4 additional applications were performed; when there was no response, the next filament with a higher force was tested, and when response was positive, the next lower force filament was tested. The 50% threshold was determined by the following equation: 50% threshold (*g*) = 10^log(last filament)+*k*×0.3^. The constant, *k*, was found in the table by Dixon^58^ and determined by the response-pattern.

#### Cold Allodynia, ADT

Cold allodynia was assessed based on previously published protocols.^52^ While the animals were still in the Plexiglas chambers following von Frey measurements, cold allodynia was assessed using application of a drop of acetone (Acetone Drop Test, ADT) to the plantar surface of the paw, using an 18 gauge plastic feeding-tube connected to a syringe without mechanically touching the skin with the tube. Following application, the duration of the response was then recorded, with a maximum of 60 s. A positive response was considered as flinching, licking or withdrawing the paw. The application and assessment were performed two times per animal with 5–10 min between each application, and the average of the two measurements was calculated.

#### Conditioned Place Aversion (CPA)

The experiments were conducted in a two-chamber device based on the protocols from^53^ and as previously described.^52^ The protocol includes 4 × 10 min of sequential tests of preconditioning (10 min), conditioning (2 × 10 min) and testing (10 min). During preconditioning, the animal is allowed free access to two connected chambers (30 × 30 × 19 cm), each associated with a scented lip-balm applied to the walls. Immediately following preconditioning, a divider was applied between the chambers, and the rats were conditioned to either stimuli or no-stimuli for 10 min in each chamber. The stimuli consisted of repeated stimulation with a 10 g VF-filament every 30 s for the 10 min that the subject was contained in that chamber, while no stimuli (NS) was applied in the other chamber. The order and side of conditioning was alternated between subjects. Following the conditioning, the divider was removed, and the rat was allowed free access to both chambers for the 10 min test. Animal movements in each chamber were recorded by a camera above, and the duration of time spent in each chamber was recorded during preconditioning and test phase. Decreased time spent in a chamber during the test versus preconditioning indicated avoidance for that chamber and was calculated as a CPA-score: time in VF-chamber during preconditioning – time in VF-chamber during test.

## Supplementary Material





## Notes

Vascular endothelial growth factor A; X-ray structure; uncropped Western blots; crystallographic parameters; statistical analysis; and NMR and LCMS traces for the indicated molecules (PDF)
SMILES string computer-readable identifiers for the presented molecules (XLSX)

## ○

Department of Pharmacology and Therapeutics, University of Florida College of Medicine, Gainesville, Florida 32610, United States

## ◆

Department of Veterinary and Animal Sciences, University of Copenhagen, 1870 Frederiksberg, Denmark

## ¶

School of Applied Sciences, Lewes Rd, Moulsecoomb, Brighton BN24GJ, U.K.

## Abbreviations

Bt: biotin
CNS: central nervous system
DRG: dorsal root ganglia
H-bonding: hydrogen bonding
MAPK: mitogen-activated protein kinase
NRP1: neuropilin-1
NSAIDs: nonsteroidal anti-inflammatory drugs
SPR: surface plasmon resonance
TGFβ1: transforming growth factor beta
VEGF: vascular endothelial growth factor
VEGFA: vascular endothelial growth factor A
VEGFR: VEGF receptor
