# Supplementary material for: Quinoline-Based Neuropilin‑1 Antagonists Exhibit a Pure Antagonist Profile and Block Vascular Endothelial Growth Factor-Induced Pain
Source: ACS Pharmacol Transl Sci. 2025 Oct 29;8(11):3844–71. doi: 10.1021/acsptsci.5c00029 (PMC12624434; doi:10.1021/acsptsci.5c00029)
Supplement: Supplementary file 2 [file pt5c00029_si_001.pdf]

Supplementary information

Quinoline-based neuropilin-1 antagonists exhibit a pure antagonist profile and block vascular endothelial growth factor-induced pain

Authors

Sara Hestehave[7,9], Silvia Dragoni[6,10], Philip Fallon[2], Filipa Mota[1], Aida Calderon-Rivera[7,8], Kimberly Gomez[7,8], Jonathan Powell[2], Anastasia Patsiarika[1], Tifelle Reisinger[2], Stuart Crosby[2], A.W. Edith Chan[1], David Steadman[1], Natalie Winfield[2], Ashley Jarvis[2], John Martin[3], Ian C. Zachary[3], Paul Frankel[5], Snezana Djordjevic[4], Christiana Ruhrberg[6], Rajesh Khanna[7,8], David L. Selwood[1]*

1The Wolfson Institute for Biomedical Research, University College London, Gower Street, London WC1E 6BT, UK.

2NCE Discovery (Domainex Ltd), Chesterford Research Park, Little Chesterford, Saffron Walden, Essex CB10 1XL, UK.

3Centre for Cardiovascular Biology and Medicine, Division of Medicine, University College London, 5 University Street, London, WC1E 6JJ, UK.

4Institute of Structural and Molecular Biology, University College London, Gower Street, London WC1E 6BT, UK.

5Institute of Cardiovascular Science, University College London, 5 University Street, London WC1E 6JF, UK.

6UCL Institute of Ophthalmology, University College London, 11-43 Bath Street, London EC1V 9EL, UK.

7Department of Molecular Pathobiology, College of Dentistry, New York University, New York, NY, 10010, USA.

8Current address: Department of Pharmacology and Therapeutics, University of Florida College of Medicine, Gainesville, Florida, 32610, USA.

9Current address: Department of Experimental Medicine, University of Copenhagen, 2200 Copenhagen N, Denmark.

10Current address: School of Applied Sciences, Lewes Rd, Moulsecoomb, Brighton BN24GJ, UK.

Table of Contents

Supplementary Figures ... 3

Figure S1. X-ray structure of 12d ... 3

Figure S2. Uncropped Western Blots for Figure 4C. ... 4

Figure S3. Vascular endothelial growth factor A (VEGFA) induces a pain-like phenotype that is blocked by 12h in male and female rats. ... 5

Figure S4. Vascular endothelial growth factor A (VEGFA) induces an increased aversion to mechanical stimuli in male and female rats. ... 6

Figure S5. Vascular endothelial growth factor A (VEGFA) induces an increased aversion to mechanical stimuli in male and female rats, that is blocked by 10 μM 12h in males. ... 7

Supplementary tables ... 8

Table S1. 2,1,3-Benzothiadiazoles in the protein databank[a] ... 8

Table S2. Crystallographic parameters ... 9

Table S3 . Statistical analysis, EG00229 vs 12h ... 10

Table S4 Statistical analysis of data in supplementary figures ... 11

NMR spectra and LC traces. ... 14

8-Bromo-6-nitroquinoline ... 14

8-bromoquinolin-6-amine ... 14

tert-butyl (8-bromoquinolin-6-yl)carbamate ... 15

sodium 6-((tert-butoxycarbonyl)amino)quinoline-8-sulfinate ... 17

methyl 3-[[6-(tert-butoxycarbonylamino)-8-quinolyl]sulfonylamino]thiophene-2-carboxylate ... 20

8-(N-(2-(methoxycarbonyl)thiophen-3-yl)sulfamoyl)quinolin-6-aminium 2,2,2-trifluoroacetate ... 22

tert-butyl 4-(4-(((8-(N-(2-(methoxycarbonyl)thiophen-3-yl)sulfamoyl)quinolin-6-yl)amino)methyl)thiazol-2-yl)piperazine-1-carboxylate ... 23

Methyl, N2-(3-((6-(((2-(4-(tert-butoxycarbonyl)piperazin-1-yl)thiazol-4-yl)methyl)amino)quinoline)-8-sulfonamido)thiophene-2-carbonyl)-Nw-((2,2,4,6,7-pentamethyl-2,3-dihydrobenzofuran-5-yl)sulfonyl)arginine ... 25

(2*S*)-5-guanidino-2-[[3-[[6-[(2-piperazin-1-ylthiazol-4-yl)methylamino]-8-quinolyl]sulfonylamino]thiophene-2-carbonyl]amino]pentanoic acid, formic acid 12h ... 26

Supplementary Figures

Figure S1. X-ray structure of **12d**

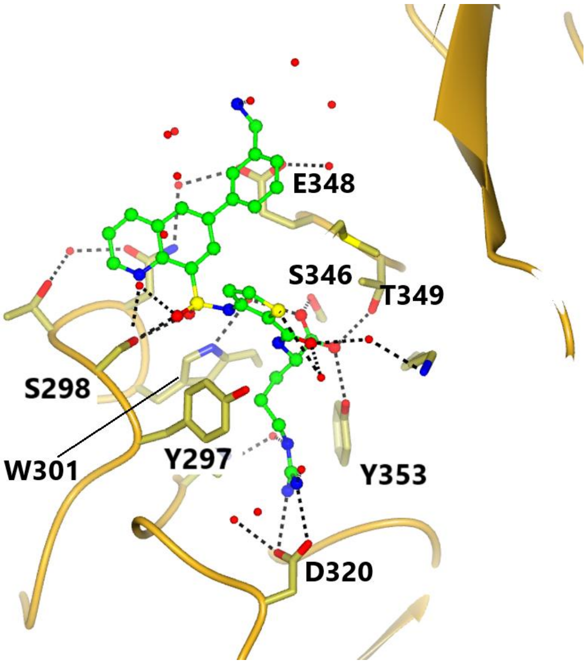

Figure S1. X-ray crystal structure of **12d** (Pdb: 9F6B). Compound **12d** (green, ball-and-stick representation) makes extensive H-bond contacts with NRP1 residues and with bound water molecules. **12d** bound to the B chain. The H-bonds from the quinoline nitrogen to S298 are visible in both chains within the asymmetric unit,

Figure S2. Uncropped Western Blots for Figure 4C.

Supplementary information – Full uncropped blots for Figure 4.

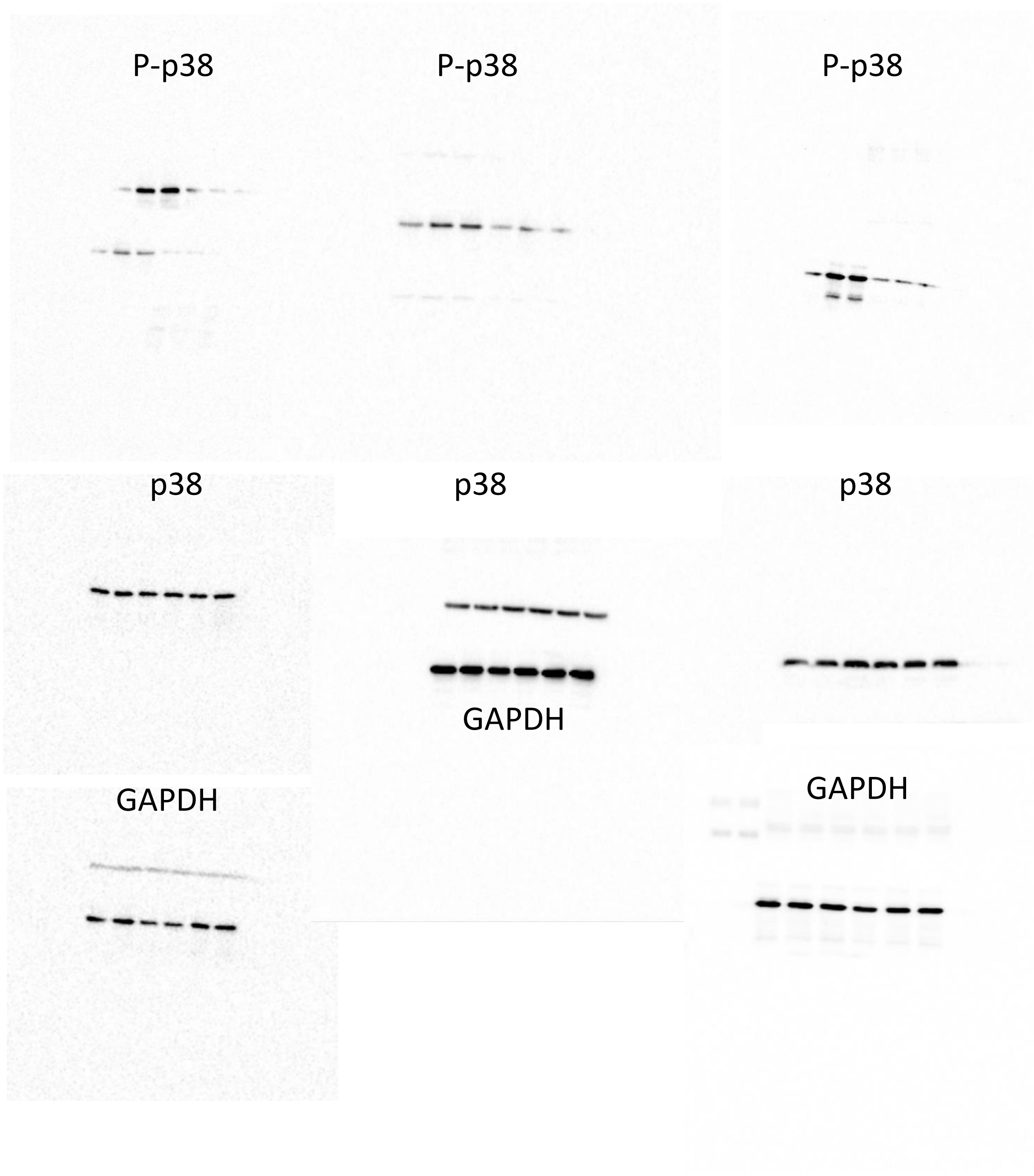

Figure S3. Vascular endothelial growth factor A (VEGFA) induces a pain-like phenotype that is blocked by **12h** in male and female rats.

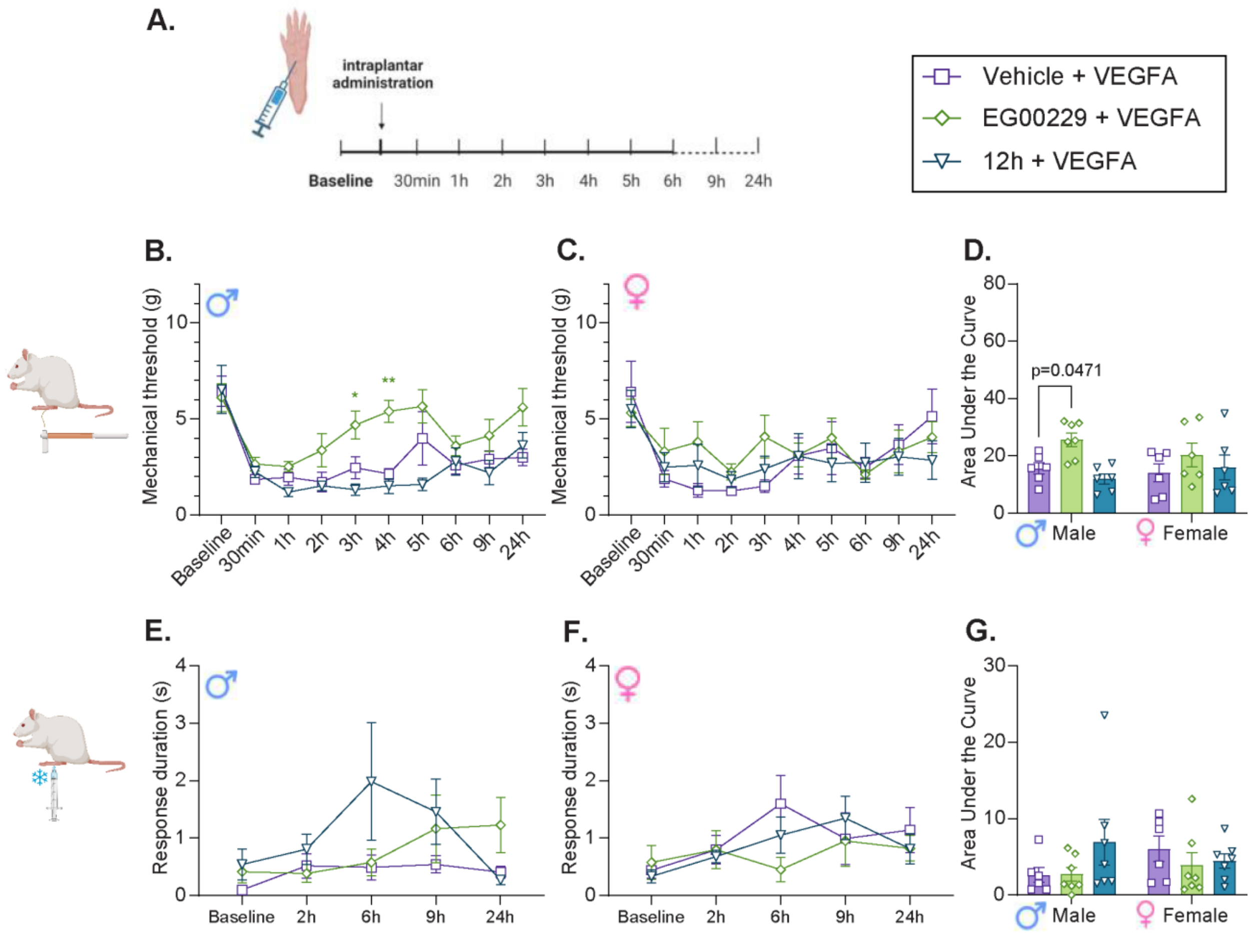

Figure S3. Vascular endothelial growth factor-A (VEGF-A) induces a pain-like phenotype that is blocked by 12h in male and female rats.

Study-design schematic and treatment conditions. Naïve rats of both sexes were given an intraplantar injection with $VEGFA_{165}$ (10 nM) in combination with either vehicle (PBS) or one of the two NRP1 inhibitors, EG00229 or **12h** (10 µM) in a volume of 50 µl/rat. Mechanical allodynia was assessed using paw withdrawal thresholds to mechanical stimuli (von Frey filaments, vF) in both male (B) and female (C) rats. (D) Quantification of Area Under the Curve (AUC) of the paw withdrawal threshold reported in panel B-C from Baseline to 6 h post administration. Cold allodynia was assessed by recording the response duration to the application of an acetone drop (Acetone Drop Test, ADT) in male (E) and female (F) rats. (G) Quantification of Area Under the Curve of the response duration to ADT from baseline to 6 hours after injection. Error-bars indicate mean ± SEM. Sex of the animals is labelled as ♂:male and ♀:female. Time-course data were analyzed using two-way RM ANOVA, treatment*time, and Tukey's post hoc test, with difference from vehicle indicated by *$P<0.05$, **$P<0.01$, ***$P<0.001$. AUC data was analyzed using two-way ANOVA, treatment*sex, and Dunnett's post hoc test suggesting differences from the sex-specific vehicle treatment group. n=6-7. For full statistical analyses, see Table S2.

Figure S4. Vascular endothelial growth factor A (VEGFA) induces an increased aversion to mechanical stimuli in male and female rats.

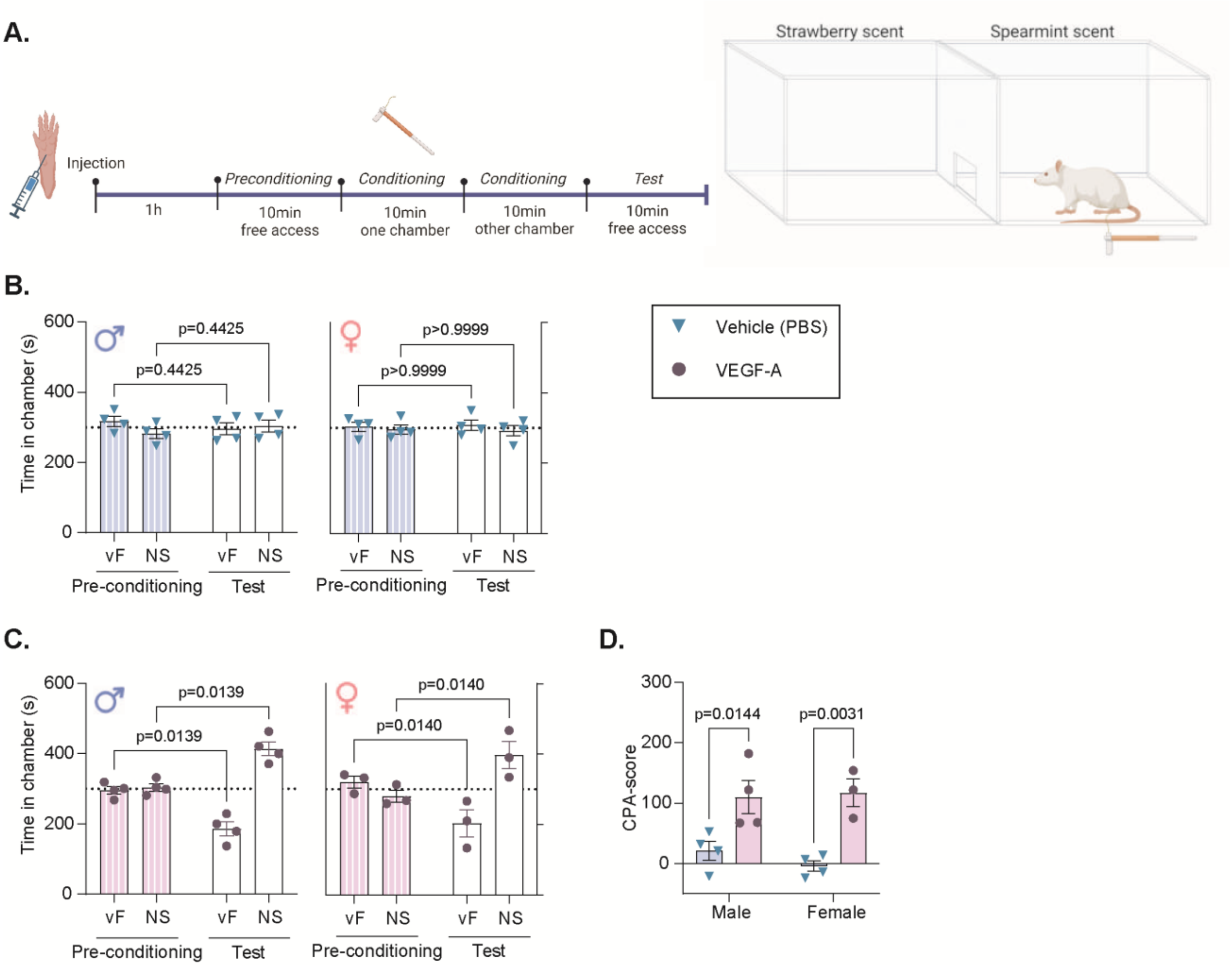

Figure S4. Vascular endothelial growth factor A (VEGFA) induces an increased aversion to mechanical stimuli in male and female rats.

Naïve rats of both genders were injected intraplantar with VEGFA (10nM) or vehicle (PBS), 50µl/rat. **A)** Schematic of the study design. 1 h after injection, the rat was exposed to the 2-chamber CPA-test, including 4*10 min consecutive sessions of: preconditioning, conditioning to each chamber, and test. Conditioning included one chamber conditioned to stimulation with a 10 g vF-filament every 30 seconds in one chamber, and no stimulation (NS) in the other **B)** Animals injected with vehicle alone, did not have any aversion to the chamber conditioned with vF-10g stimulation. **C)** VEGFA treated animals showed increased aversion to the vF-conditioned chamber during the test. **D)** VEGFA induced aversion to stimuli with vF-10 g as seen by increased CPA-scores when compared with vehicle. CPA score = time in VF-chamber during preconditioning – time in VF-chamber during test. Error-bars show mean ± SEM, and sex is indicated by ♂:males and ♀:females. n=3-4. For full statistical analyses, see Table S2.

Figure S5. Vascular endothelial growth factor A (VEGFA) induces an increased aversion to mechanical stimuli in male and female rats, that is blocked by 10 µM 12h in males.

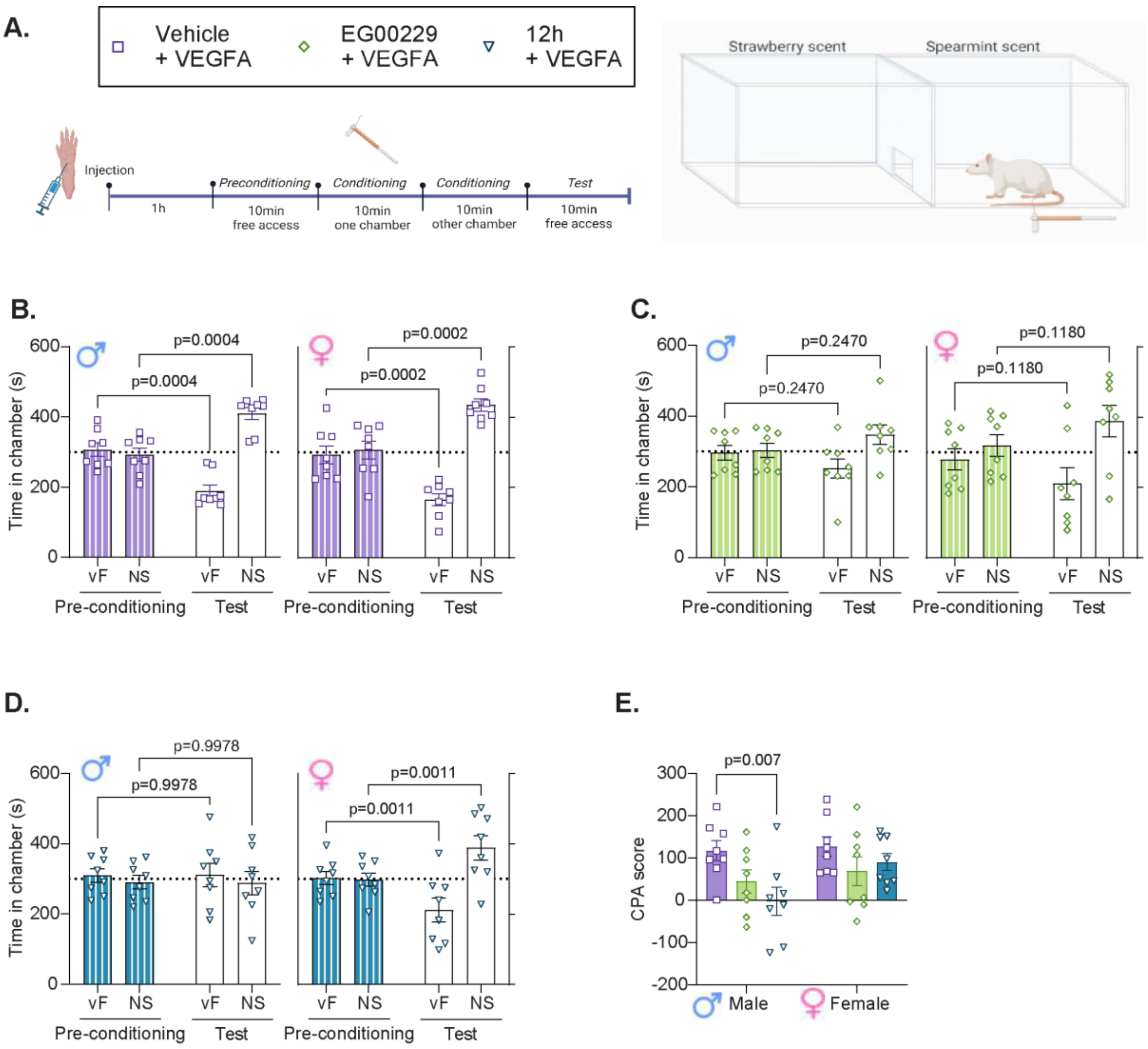

Figure S5. Vascular endothelial growth factor A (VEGFA) induces an increased aversion to mechanical stimuli in male and female rats, that is blocked by 10 µM 12h in males.

Naïve rats of both sexes were injected intraplantar with VEGFA (10 nM) in combination with either vehicle (PBS) or one of the two NRP1 inhibitors, EG00229 or **12h** (10 µM), 50 µl/rat. **A)** Schematic of the study design. 1 h after injection, the rat was exposed to the 2-chamber CPA-test, including 4*10 min consecutive sessions of: preconditioning, conditioning to each chamber, and test. Conditioning included one chamber conditioned to stimulation with a 10 g vF-filament every 30 seconds in one chamber, and no stimulation (NS) in the other. **B)** Vehicle / VEGFA treated animals showed increased aversion to the vF-conditioned chamber during the test for both male (♂) and female (♀) rats. **C)** NRP1 inhibitor EG00229 prevented the stimulus-aversion from VEGFA in both male and female rats. **D)** NRP1 inhibitor **12h** prevented significant stimulus-

aversion from VEGFA in males (♂). **E)** NRP1 inhibitors prevented the aversive effects from VEGFA as demonstrated by decreased CPA-scores when compared with vehicle. CPA score = time in VF-chamber during preconditioning – time in VF-chamber during test. n=8. P-values as suggested by appropriate post hoc test. For full statistical analyses, see Table S2.

Supplementary tables

Table S1. 2,1,3-Benzothiadiazoles in the protein databank[a]

**PDB code**	**H-bond to 1,3 nitrogens**	**Comment**
4DDM	No H bonds to protein	This structure shows Mycobacterium tuberculosis pantothenate synthetase in complex with 2,1,3-benzothiadiazole-5-carboxylic acid. This was part of a fragment-based drug discovery effort.
5UXZ	No H bonds to protein	contain X-ray crystal structures of a Halo-tagged protein bound to a fluorogenic benzothiadiazole ligand. These studies used benzothiadiazoles as fluorophores for protein imaging.
2pj9	No H bonds to protein	CRYSTAL STRUCTURE OF ACTIVATED PORCINE PANCREATIC CARBOXYPEPTIDASE
7HCE	No H bonds to protein, 1 to solvent	Crystal structure of SARS-CoV-2 NSP3 macrodomain in complex with AVI-0000002
3i97	Yes in one chain	B1 domain of human Neuropilin-1 bound with small molecule EG00229

[a] The term 2,1,3-benzothiadiazole was used to search the pdb alongside structure searches.

Table S2. Crystallographic parameters

NRP1 complex with 12d	
**Data collection**	
PDB ID	9F6B
Space group	P2(1)
Unit cell parameters	
Number of protein chains per AU	2
a, b, c (Å)	40.57, 92.22, 40.83
α, β, γ (°)	90.00, 94.84, 90.00
Wavelength (Å)	1.54
Resolution (Å)	20.51-1.57 (1.60-1.57)
Number of unique reflections	41294
Rmerge	4.3 (21.3)
$\langle I/\sigma(I)\rangle$	14.3 (3.2)
Completeness (%)	98.8 (82.1)
Multiplicity	3.1 (1.8)
**Refinement**	
Resolution (Å)	20.51-1.57 (1.61-1.57)
Number of reflections	39098 (2480)
Number of reflections (R-free)	2060 (147)
Rwork/Rfree (%)	19.3/23.9 (23.8/29.2)
r.m.s. deviations	
bond length (Å)	0.024
Bond angles (°)	2.344
Ramachandran plot	
favoured (%)	96
allowed (%)	4
outliers (%)	0
Mean B values ($Å^2$)	
Overall	13.0
Water	23.1
Ligand	12.7
Number of atoms in AU	
Protein	2481
Ligand	82
Water	460

Table S3 . Statistical analysis, EG00229 vs 12h

Figure number	Analysis	Outcome	Post-test
**Fig. 5 VEGFA-mediated increase in total sodium currents is prevented by NRP1 inhibition**			
Fig 5C. Peak Na+ current density	One way ANOVA	$F(5,75)=3.732$, $P=0.0045$	Dunnett
Fig 5D. V1/2 of activation	One way ANOVA	$F(5,82)=7.709$, $P<0.0001$	Dunnett
Fig 5D. $V_{1/2}$ of inactivation	One way ANOVA	$F(5,75)=0.5813$, $P=0.7141$	Dunnett
**Fig 6. VEGF-A induced allodynia is reduced by 12h**			
Fig 6B. VF males – 30µM	Two-way RM ANOVA, time*treatment	$F_{time}(9,135)=7.382$, $P<0.0001$<br>$F_{treatment}(2,15)=6.822$, $P=0.0078$<br>$F_{time*treatment}(18,135)=1.861$, $P=0.0243$	Tukey
Fig 6C. VF females - 30 µM	Two-way RM ANOVA, time*treatment	$F_{time}(9,135)=12.79$, $P<0.0001$<br>$F_{treatment}(2,15)=4.643$, $P=0.0269$<br>$F_{time*treatment}(18,135)=2.012$, $P=0.0129$	Tukey
Fig 6D. VF – AUC - 30 µM	Two-way ANOVA, sex*treatment	$F_{treatment}(2,30)=16.16$, $P<0.0001$	Dunnett
Fig 6E. ADT males – 30µM	Mixed-effects model*, time*treatment	NS	
Fig 6F. ADT females - 30 µM	Mixed-effects model*, time*treatment	$F_{treatment}(2,15)=7.058$, $P=0.0069$	Tukey
Fig 6G. ADT – AUC - 30 µM	Two-way ANOVA, sex*treatment	$F_{treatment}(2,30)=4.125$, $P=0.0261$<br>$F_{sex*treatment}(2,30)=7.200$, $P=0.0028$	Dunnett

**Fig 7.** ***Vascular endothelial growth factorA (VEGFA) induces an increased aversion to mechanical stimuli in male and female rats, that is blocked by 12h.***			
Fig 7B – vehicle treatment, time in chamber	3-way RM ANOVA, time*chamber*sex	$F_{chamber}$ (1,14) = 21.81, P=0.0004<br>$F_{time*chamber}$ (1,5) = 112.3, P<0.0001	Sidak
Fig 7C – EG00229 treatment, time in chamber	3-way RM ANOVA, time*chamber*sex	NS	Sidak
Fig 7D – **12h** treatment, time in chamber	3-way RM ANOVA, time*chamber*sex	NS	Sidak
Fig 7E. CPA-score, NRP-1 inhibitors.	Two Way ANOVA, sex*treatment	$F_{treatment}$ (2,42) = 11.23, P=0.0001	Dunnett

*Mixed-effects model was used due to missing values, as one cohort (including equal numbers from all groups) was not tested in the ADT test at 9 and 24h.

Table S4 Statistical analysis of data in supplementary figures

**Figure number**	**Analysis**	**Outcome**	**Post-test**
**Fig S1.** ***VEGFA induced allodynia is reduced by NRP1 inhibition, 10µM***			
Fig S1B. VF males – 10µM	Two-way RM ANOVA, time*treatment	F_{time}(9,153)=14.73, P<0.0001<br>$F_{treatment}$(2,17)=8.951, P=0.0022<br>$F_{time*treatment}$(18,153)=2.097, P=0.0083	Tukey
Fig S1C. VF females - 10µM	Two-way RM ANOVA, time*treatment	F_{time}(9,135)=9.290, P<0.0001	
Fig S1D. VF – AUC - 10µM	Two-way ANOVA, sex*treatment	$F_{treatment}$(2,31)=5.295, P=0.0105	Dunnett
Fig S1E. ADT males – 10µM	Two-way RM ANOVA, time*treatment	F_{time} (4,68)=2.344, P=0.0634 (NS)	

		$F_{time*treatment}$ (8,68)=1.853, P=0.0822 (NS)	
Fig S1F. ADT females - 10µM	Two-way RM ANOVA, time*treatment	F_{time} (4,68)=3.551, P=0.0109	
Fig S1G. ADT – AUC - 10µM	Two-way ANOVA, sex*treatment	NS	

**Fig S2.** ***Vascular endothelial growth factor-A (VEGF-A) induces an increased aversion to mechanical stimuli male and female rats.***

Fig S2B. Naïve	3-way RM ANOVA, time*chamber*sex	NS	Sidak
Fig S2C. VEGF-A	3-way RM ANOVA, time*chamber*sex	$F_{chamber}$ (1,5)=15.67, P=0.0108<br>$F_{time*chamber}$ (1,5)=36.43, P=0.0018	Sidak
Fig S2D. VEGF-A pilot, CPA-score	Two Way ANOVA, sex*injury	F_{injury} (1,11) = 28.00, P=0.0003	Sidak
Fig S3. Vascular endothelial growth factor-A (VEGF-A) induces an increased aversion to mechanical stimuli in male and female rats, that is blocked by 10µM 12h in males			
Fig S3B – vehicle treatment, time in chamber	3-way RM ANOVA, time*chamber*sex	$F_{chamber}$ (1,14) = 28.56, P=0.0001<br>$F_{time*chamber}$ (1,14) = 53.81, P<0.0001	Sidak

Fig S3C – EG00229 treatment, time in chamber	3-way RM ANOVA, time*chamber*sex	$F_{chamber}$ (1,14) = 4.074, P=0.0631 (NS)<br>$F_{time*chamber}$ (1,14) = 6.704, P=0.0214	Sidak
Fig S3D – **12h** treatment, time in chamber	3-way RM ANOVA, time*chamber*sex	$F_{chamber}$ (1,14), P=0.345 (NS)<br>$F_{time*chamber}$ (1,14) = 5.200, P=0.039<br>$F_{time*chamber*sex}$ (1,14) = 5.679, P=0.032	Sidak

Fig S3E. CPA-score, NRP-1 inhibitors, .	Two Way ANOVA, sex*treatment	$F_{treatment}$ (2,42) = 4.627, P=0.0153<br><br>F_{sex} (1,42) = 3.551, P=0.0664 (NS)	Dunnett

NMR spectra and LC traces.

8-Bromo-6-nitroquinoline

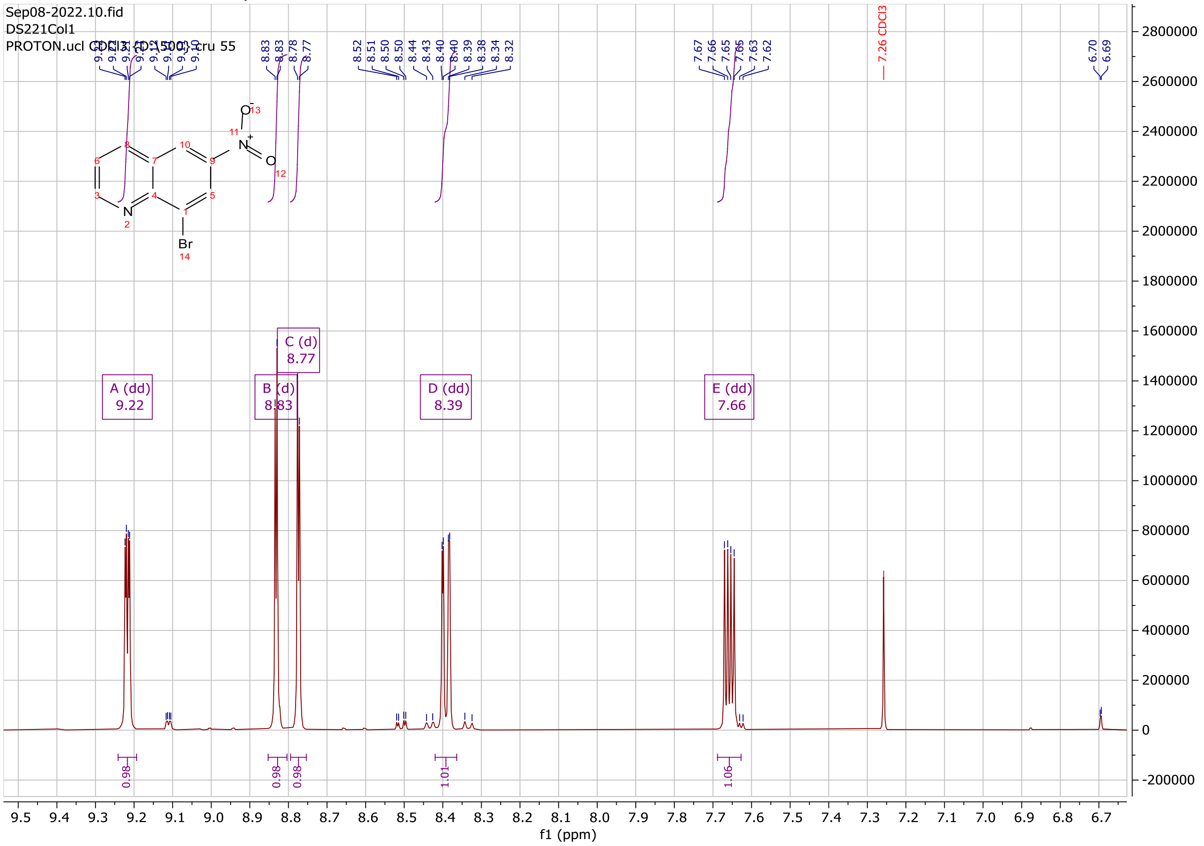

8-bromoquinolin-6-amine

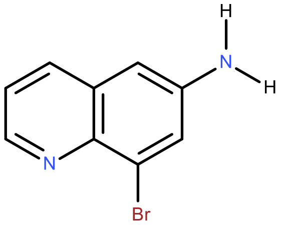

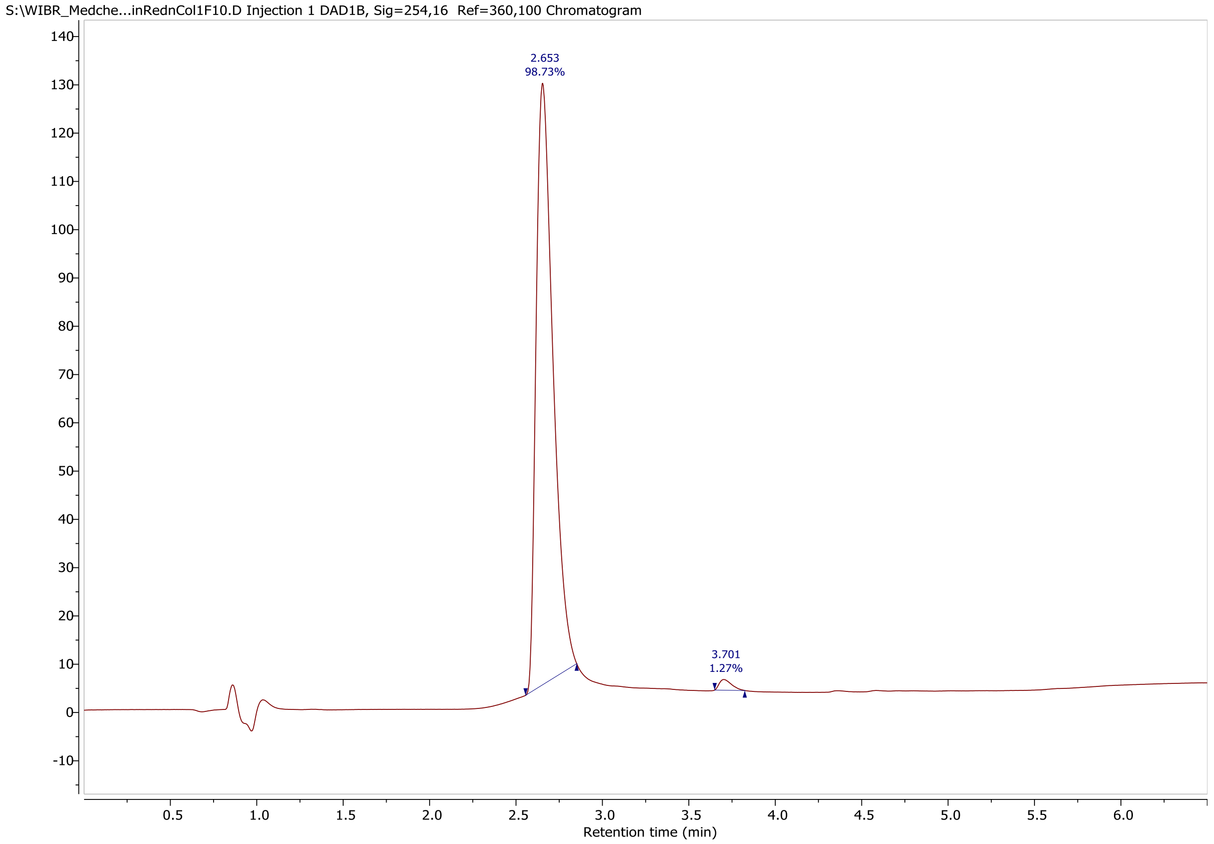

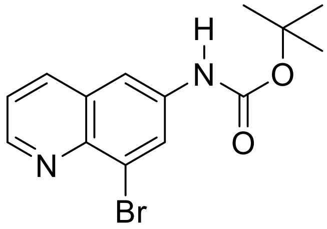

tert-butyl (8-bromoquinolin-6-yl)carbamate

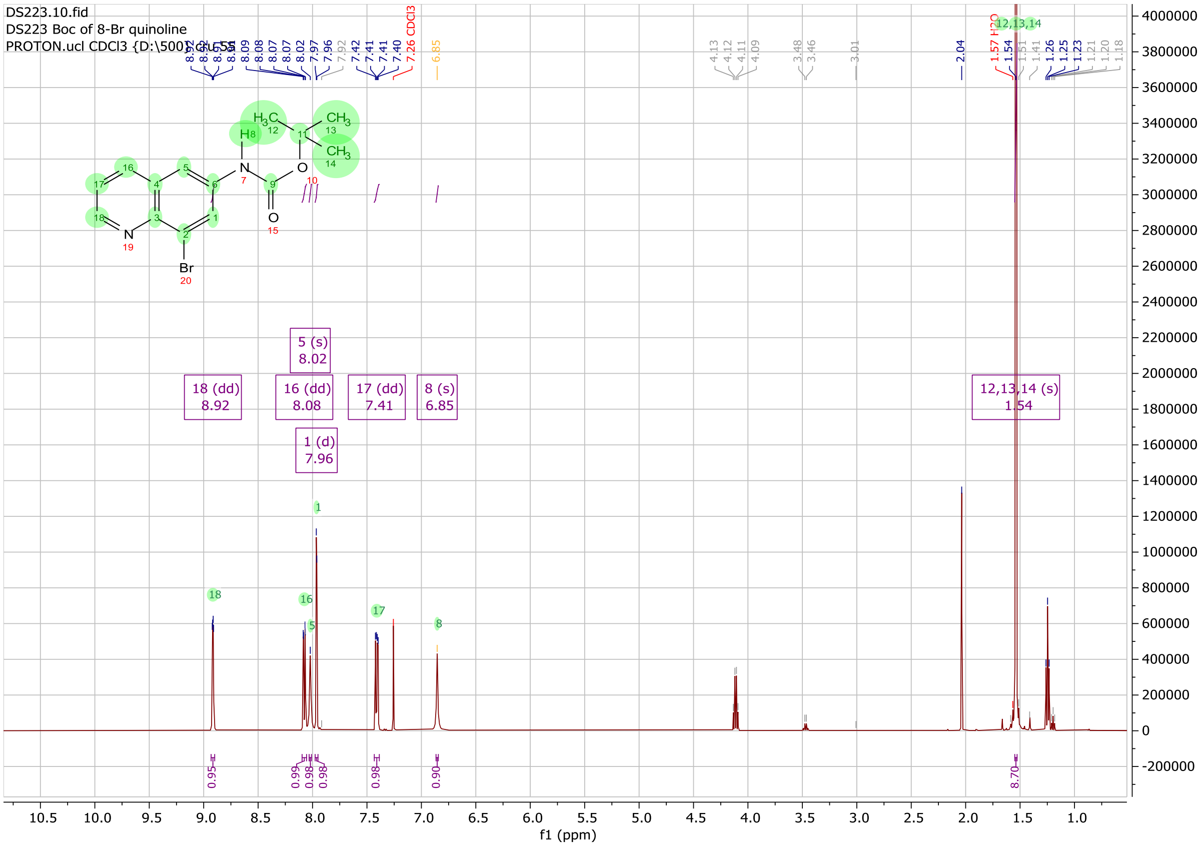

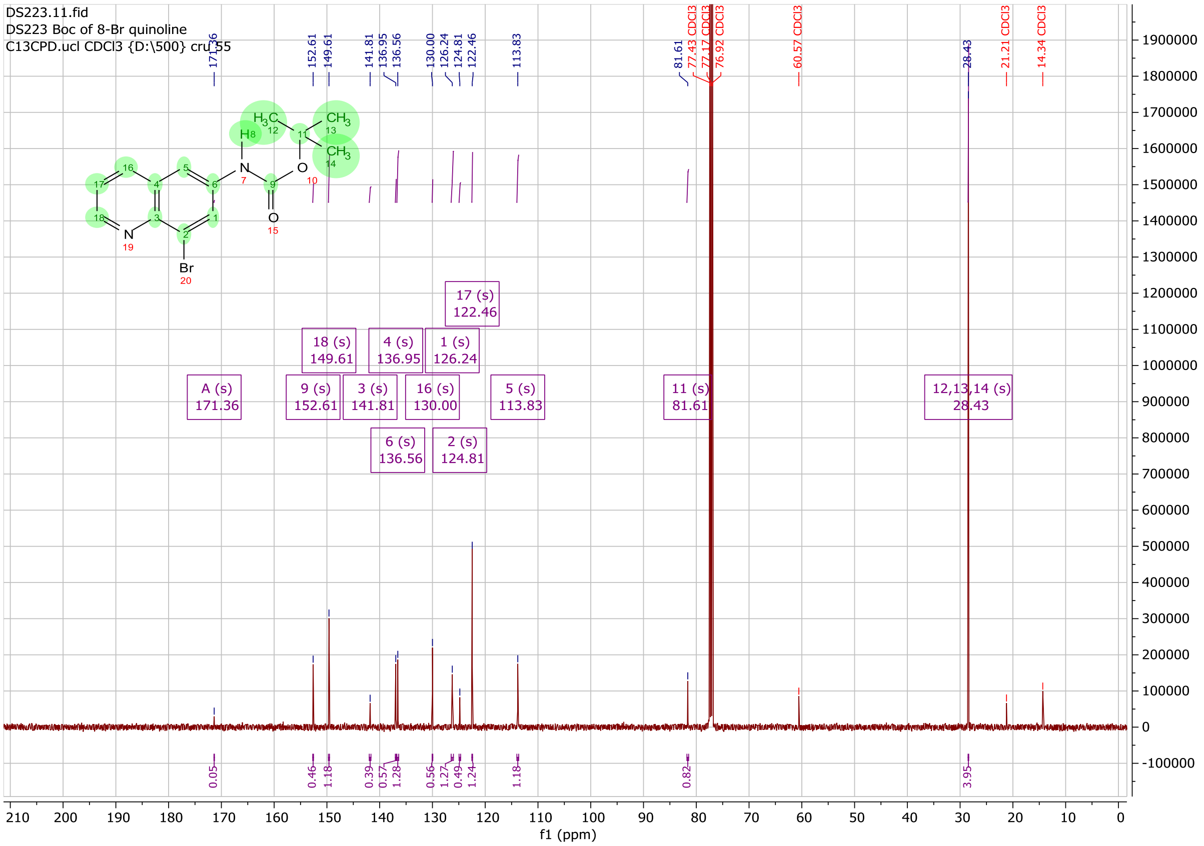

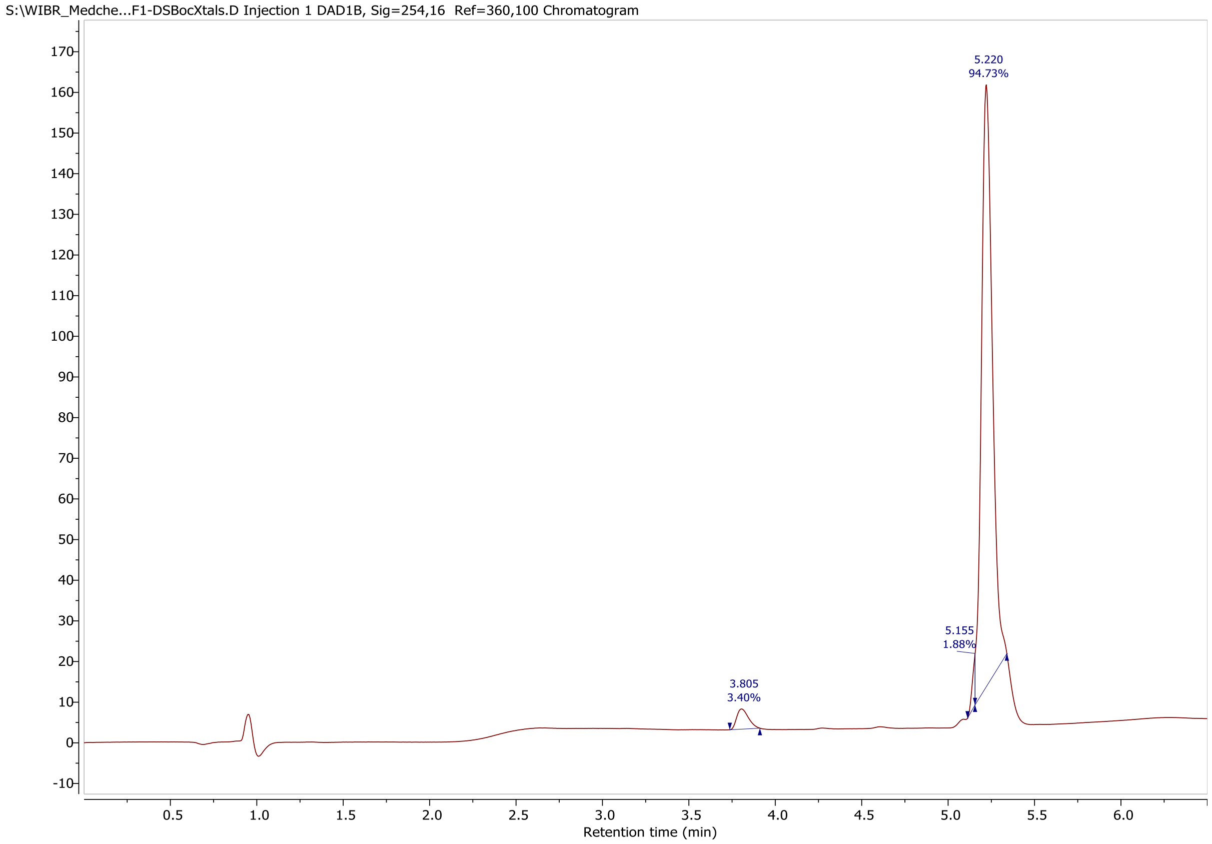

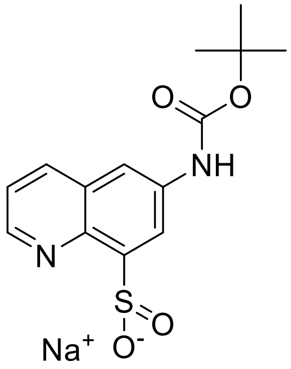

sodium 6-((tert-butoxycarbonyl)amino)quinoline-8-sulfinate

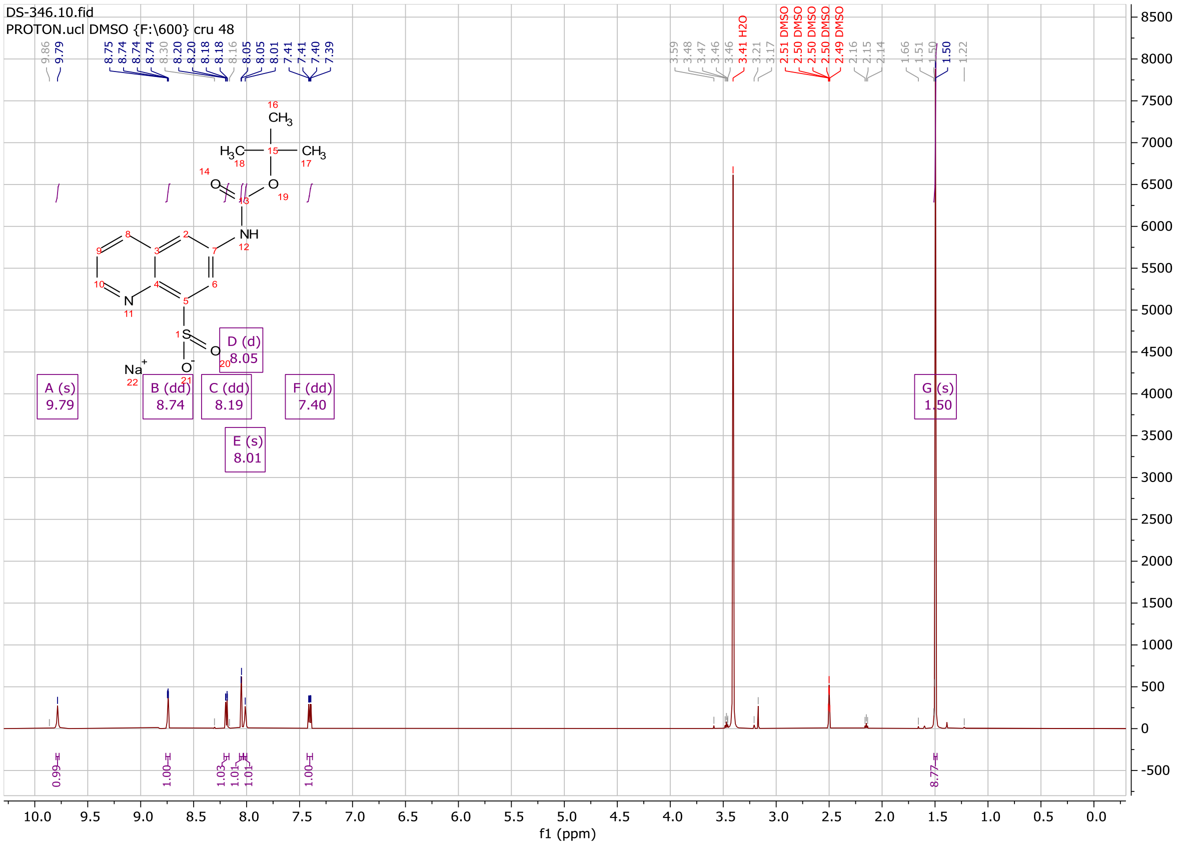

DS-346.10.fid
PROTON.ucl DMSO {F:\600} cru 48
A (s) 9.79
B (dd) 8.74
C (dd) 8.19
D (d) 8.05
E (s) 8.01
F (dd) 7.40
G (s) 1.50
3.41 H2O
2.51 DMSO
2.50 DMSO
2.49 DMSO
0.99
1.00
1.03
1.01
1.01
1.00
8.77
f1 (ppm)

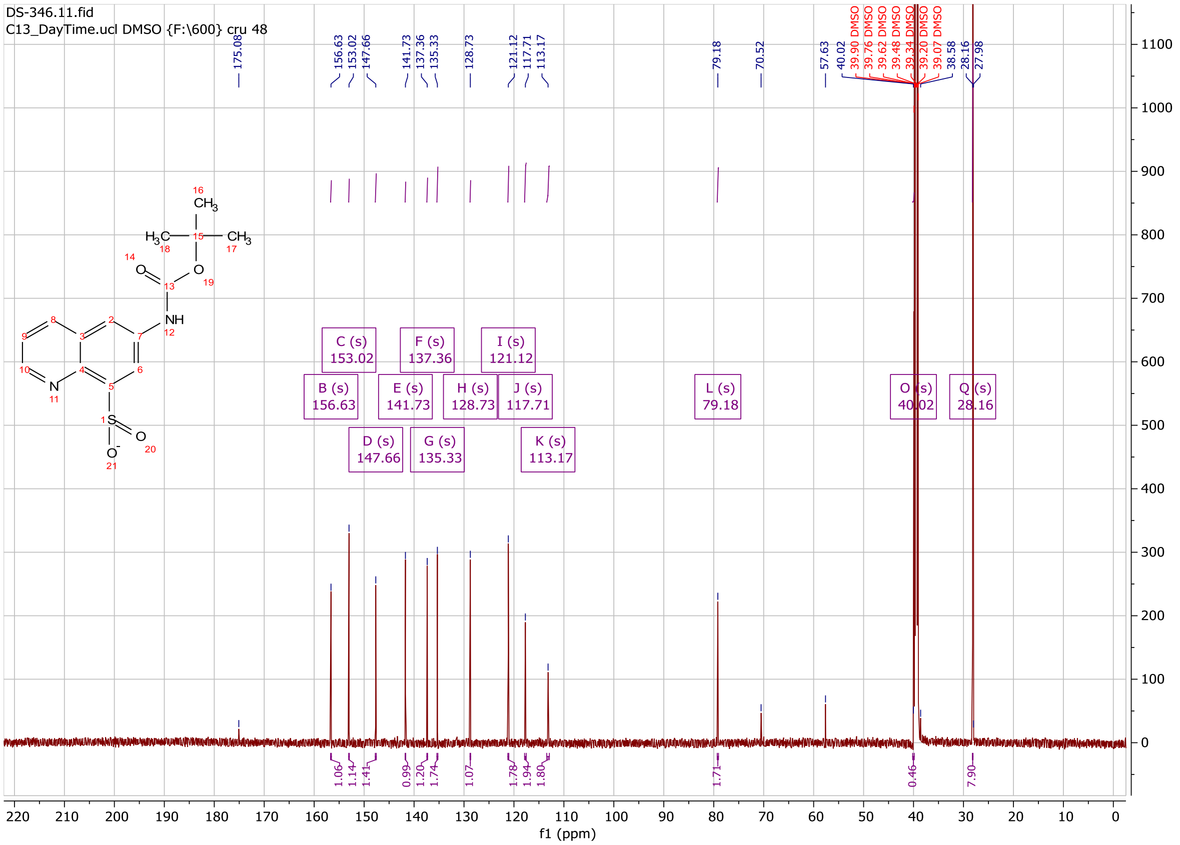
DS-346.11.fid
C13_DayTime.ucl DMSO {F:\600} cru 48
175.08
156.63
153.02
147.66
141.73
137.36
135.33
128.73
121.12
117.71
113.17
79.18
70.52
57.63
40.02
39.90 DMSO
39.76 DMSO
39.62 DMSO
39.48 DMSO
39.34 DMSO
39.20 DMSO
39.07 DMSO
38.58
28.16
27.98
B (s) 156.63
C (s) 153.02
D (s) 147.66
E (s) 141.73
F (s) 137.36
G (s) 135.33
H (s) 128.73
I (s) 121.12
J (s) 117.71
K (s) 113.17
L (s) 79.18
O (s) 40.02
Q (s) 28.16
1.06
1.14
1.41
0.99
1.20
1.74
1.07
1.78
1.94
1.80
1.71
0.46
7.90
f1 (ppm)

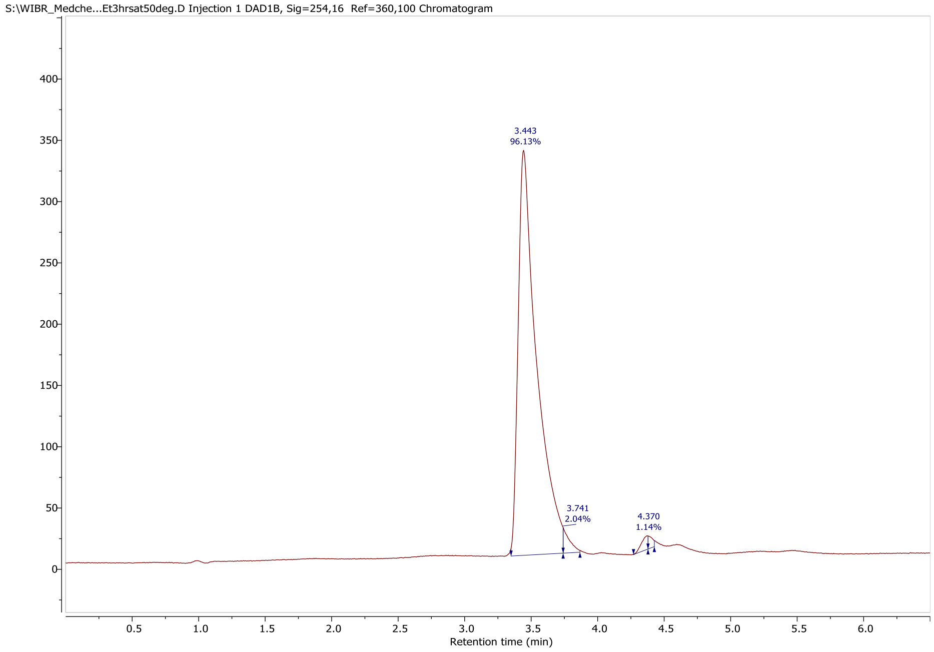
S:\WIBR_Medche...Et3hrsat50deg.D Injection 1 DAD1B, Sig=254,16 Ref=360,100 Chromatogram
3.443
96.13%
3.741
2.04%
4.370
1.14%
Retention time (min)

methyl 3-[[6-(tert-butoxycarbonylamino)-8-quinolyl]sulfonylamino]thiophene-2-carboxylate

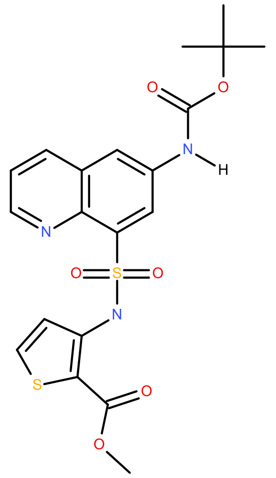

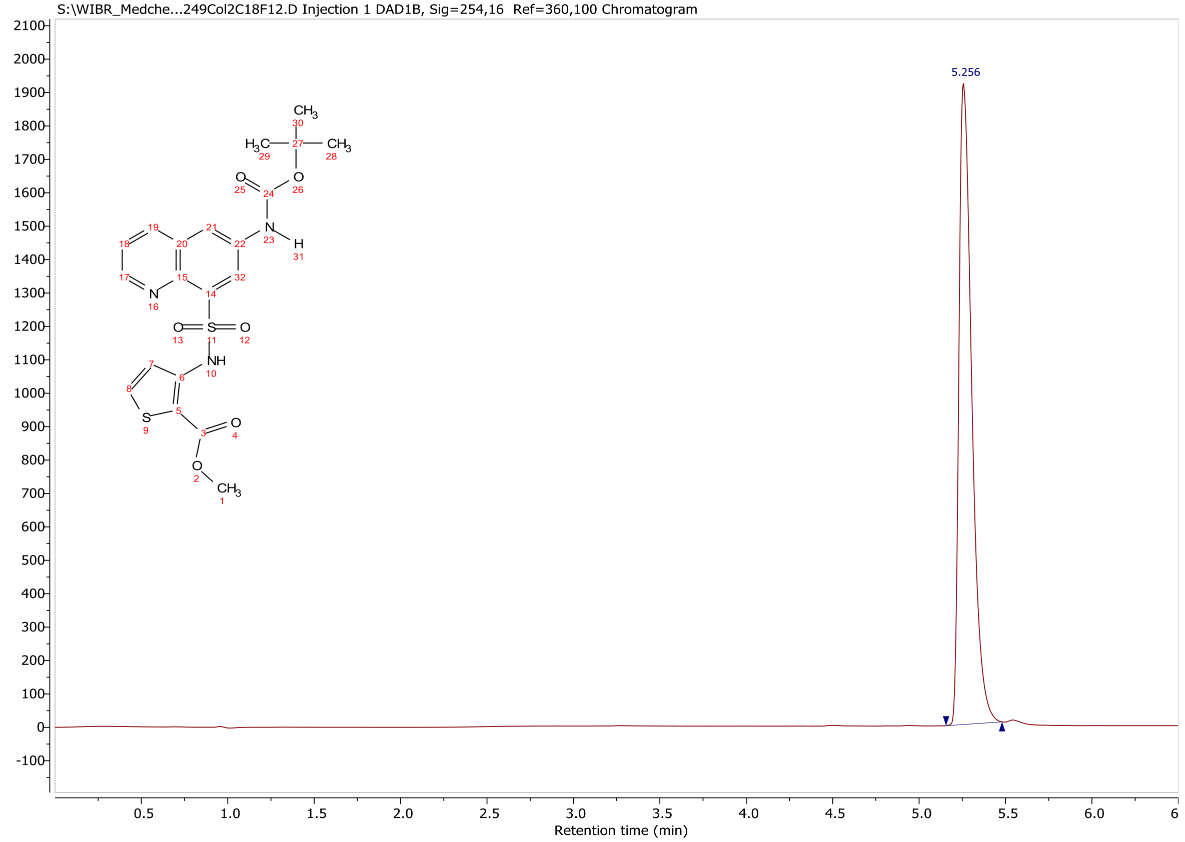

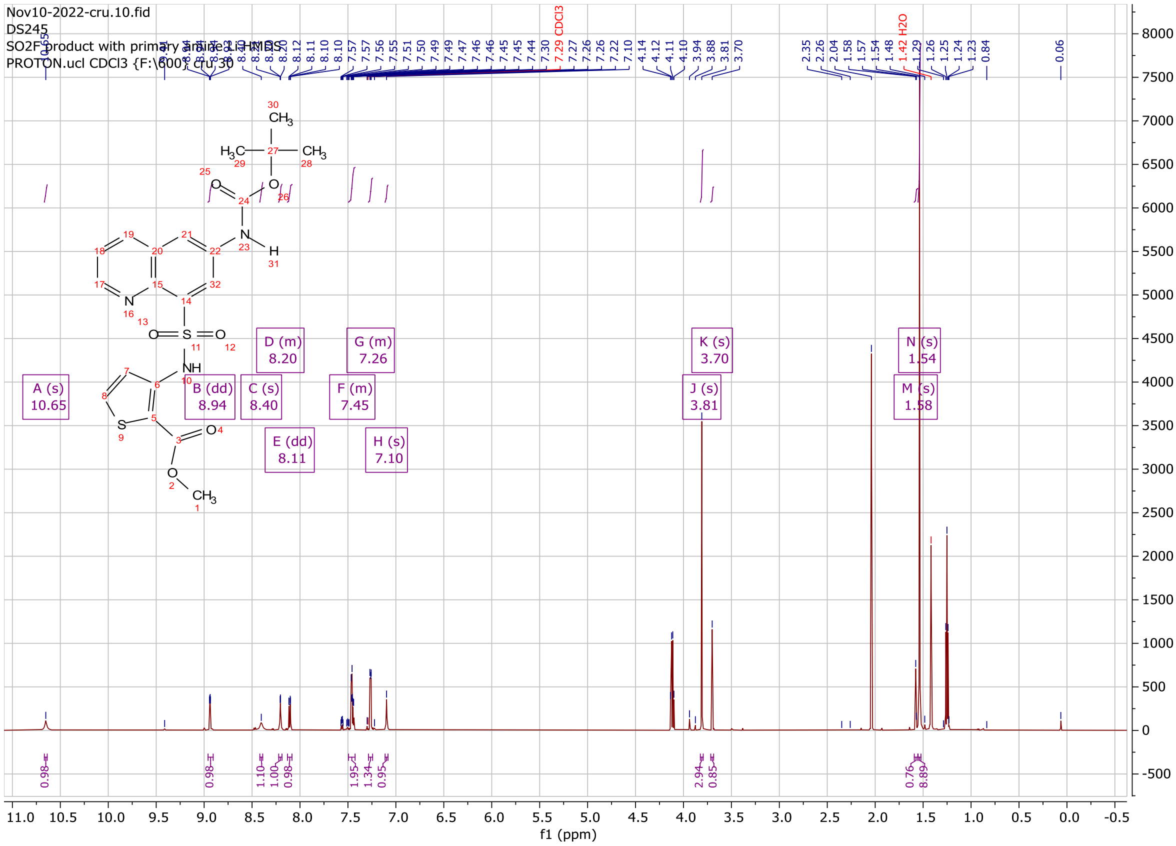

Nov10-2022-cru.10.fid
DS245
SO2F product with primary amine LiHMDS
PROTON.ucl CDCl3 {F:\600} cru 30
A (s) 10.65
B (dd) 8.94
C (s) 8.40
D (m) 8.20
E (dd) 8.11
F (m) 7.45
G (m) 7.26
H (s) 7.10
J (s) 3.81
K (s) 3.70
M (s) 1.58
N (s) 1.54
f1 (ppm)

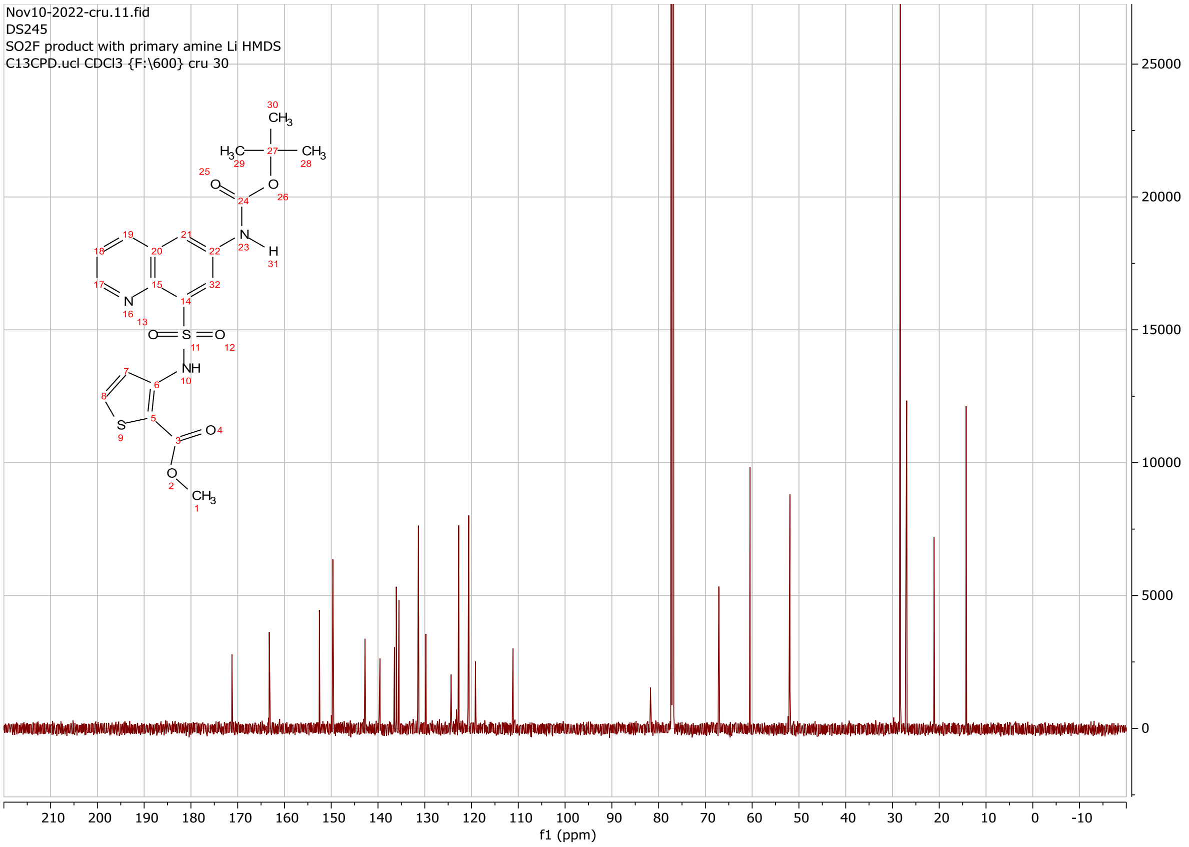

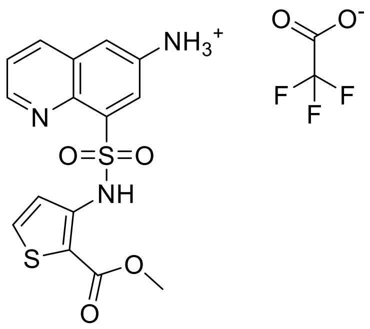

8-(N-(2-(methoxycarbonyl)thiophen-3-yl)sulfamoyl)quinolin-6-aminium 2,2,2-trifluoroacetate

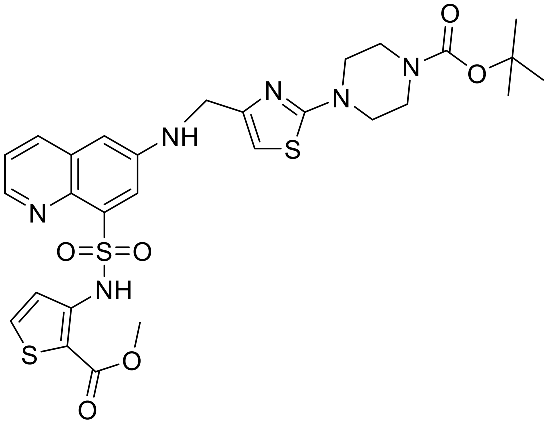

tert-butyl 4-(4-(((8-(N-(2-(methoxycarbonyl)thiophen-3-yl)sulfamoyl)quinolin-6-yl)amino)methyl)thiazol-2-yl)piperazine-1-carboxylate

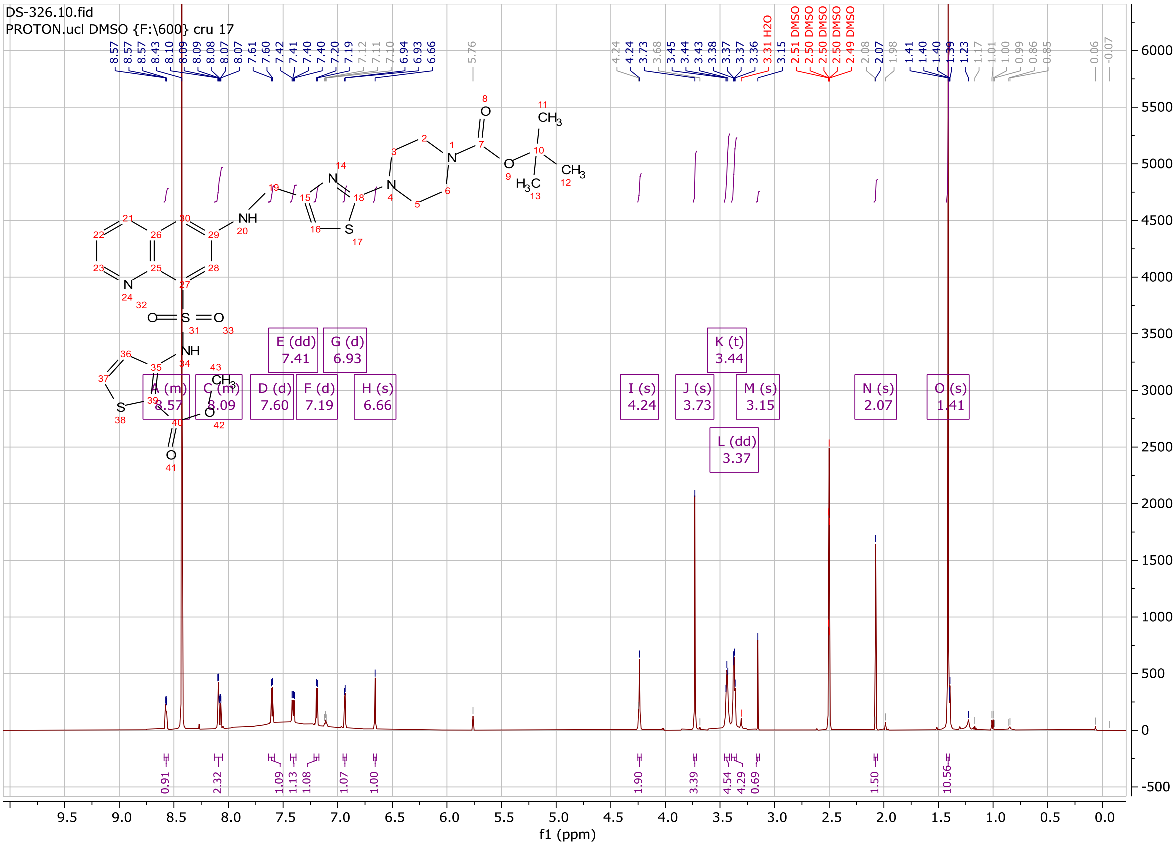

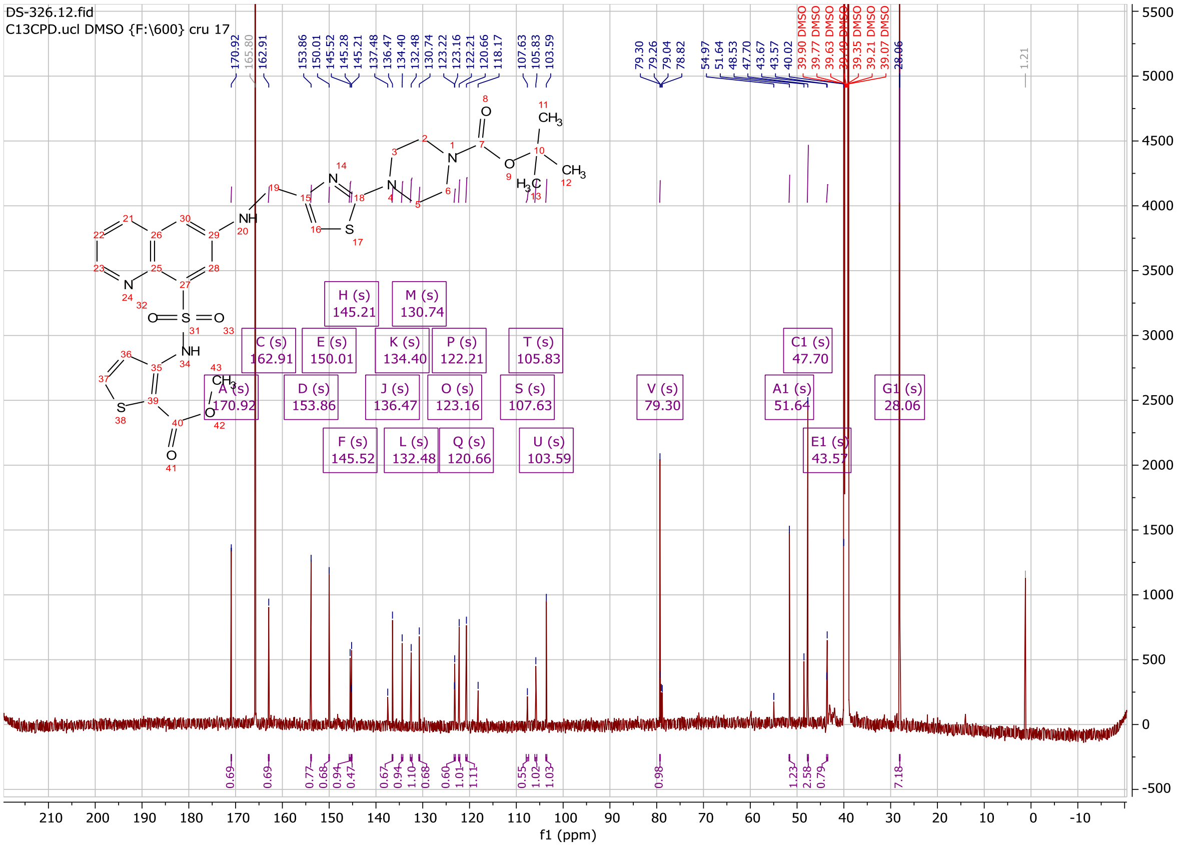
DS-326.12.fid
C13CPD.ucl DMSO {F:\600} cru 17
f1 (ppm)

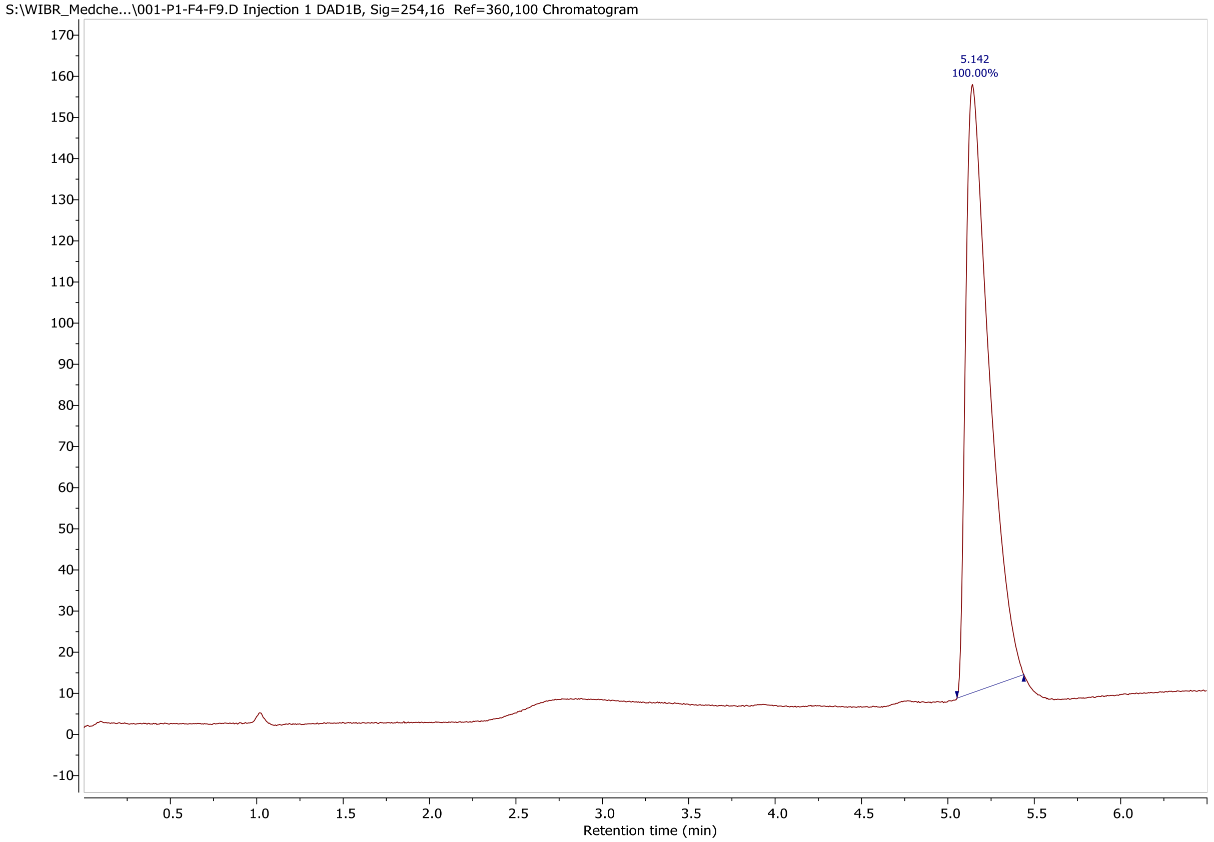
S:\WIBR_Medche...\001-P1-F4-F9.D Injection 1 DAD1B, Sig=254,16 Ref=360,100 Chromatogram
5.142
100.00%
Retention time (min)

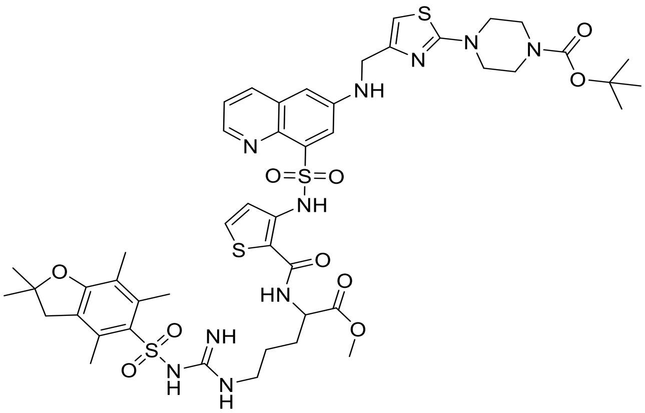

Methyl, N2-(3-((6-(((2-(4-(tert-butoxycarbonyl)piperazin-1-yl)thiazol-4-yl)methyl)amino)quinoline)-8-sulfonamido)thiophene-2-carbonyl)-Nw-((2,2,4,6,7-pentamethyl-2,3-dihydrobenzofuran-5-yl)sulfonyl)arginine

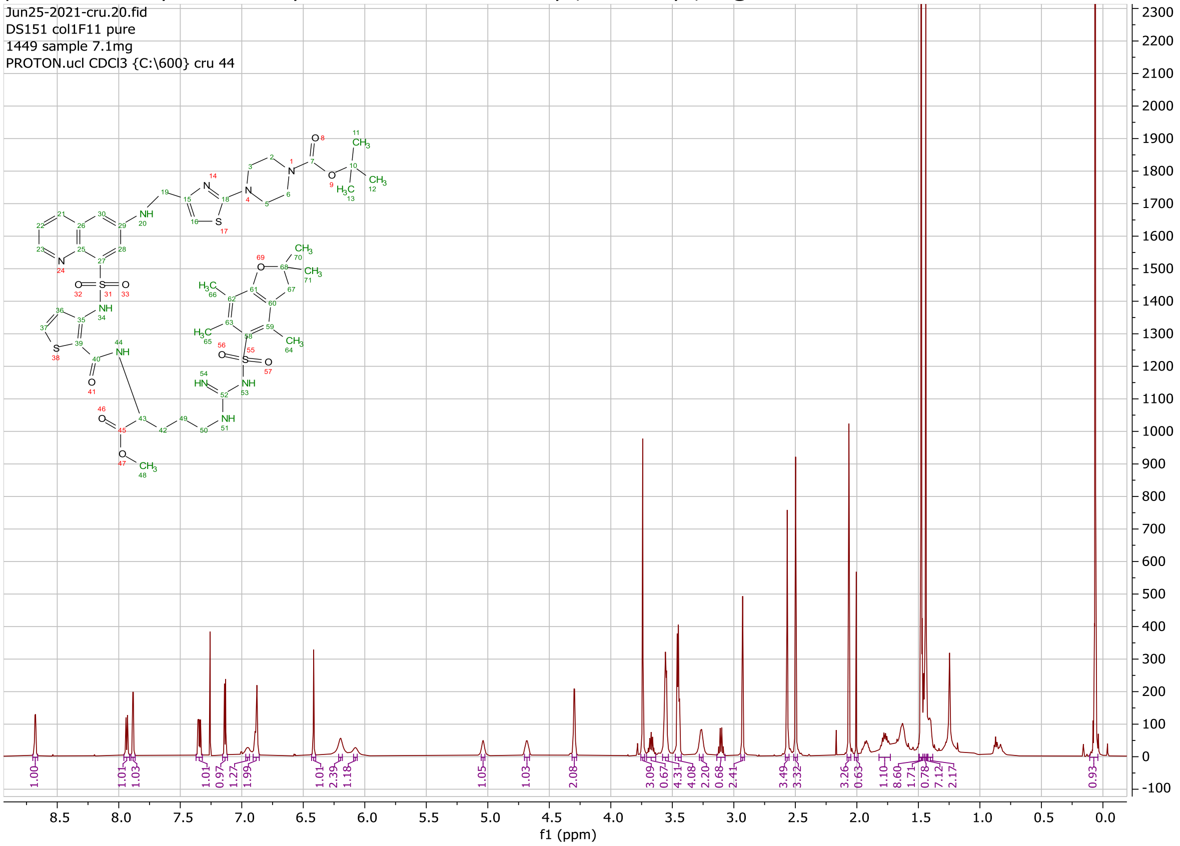

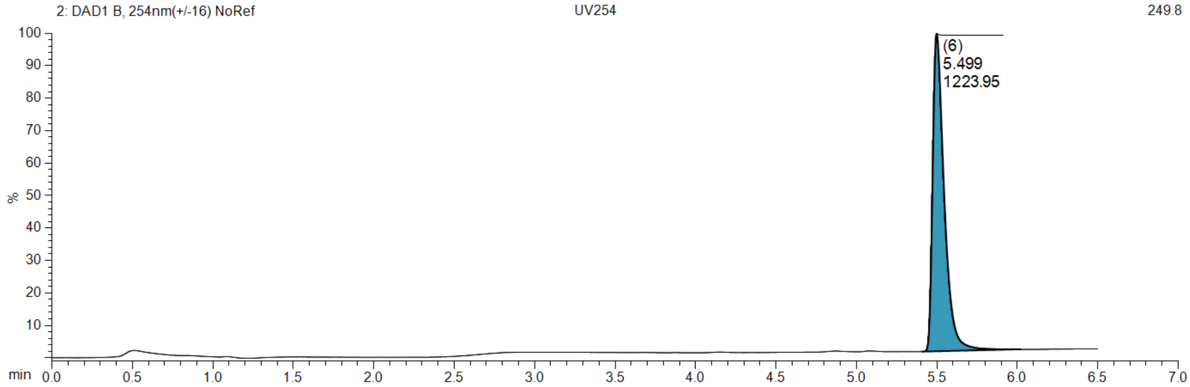

(2*S*)-5-guanidino-2-[[3-[[6-[(2-piperazin-1-ylthiazol-4-yl)methylamino]-8-quinolyl]sulfonylamino]thiophene-2-carbonyl]amino]pentanoic acid, formic acid 12h

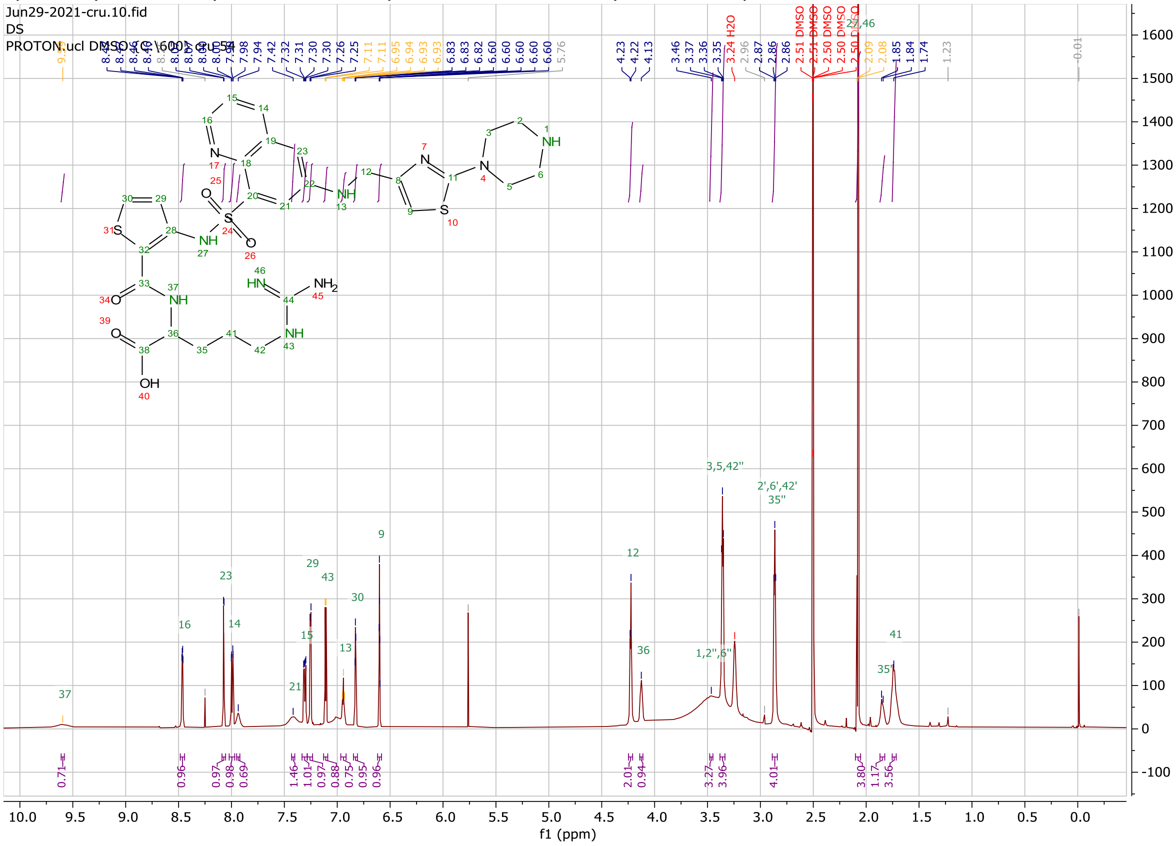

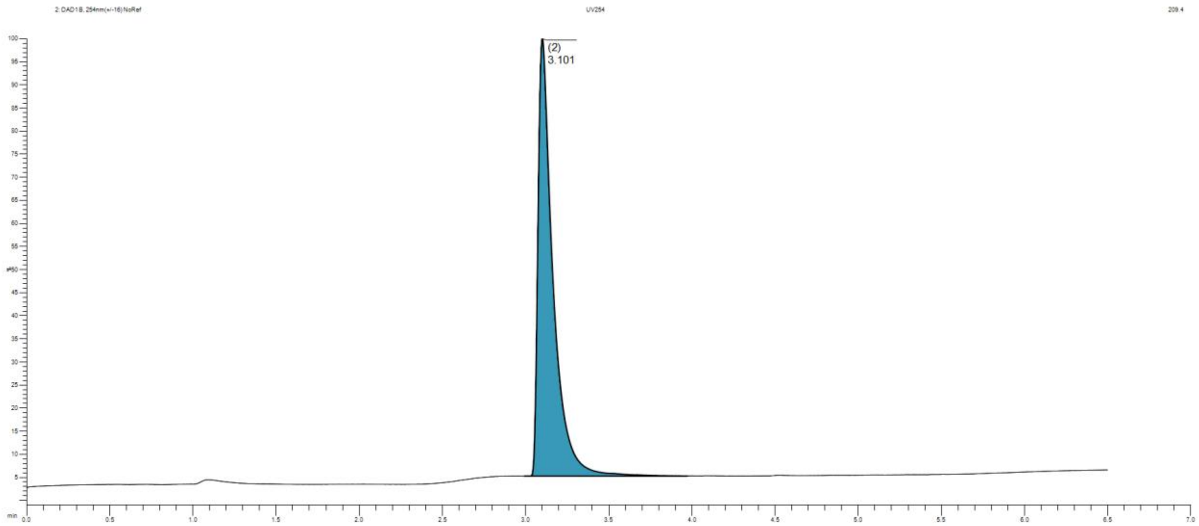
2: DAD1B, 254nm(+/-16) NoRef
UV254
209.4
(2)
3.101
min
